# Serum Biochemical Phenotypes in the Domestic Dog

**DOI:** 10.1371/journal.pone.0149650

**Published:** 2016-02-26

**Authors:** Yu-Mei Chang, Erin Hadox, Balazs Szladovits, Oliver A. Garden

**Affiliations:** 1 Research Office, The Royal Veterinary College, Camden Campus, Royal College Street, London, NW1 OTU, United Kingdom; 2 Department of Clinical Science and Services, Regulatory T Cell Laboratory, The Royal Veterinary College, Camden Campus, Royal College Street, London, NW1 OTU, United Kingdom; 3 Department of Pathology and Pathogen Biology, The Royal Veterinary College, Hawkshead Campus, North Mymms, Hatfield, Hertfordshire, AL9 7TA, United Kingdom; Oregon State University, UNITED STATES

## Abstract

The serum or plasma biochemical profile is essential in the diagnosis and monitoring of systemic disease in veterinary medicine, but current reference intervals typically take no account of breed-specific differences. Breed-specific hematological phenotypes have been documented in the domestic dog, but little has been published on serum biochemical phenotypes in this species. Serum biochemical profiles of dogs in which all measurements fell within the existing reference intervals were retrieved from a large veterinary database. Serum biochemical profiles from 3045 dogs were retrieved, of which 1495 had an accompanying normal glucose concentration. Sixty pure breeds plus a mixed breed control group were represented by at least 10 individuals. All analytes, except for sodium, chloride and glucose, showed variation with age. Total protein, globulin, potassium, chloride, creatinine, cholesterol, total bilirubin, ALT, CK, amylase, and lipase varied between sexes. Neutering status significantly impacted all analytes except albumin, sodium, calcium, urea, and glucose. Principal component analysis of serum biochemical data revealed 36 pure breeds with distinctive phenotypes. Furthermore, comparative analysis identified 23 breeds with significant differences from the mixed breed group in all biochemical analytes except urea and glucose. Eighteen breeds were identified by both principal component and comparative analysis. Tentative reference intervals were generated for breeds with a distinctive phenotype identified by comparative analysis and represented by at least 120 individuals. This is the first large-scale analysis of breed-specific serum biochemical phenotypes in the domestic dog and highlights potential genetic components of biochemical traits in this species.

## Introduction

The serum or plasma biochemical profile is a crucial tool in the diagnosis of systemic disease in veterinary medicine. Abnormal analytes may focus subsequent diagnostic efforts, such as imaging or serological tests, and monitoring of dogs may involve the submission of repeated biochemical profiles to assess the impact of treatment [1, 2]. Specific analytes may be of particular importance in certain disease setting. For example, the sodium: potassium ratio may be used as a screening tool for hypoadrenocorticism [3] and creatinine concentration provides the basis for the International Renal Interest Society (IRIS) staging of chronic kidney disease [4].

The biochemical profile requires optimal reference intervals for accurate interpretation, since clinical decision making is often influenced by the extent to which values depart from expected. However, most current reference intervals are based on reference values across several different types of breed of varying shape, size, age, sex, and neutering status. Age-related changes in biochemical analytes such as alkaline phosphatase (ALP) activity and concentrations of albumin, globulin, calcium, and phosphate have been recognized for some time [5–8]. In contrast, breed-specific phenotypes remain under-investigated, although certain breeds have more widely recognized biochemical characteristics that call into question the validity of common reference intervals, including greyhounds [9–11], Bernese mountain dogs [12] and Dogues de Bordeaux [13].

The dog is the most polymorphic terrestrial species on the planet. The Kennel Club recognizes over 200 different breeds in the United Kingdom, each defined by particular behavioral and physical characteristics that have been artificially selected by humans over the past 500 years [14]. The result is a high degree of interbreed heterogeneity, but remarkable intra-breed homogeneity [15]. Recent human genome-wide association studies have identified multiple loci associated with liver enzyme activity [16], and calcium [17] and magnesium [18] concentrations. Similar loci are likely to influence these and other biochemical analytes in dogs. Given the extent of inter-breed heterogeneity and intra-breed homogeneity of genetic diversity in dogs, breed-specific biochemical phenotypes are not only predictable but may also offer a powerful new approach to the genetic dissection of biochemical traits in health, with concurrent implications for a variety of diseases.

The objective of this study was therefore to perform the first large-scale retrospective analysis of phenotypic diversity of biochemical analytes by age, sex and breed in the domestic dog, both to assess the need for breed-specific reference intervals and to lay the foundations for the genetic dissection of these traits in the future, ultimately triangulating phenotype, breed and genetic predisposition.

## Materials and Methods

(Detailed protocols may be found in the S1 Materials and Methods; a summary of methods is presented here.)

### Inclusion and exclusion criteria

Serum biochemical profiles were collected from the database of the Royal Veterinary College (RVC) Diagnostic Laboratory (DL) from January 2004 to October 2013, comparing each dog’s profile to the generic reference intervals used by the DL for all dogs. Serum biochemical profiles were included in the study if every analyte fell within the reference interval, excluding glucose concentrations which were considered separately. Dogs without available glucose or if the glucose concentrations falling outside the reference interval were not considered as an exclusion criterion for the interpretation of the other biochemical analytes, but only those glucose concentrations falling within the reference interval were considered in the analysis of glucose. Data were collected from both pure breed dogs and a control group of mixed breed dogs (S1 Dataset), anonymizing all patient details in accordance with general guidelines laid down by the Royal Veterinary College Ethics and Welfare Committee.

### Specimen collection, analysis, and quality control

All blood samples were collected by licensed veterinarians, veterinary technicians, or veterinary students under supervision, for routine diagnostic purposes under the Veterinary Surgeons Act (1966), following written informed consent by the owners of the dogs. Venepuncture was performed in routine fashion, with minimal discomfort to individual animals. All samples were obtained for veterinary diagnostic, rather than experimental, purposes. The vast majority of blood samples were collected into plain serum tubes (99.8%) and analyzed using an IL600 Clinical Chemistry Analyzer (Instrumentation Laboratory Ltd; Cheshire, UK). Reported analytes included total protein, albumin, globulin, sodium, potassium, chloride, calcium, inorganic phosphorus, urea, creatinine, cholesterol, total bilirubin, alanine aminotransferase (ALT), ALP, creatinine kinase (CK), amylase, lipase and glucose.

### Statistical analysis

Age of the dogs was classified into eight mutually exclusive categories: less than or equal to one year of age (designated ≤1), greater than one year to less than or equal to two years of age (designated >1:≤2), and >2:≤4, >4:≤6, >6:≤8, >8:≤10, and >10 years of age. (For clarity of expression in the text of the Results and Discussion sections, these age categories are respectively labeled ≤1, 1‒2, 2‒4, 4‒6, 6‒8, 8‒10, and >10 years.) A linear mixed effects model (LMM) was used to assess the effect of age, sex, neutering status, and all two-way and three-way interactions on each analyte, taking breed as a random effect. Type I error rate was set at 5%. Adjusted means ± standard errors were presented. Residuals were defined as the observed values minus the estimated fixed effects of age, sex, and neutering status. For those breeds represented by at least 10 dogs, principal component analysis (PCA) was undertaken on all predicted random breed effects of the 18 biochemical analytes from the LMMs but the mixed breed dogs. Principal components with Eigenvalues greater than 1 were presented. Those breeds with at least one principal component of less than -2 or greater than +2 were considered to have distinctive phenotypes. A complementary analytical approach was then adopted to verify whether those breeds with distinctive phenotype based on PCA have significantly different distributions of biochemical analytes from outbred dogs with a heterogeneous genetic background. For each biochemical analyte the distributions of residuals for all breeds represented by at least 10 dogs were compared with those of the mixed breed dogs by two-sample Kolmogorov-Smirnov (KS) tests. To facilitate the pictorial representation of the data analyzed by KS tests, all breed codes submitted through the RVC Diagnostic Laboratory were classified into groups previously defined by haplotype analysis of single nucleotide polymorphisms (SNPs) [19], or to their closest matches (Tables A to R in S1 File), namely Ancient, Toy, Working, Sight Hound, Mastiff-like, Retriever/other Mastiff-like, Herding, Terrier, Scent Hound and Spaniel/Pointer groups. Those breeds not classified in this haplotype grouping and without an obvious nearest match were considered in a miscellaneous group labeled ‘Other’. Where significant differences were identified for breeds with at least 120 individuals, tentative breed-specific reference intervals were calculated based on ASVCP guidelines [20]. Normality of the analytes were assessed by the Kolmogorov-Smirnov tests and outliers assessed using Horn’s algorithm. Non-parametric methods were employed to establish the 95% reference intervals and the 90% confidence intervals of the lower and upper reference limits. The lower (upper) limit for the 95% breed-specific reference interval per analyte may be overestimated (underestimated) if more than 2% of the recorded data were equal to the existing RVC DL lower (upper) reference limit, thus suggesting ‘truncation’ of the data. This truncation was not applicable to the lower limit for total bilirubin since its value was already 0. All analyses were carried out using statistical software packages SAS 9.2 and R 3.1.2.

## Results

### Study population

Of 3045 normal serum biochemical profiles identified in the study period, 1495 had glucose concentrations within the reference interval. The study population included 311 intact females, 961 neutered females, 687 intact males and 1086 neutered males. Dogs ranged in age from four weeks to 18 years. Sixty breeds plus the mixed breed control group included 10 or more individuals, for which descriptive statistics are represented in Tables A to R in S1 File. Five breeds plus the mixed breed group included 120 or more individuals, including—in descending popularity—the Labrador Retriever (n = 327), Cavalier King Charles Spaniel (CKCS; n = 174), German Shepherd Dog (GSD; n = 160), Boxer (n = 146), and Golden Retriever (n = 121).

### Effects of age, sex and neutering status

**Fig 1:**
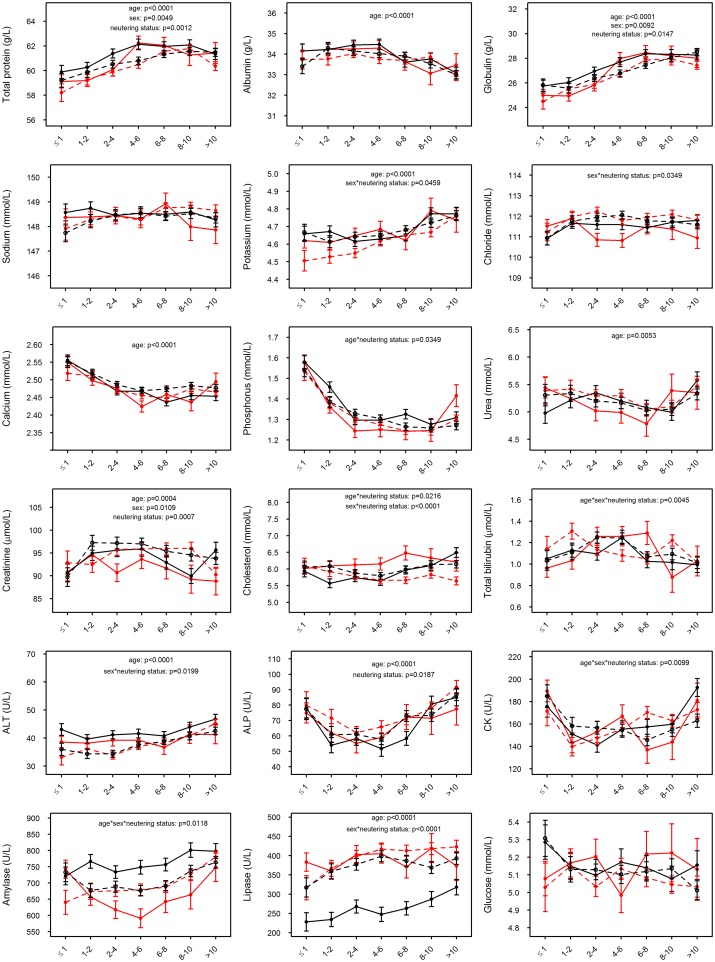
Effects of age, sex and neutering status on serum biochemical analytes. The adjusted mean values ± 1 standard error bars—accounting for breed—for each of the 18 biochemical parameters are represented on the respective *y* axes, showing age in years on the *x* axes. Intact male and female dogs are represented by the solid black and red lines, respectively; neutered male and female dogs are represented by the interrupted black and red lines, respectively. When present, significant differences (in age, sex, neutering status, or age*sex, age*neutering status, sex*neutering status or age*sex*neutering status interactions) are shown at the top of the figure; there was no significant difference in glucose between age, sex or neutering status. These analyses were all undertaken on the complete dataset of dogs, including both mixed and pure breeds.

All measured analytes except sodium (p = 0.4376), chloride (p = 0.0730) and glucose (p = 0.8430) varied with age (p<0.05; Fig 1, Table S in S1 File), though in some cases the differences were marginal despite being statistically significant. Total protein, globulin, potassium, ALT and lipase all generally increased with age (all analytes: p<0.0001), while other analytes showed a more variable pattern: albumin concentration (p<0.0001) was similar among younger dogs and decreased from 4–6 years of age; calcium (p<0.0001) and phosphorus (p<0.0001) concentrations were both higher among dogs ≤ 1 year of age and then decreased to a plateau from 2‒4 to >10 years of age; urea concentration (p = 0.0053) decreased from ≤ 1 to 6–8 years of age in all but the intact male dogs and then increased to >10 years of age in all gender groups; creatinine concentration (p = 0.0004) increased to 1–2 years of age and then decreased from 4–6 or, in the case of neutered female dogs, 8–10 to >10 years of age, except in the intact male dogs, which showed an increase from 8–10 to >10 years of age; and ALP activity (p<0.0001) decreased from ≤ 1 to 2–4 years of age and then gradually increased to levels similar to those ≤ 1 year of age. The effects of age on phosphorus, cholesterol, total bilirubin, CK and amylase depended on the sex and/or neutering status of the dogs, and for cholesterol and total bilirubin were marginal. Cholesterol concentration decreased with age in neutered female dogs, but in intact male dogs decreased from ≤1 to 1–2 years of age and then increased from 4–6 to >10 years of age; total bilirubin concentration increased from ≤1 to 2–4 years of age, remained at a plateau until 6–8 years of age, and then decreased again to >10 years of age in intact female dogs; CK activity decreased from ≤1 year of age to 2–4 years of age and then increased to >10 years of age in intact male dogs; and amylase activity decreased from ≤1 year of age to 4–6 years of age and then increased again to >10 years of age in intact female dogs, but in neutered female dogs increased from ≤1 year of age to >10 years of age.

There were no differences in albumin (p = 0.4579), sodium (p = 0.7232), calcium (p = 0.0722), phosphorus (p = 0.0692), urea (p = 0.6335), ALP (p = 0.2325) and glucose (p = 0.2833) between sexes, but concentrations of total protein (female 60.5±0.2 vs male 61.0±0.2 g/L, p = 0.0049), globulin (female 26.7±0.2 vs male 27.1±0.2 g/L, p = 0.0092) and creatinine (female 93±1 vs male 94±1 μmol/L, p = 0.0109) were all lower in female than male dogs, though the differences were generally marginal (Fig 1, Table S in S1 File). There were significant differences in total protein (neutered 60.5±0.2 vs intact 61.0±0.2 g/L, p = 0.0012), globulin (neutered 26.8±0.2 vs intact 27.1±0.2 g/L, p = 0.0147), creatinine (neutered 95±1 vs intact 93±1 μmol/L, p = 0.0007) and ALP (neutered 72±2 vs intact 67±2U/L, p = 0.0187) between neutered and intact dogs: in general, total protein and globulin concentrations were higher in intact than neutered dogs of each respective sex, while creatinine concentration and ALP activity were higher in neutered than intact dogs of each respective sex (Fig 1, Table S in S1 File).

There were significant interactions between sex and neutering status for potassium (p = 0.0459), chloride (p = 0.0349), cholesterol (p<0.0001), ALT (p = 0.0199) and lipase (p<0.0001), although in all cases except lipase the differences were marginal. In general, potassium concentrations were higher in intact (4.67±0.02 mmol/L) than neutered female (4.61±0.02 mmol/L), but not male, dogs; chloride concentrations were lower in intact (111.3±0.2 mmol/L) than neutered female (111.9±0.1 mmol/L), but not male, dogs; cholesterol concentrations were higher in intact (6.2±0.1 mmol/L) than neutered female (5.8±0.1 mmol/L), but not male, dogs; and ALT activities were higher in intact (42±1 U/L) than neutered male (38±1 U/L), but not female, dogs. However, lipase activities were notably lower in intact (264±11 U/L) than neutered male dogs (371±10 U/L), but there were no differences in values for intact and neutered female dogs.

There were significant three-way interactions between age, sex and neutering status for total bilirubin (p = 0.0045), CK (p = 0.0099) and amylase (p = 0.0188), although once again the differences were marginal in all but amylase: intact male dogs had notably higher amylase activity than intact female dogs from 1–2 to 6–8 years of age, while values for neutered male and female dogs, which could not be distinguished, were intermediate in magnitude.

The observations recorded in this section relate to the complete dataset of dogs, including both mixed and pure breeds. However, broadly similar trends were observed when the most populous breed—the Labrador Retriever (n = 327)–was examined independently (S1 Fig).

### Principal component analysis identifies breed-specific serum biochemical phenotypes

**Fig 2:**
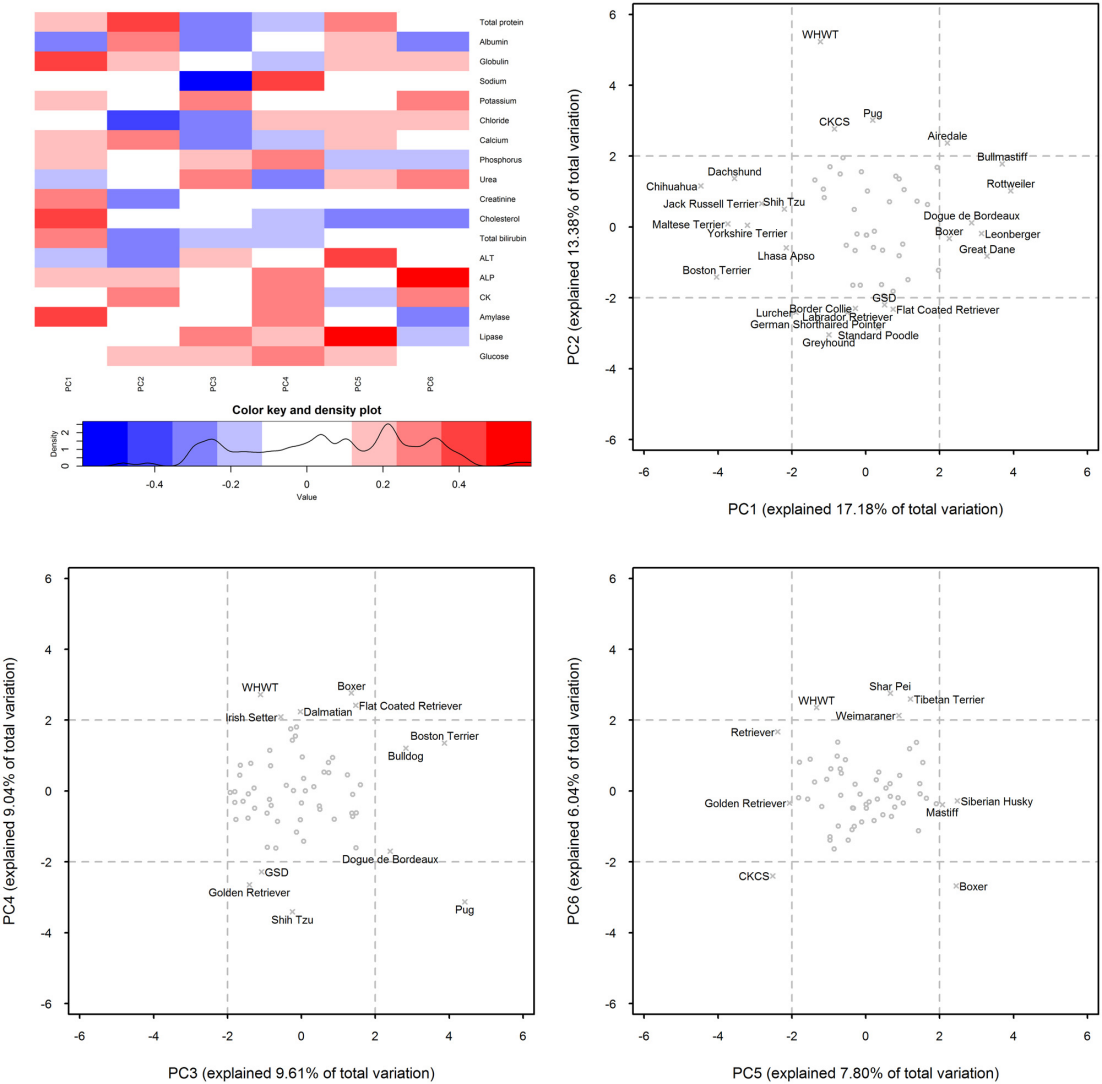
Principal component analysis identifies breed-specific serum biochemical phenotypes. Of the 18 principal components identified in the current dataset, the first six had Eigenvalues of over 1 and cumulatively accounted for 63% of the total variance. A heat map showing correlations between the serum biochemical parameters and the first six principal components (PC1 to PC6) is shown in part (a). Biplots (PC1 *vs* PC2; PC3 *vs* PC4; PC5 *vs* PC6) are shown in part (b), revealing outlying breeds with principal components of greater than +2 and less than -2, representing distinctive phenotypes. These outlying breeds are labeled in the biplots.

Eighteen principal components were identified in the current dataset, of which the first six had Eigenvalues of greater than 1 (Fig 2A), cumulatively accounting for 63% of the total variance of the dataset (Table T in S1 File). The first two principal components identified 26 breeds with distinctive phenotypes; the inclusion of principal components 3 to 6 added an additional 10 breeds to this list. Thus, a total of 36 of 60 breeds showed distinctive phenotypes on the basis of the first six principal components (Fig 2B). There were 10 breeds with distinctive phenotypes on the basis of principal component 1 only, six on the basis of principal component 2 only, one on the basis of principal component 3 only, two on the basis of principal component 4 only, and three each on the basis of principal component 5 or 6 (Fig 2B). Eleven breeds had distinctive phenotypes on the basis of multiple principal components. In particular, the boxer had a distinctive phenotype on the basis of principal components 1, 4, 5 and 6; the pug, on the basis of principal components 2, 3 and 4; the West Highland White Terrier (WHWT), on the basis of principal components 2, 4 and 6; the CKCS, on the basis of principal components 2, 5 and 6; the Shih Tzu, on the basis of principal components 1 and 4; and the Golden Retriever, on the basis of principal components 4 and 5; Airedale on the basis of principal components 1 and 2; both Flat-coated Retriever and the GSD on the basis of principal components 2 and 4; Boston Terrier and Dogue de Bordeaux on the basis of principal components 1 and 3.

### Comparisons with a control group of mixed breed dogs confirm breed-specific phenotypes

**Fig 3:**
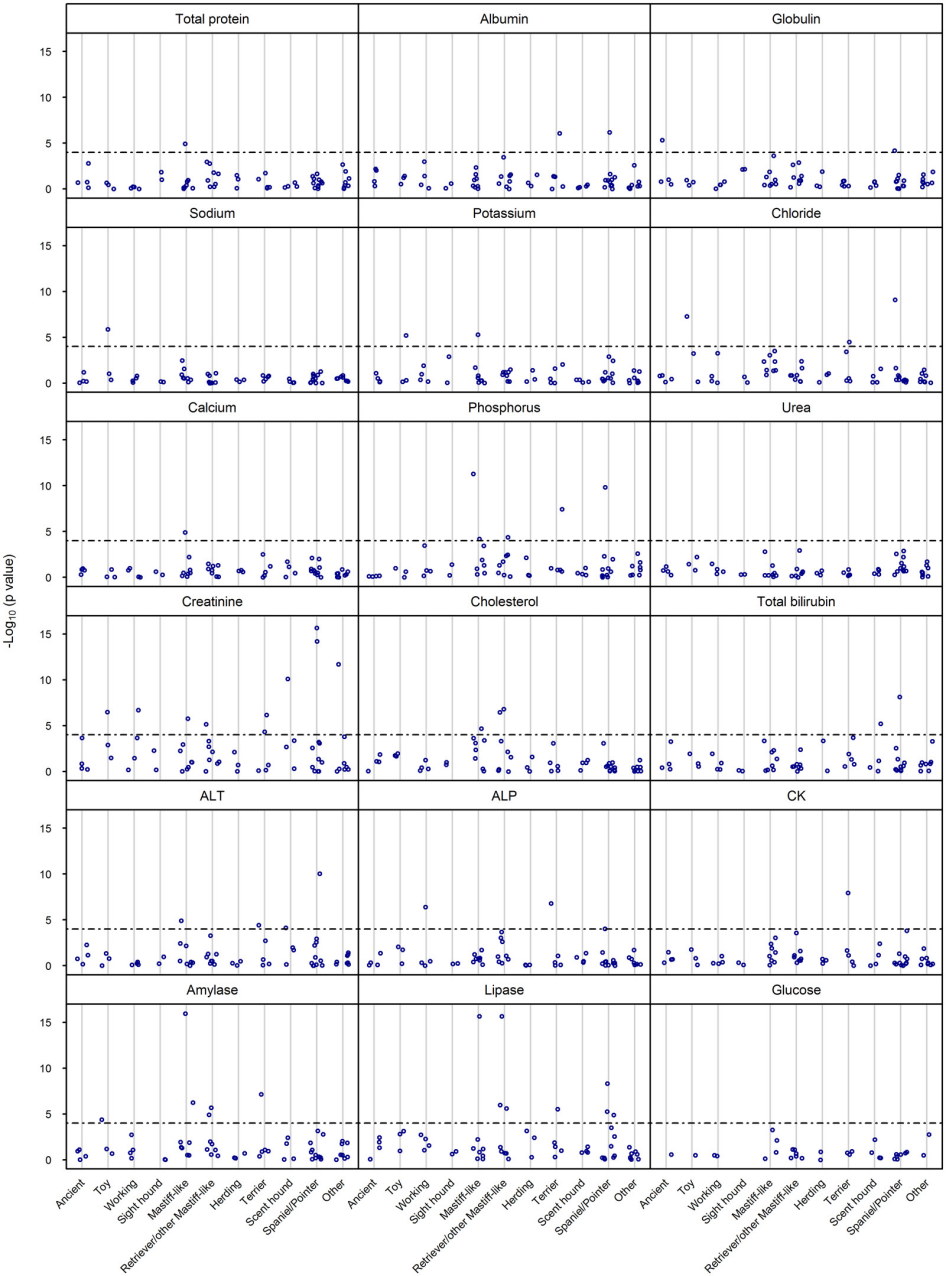
Pairwise comparisons of serum biochemical analytes between pure breed and mixed breed dogs confirm breed-specific phenotypes. The results of two-sample Kolmogorov-Smirnov tests to compare the distributions of residuals for the pure breed *versus* mixed breed dogs (Residuals were defined as the observed values minus the estimated fixed effects of age, sex and neutering status). Respective *y* axes show -log_10_ (p value) of specific individual breeds assigned to one of 11 genetically-related groups, shown on the *x* axes. A Bonferroni correction yielded a threshold for significance of 10^−4^, represented by the interrupted line. All breeds with residual values significantly different from those of the mixed breed population are shown above the line.

**Fig 4:**
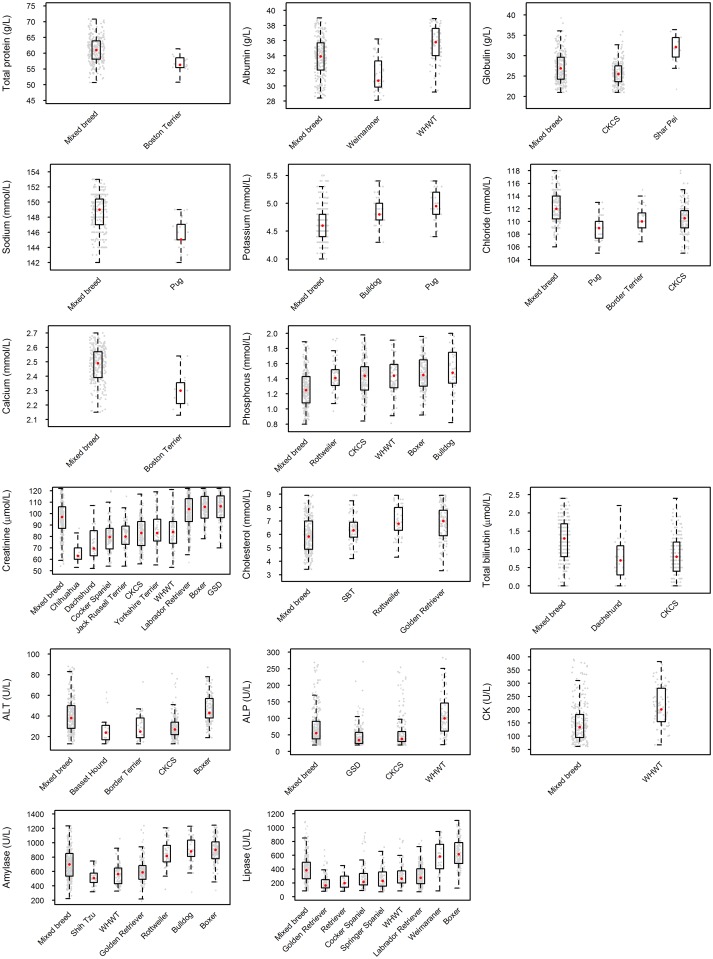
Combined box-and-whisker / dot plots representing serum biochemical data from breeds showing a significant difference from the mixed breed group for each of the 16 parameters for which differences were documented. Each small dot represents an individual dog, the boxes show the respective 25^th^ and 75^th^ percentiles, the larger red dots median values, and the whiskers the lowest and highest data points still within 1.5 times the interquartile range of the respective lower and upper quartiles.

All biochemical analytes except urea and glucose showed significant differences between specific individual breeds and the mixed breed group (Fig 3; Table U in S1 File). Notable observations included the differences between pure and mixed breed dogs in total protein concentration in the Mastiff-like group (Boston Terrier); differences in albumin concentration in the Terrier (WHWT) and Spaniel/Pointer (Weimaraner) groups; differences in globulin concentration in the Ancient (Shar Pei) and Spaniel/Pointer (CKCS) groups; differences in sodium concentration in the Toy group (Pug); differences in potassium concentration in the Toy (Pug) and Mastiff-like (Bulldog) groups; differences in chloride concentration in the Toy (Pug), Terrier (Border Terrier) and Spaniel/Pointer (CKCS) groups; difference in calcium concentration in the Mastiff-like group (Boston Terrier); differences in inorganic phosphorus concentration in the Mastiff-like (Boxer, Bulldog), Retriever/other Mastiff-like (Rottweiler), Terrier (WHWT) and Spaniel/Pointer (CKCS) groups; differences in creatinine concentration in the Toy (Chihuahua), Working (GSD), Mastiff-like (Boxer), Retriever/other Mastiff-like (Labrador Retriever), Terrier (WHWT, Yorkshire Terrier), Scent hound (Dachshund), Spaniel/Pointer (Cocker Spaniel) and Other (Jack Russell Terrier) groups; differences in cholesterol concentration in the Mastiff-like (Staffordshire Bull Terrier (SBT)) and Retriever/other Mastiff-like (Rottweiler, Golden Retriever) groups; differences in total bilirubin concentration in the Scent hound (Dachshund) and Spaniel/Pointer (CKCS) groups; differences in ALT activity in the Mastiff-like (Boxer), Terrier (Border Terrier), Scent hound (Basset Hound) and Spaniel/Pointer (CKCS) groups; differences in ALP activity in the Working (GSD), Terrier (WHWT) and Spaniel/Pointer (CKCS) groups; differences in CK activity in the Terrier group (WHWT); differences in amylase activity in the Toy (Shih Tzu), Mastiff-like (Boxer, Bulldog), Retriever/other Mastiff-like (Rottweiler, Golden Retriever) and Terrier (WHWT) groups; and differences in lipase activity in the Mastiff-like (Boxer), Retriever/other Mastiff-like (Labrador Retriever, Golden Retriever and Retriever), Terrier (WHWT) and Spaniel/Pointer (Cocker and Springer Spaniel, Weimaraner) groups (Fig 4).

Breeds with three or more biochemical differences from the mixed breed group were the Pug (sodium, potassium, chloride), Bulldog (potassium, phosphorus, amylase), Boxer (phosphorus, creatinine, ALT, amylase, lipase), Golden Retriever (cholesterol, amylase, lipase), Rottweiler (phosphorus, cholesterol, amylase), WHWT (albumin, phosphorus, creatinine, ALP, CK, amylase, lipase), and CKCS (globulin, chloride, phosphorus, creatinine, total bilirubin, ALT, ALP).

S2 Fig summarizes the results of PCA and pairwise comparisons with the control mixed breed group, showing that the majority of breeds highlighted by comparative analysis (18/23) were also identified by PCA, but that 50% (18/36) of the breeds were identified by PCA alone.

### Towards breed-specific serum biochemical reference intervals

Of the 23 breeds identified by pairwise comparisons with the mixed breed group, five were represented by at least 120 dogs (Labrador Retriever, CKCS, GSD, Boxer, Golden Retriever). Histograms of the serum biochemical profiles were presented in S3 to S7 Figs. Normality of the analytes were rejected for globulin (Labrador Retriever), sodium (Labrador Retriever, GSD, CKCS), potassium (Labrador Retriever, CKCS), chloride (Labrador Retriever, Boxer), urea (Labrador Retriever, Boxer), creatinine (Labrador Retriever, GSD), total bilirubin (Labrador Retriever), amylase (Golden Retriever), lipase (Labrador Retriever, Golden Retriever); ALT, CK and ALP did not followed Gaussian distributions for all 5 breeds. Multiple outliers were detected for chloride (Labrador Retriever, CKCS, GSD, Boxer) and amylase (CKCS, GSD, Golden Retriever). One or two outliers were detected for globulin, potassium, ALT and ALP. Tentative breed-specific reference intervals were calculated for these breeds (Tables V to Z in S1 File).

## Discussion

To the best of the authors’ knowledge, the current study represents the largest retrospective review of normal serum biochemical profiles in the dog. Data from over 3000 individuals representing 60 pure breeds plus a mixed breed control population were examined, revealing effects attributable to age, sex, neutering status and breed. Two complementary analytical strategies, PCA and pairwise comparisons of every pure breed with the control population, demonstrated distinct breed-specific serum biochemical phenotypes, adding to the growing body of literature highlighting the physical, behavioral and physiological diversity of this polymorphic species [21, 22].

Age, sex and neutering status variably impacted many of the serum biochemical analytes. Serum calcium, phosphorus and ALP were higher in younger dogs, as previously published in a number of species [13, 23–28], thought to reflect the impact of increased concentrations of growth hormone on bone turnover and renal phosphate reabsorption [29], an effect that was apparent until 2–4 years of age in this study. A number of novel observations were also made. Serum globulin concentration showed a sustained increase up to 6–8 years of age, coinciding with a decrease in serum albumin from 4–6 years of age. Comprising both acute phase proteins and gamma globulins, we speculate that the rising globulin concentration reflected a progressively increasing inflammatory burden with age, coined ‘inflammaging’ [30, 31]. A concurrent blunting of hepatic synthesis of albumin, a negative acute phase protein, alongside subclinical renal and/or intestinal losses of this protein could have accounted for the decreasing concentration of this analyte, an observation also made in Dogues de Bordeaux [32] and elderly people in whom albumin concentrations less than 40g/l, but still reported as normal, are associated with increased risk of mortality [33, 34]. Though small in magnitude, serum urea and creatinine showed variable changes, creatinine generally increasing and then decreasing with age, thought to reflect growth of muscles to maturity and then age-related sarcopenia; and urea decreasing to 6–8 years of age and then increasing to >10 years of age, thought to reflect changes in dietary protein intake and declining renal function or subclinical dehydration with age. The lack of an increase in creatinine with advancing age in all but the intact male dogs was intriguing and at odds with changes in serum creatinine in the aging human population [35, 36], but concordant with recent data in aging healthy dogs [32, 37]. From 4–6 years of age, serum ALT and ALP activities both increased, possibly also reflecting a rising subclinical inflammatory burden of the liver with age, one source of which could have been the aging gastrointestinal tract [38]. However, this observation was at odds with a study of Dogues de Bordeaux, which revealed a decrease in serum ALT activity with age [32]. Furthermore, whilst we were careful to include only biochemical profiles from dogs in which every analyte was within existing reference intervals, unhealthy individuals would inevitably have contributed to the sample pool. Unfortunately, not all blood samples were associated with clear information on why the samples were submitted, precluding a systematic analysis of this possibility. Pre-existing disease and the administration of medications, including corticosteroids, could therefore have contributed to the rise in activity of these analytes. Additional findings included the higher CK activity in intact male dogs <1 and >10 years of age, the former observed as a general phenomenon in other species and attributed to growth and heightened activity of young animals [23, 27], and the latter thought to reflect low-level inflammation and the increasingly recumbent lifestyle of older dogs. The rising potassium concentration was also notable and likely to reflect the combined influence of age on absorption, excretion and transcellular shifts; similar observations have been made in human patients [36, 39].

Sex and neutering status were additional confounding factors that we considered in our analysis. Subtle differences between the sexes were seen in total protein, globulin and creatinine, all of which were higher in male than female dogs. In the case of creatinine, this was thought to reflect the generally larger size and muscle bulk of male dogs, but in the case of globulin and total protein this was a novel finding that we speculate represented the impact of sex hormones, especially androgens, on protein turnover. In contrast to total protein and globulin, creatinine and ALP were higher in neutered than intact dogs of either sex, presumably reflecting diminished clearance of these analytes in the neutered state. More striking differences between the sexes were seen in amylase and lipase, which appeared to be reciprocally influenced by male sex hormones, intact male dogs displaying the highest amylase but the lowest lipase activity. The lower lipase activity in intact male dogs was at odds with a study in silver foxes that showed males had higher serum lipase activity than females [40], suggesting species differences in sex hormone modulation of this enzyme. In contrast to lipase, amylase also appeared to be influenced by female sex hormones, intact female dogs displaying the lowest activity of this enzyme. Reconciling with this observation, estradiol was shown to decrease pancreatic amylase concentration in guinea pigs [41]. However, in a more recent study in rats estradiol was necessary to maintain amylase content in the pancreas [42], again suggesting species-specific hormonal influences. To the best of our knowledge, this is the first time that gender influences on serum activities of these enzymes have been documented in dogs, reconciling with known gender differences of several analytes in a number of species [43, 44]. Such observations underline the need to account for sex as well as breed in assessing what is normal.

Adopting a similar approach to that of our recent study of canine hematological phenotypes [45], two complementary analytical techniques were employed to identify distinctive serum biochemical phenotypes with breed, namely PCA of all of the data from pure breed dogs and pairwise comparisons of data from the pure breed and mixed breed dogs. Observations made between the two techniques were generally concordant, both revealing 18 breeds with distinctive phenotypes. However, in common with our work on hematological phenotypes PCA was ultimately more sensitive, yielding an additional 18 unique breeds that were not identified by pairwise comparisons; in contrast, pairwise comparisons revealed only five breeds that were not identified by PCA. While the sensitivity of PCA to detect unique breeds would clearly have been determined in part on the principal component thresholds that were employed to define ‘uniqueness’, the inferred multidimensionality of this analytical technique, which incorporates data from the whole profile rather than individual analytes, was thought to account for its greater ability to distinguish outlying breeds.

Notwithstanding the statistical nuances of these two approaches, several interesting observations were made. Analytes for which there were multiple inter-breed differences included creatinine, lipase, amylase, phosphorus and ALT. Serum creatinine concentration is known to vary with muscle mass [37, 46, 47], predicated in large part on body size, and is the basis for important diagnostic inferences and therapeutic decisions in the assessment of renal function. In our opinion, the current work emphasizes the need for breed-specific reference intervals for serum creatinine, normalized for sex and neutering status, to refine the diagnostic value of this analyte in canine medicine, as also suggested by a recent study of different sighthound breeds [48]; moreover, discrepant serum creatinine reference intervals between laboratories seem to reflect different reference populations rather than analytical variation [49]. Canine breed-specific differences in lipase, amylase and ALT activity presumably reflect global differences in the synthesis, catabolism and excretion of these enzymes, which we speculate are determined in part by genetic ‘set-points’. According with our data, recent work has suggested that serum amylase activity in Bernese mountain dogs [12] and ALT activity in Italian greyhounds differ from generic reference intervals [48], but the possibility of subclinical disease influencing these analytes could not be ruled out, with a similar caveat applying in our study. Moreover, the differences in serum ALT activity observed between breeds, while significant, were small and unlikely to impact clinical decision-making, and the diagnostic importance of serum amylase and lipase activity in dogs is questionable: the argument for breed-specific reference intervals for these analytes is therefore less compelling. We speculated that breed-specific differences in serum phosphorus concentration were also referable to genetically determined differences in its absorption, mobilization, deposition and/or excretion, perhaps predicated on varying concentrations of—or receptor sensitivity to—hormones important in its regulation, consistent with genetic loci impacting serum phosphorus concentration in humans [50]. A recent study revealed a departure from generic reference intervals for serum phosphorus in various sighthound breeds [48], again speaking to a breed-specific biochemical phenotype. Nevertheless, serum electrolyte concentrations tend to be maintained within tight reference intervals within a species owing to their critical physiological importance and factors such as dietary phosphorus intake, transcellular shifts with respiratory alkalosis, subclinical renal disease [51], or even diurnal variations [52], which could all differ between breeds, remained credible alternative explanations for this observation.

A number of breeds showed distinctive serum biochemical phenotypes on the basis of both PCA and pairwise comparisons with the control population, but in many cases the departure of values from those of the control population was small and of questionable clinical significance. Furthermore, relatively few sighthounds were recruited into our study, probably reflecting the known departure of serum biochemical analytes from generic reference intervals in these breeds and underlining the likelihood of under-estimation of breed-specific differences in this work. Nevertheless, several interesting, novel observations were made. Of particular note were pugs and CKCS. Pugs demonstrated notably lower serum sodium and chloride, and higher serum potassium concentrations than the mixed breed group, raising the possibility of differences in renal and / or gastrointestinal absorption and excretion of these electrolytes, perhaps reflecting differences in their mineralocorticoid regulation that are at least in part genetically determined. Of all the breeds in our study, the CKCS showed the greatest number of analytes with significant differences from the control group, reconciling with findings in our recent study of haematological phenotypes in dogs [45] and emphasizing the unique nature of this breed in both conformational and physiological traits.

In summary, this study has revealed the rich phenotypic diversity of serum biochemical analytes in the domestic dog, building on a number of smaller studies in this polymorphic species [12, 13, 53, 54]. While many of the absolute differences were small and of questionable clinical significance, some—in particular, those for serum creatinine—were notable and would warrant breed-specific reference intervals adjusted for age, sex and neutering status. While the optimal method to develop reliable reference intervals is to sample (a priori) a large number of rigorously assessed healthy dogs of varying age, sex, neutering status and breed, thus controlling for a number of pre-analytical variables [55–57], the practicalities of this approach in the real world present a formidable challenge. While data of this nature should be gathered in the fullness of time, more practicable approaches, such as indirect sampling (a posteriori) presents an alternative method. While our sampling method for breed specific reference intervals is a limitation of the study, the low number of instances in which truncation was considered suggests that most of the analytes of the presented breeds truly fell within the currently used intervals (further suggesting that generic canine intervals tend to be too wide). A strategy towards subject-based reference values or personalization of reference intervals for serum biochemical analytes has been suggested in human medicine, to account for age, gender and ethnicity [58, 59]In addition, the genetic homogeneity of canine breeds [15] offers a truly unique resource to investigate the genetic basis for homeostatic control of serum biochemical analytes in health, thus ultimately promising to disclose novel therapeutic targets in disease.

## Supporting Information

S1 Dataset Anonymized biochemistry data. (XLSX)

S1 Fig Effects of age, sex and neutering status on biochemical analytes for the Labrador Retriever (n = 327). The adjusted mean values ± 1 standard error bars for each of the 18 biochemical analytes are represented on the respective *y* axes, showing age in years on the *x* axes. Intact male and female dogs are represented by the solid black and red lines; neutered male and female dogs are represented by the interrupted black and red lines, respectively. These analyses were all undertaken on the Labrador retriever dogs only.
(TIF)

S2 Fig A comparison of the results of principal component analysis with pairwise comparative analysis. Breeds with a distinctive phenotype identified by principal component analysis are designated in blue, and breeds identified by pairwise comparisons with the mixed breed group are indicated in red. Complementarity was generally observed between the methods.
(TIF)

S3 Fig Histograms of the serum biochemical data for the Labrador Retriever (n = 327). (TIF)

S4 Fig Histograms of the serum biochemical data for the Cavalier King Charles Spaniel (n = 174). (TIF)

S5 Fig Histograms of the serum biochemical data for the German Shepherd Dog (n = 160). (TIF)

S6 Fig Histograms of the serum biochemical data for the Boxer (n = 146). (TIF)

S7 Fig Histograms of the serum biochemical data for the Golden Retriever (n = 121). (TIF)

S1 File Tables A-R, descriptive statistics for all serum biochemical data. Table S, p-values for the effects of age, sex and neutering status on serum biochemical profile. Table T, eigenvalues for the principal component analysis. Table U, pairwise comparison results between the pure breed and mixed breed dogs. Tables V-Z, tentative breed-specific reference intervals.
(DOC)

S1 Materials and Methods Detailed samples inclusion and exclusion criteria, specimen collection, analysis, and quality control and statistical methods. (DOCX)
