# Supplementary figures and images for: Serum Biochemical Phenotypes in the Domestic Dog
Source: PLoS One. 2016 Feb 26;11(2):e0149650. doi: 10.1371/journal.pone.0149650 (PMC4769346; doi:10.1371/journal.pone.0149650)

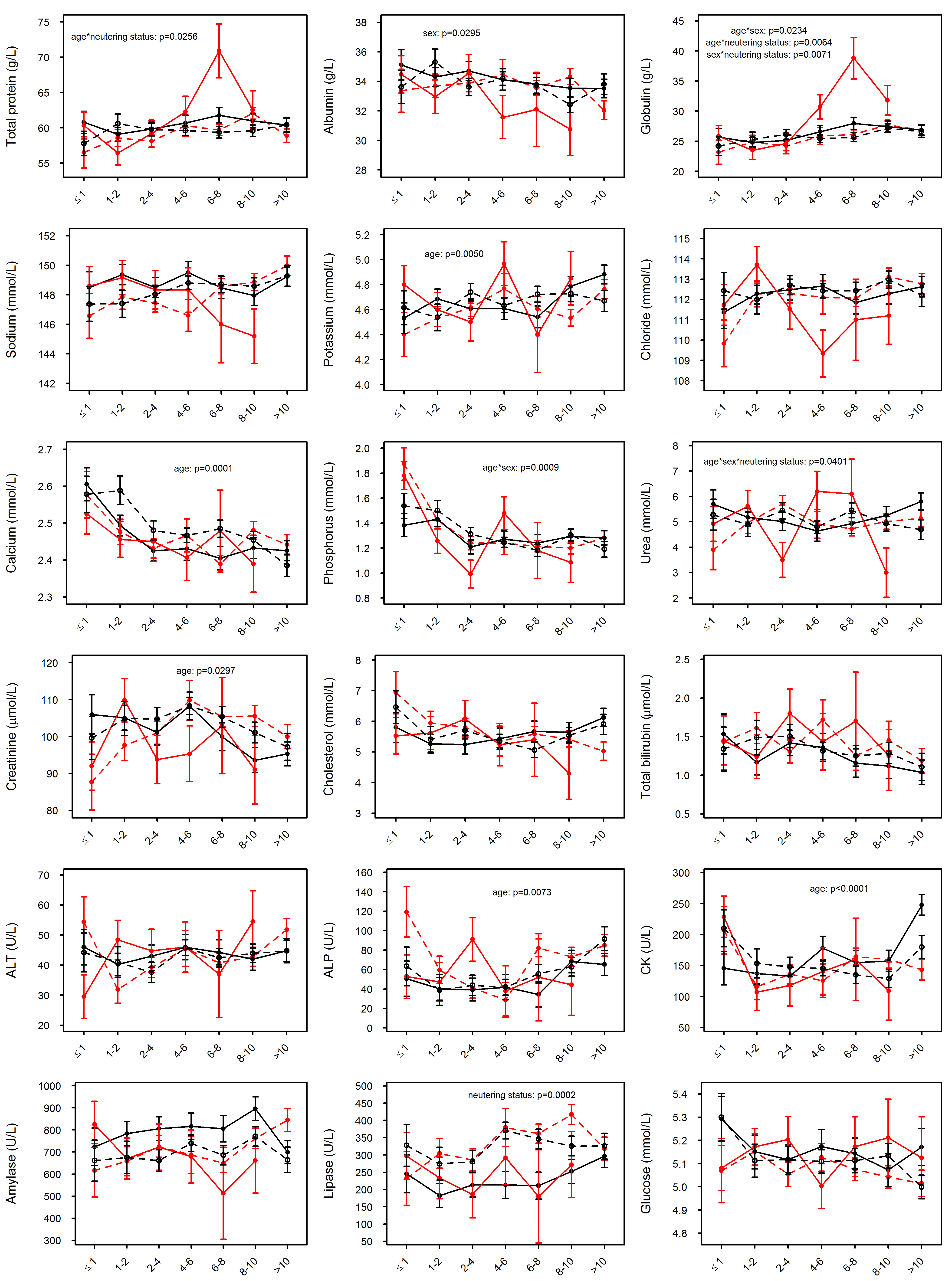

Supplement: S1 Fig — The adjusted mean values ± 1 standard error bars for each of the 18 biochemical analytes are represented on the respective y axes, showing age in years on the x axes. Intact male and female dogs are represented by the solid black and red lines; neutered male and female dogs are represented by the interrupted black and red lines, respectively. These analyses were all undertaken on the Labrador retriever dogs only. (TIF) [file pone.0149650.s002.tif]

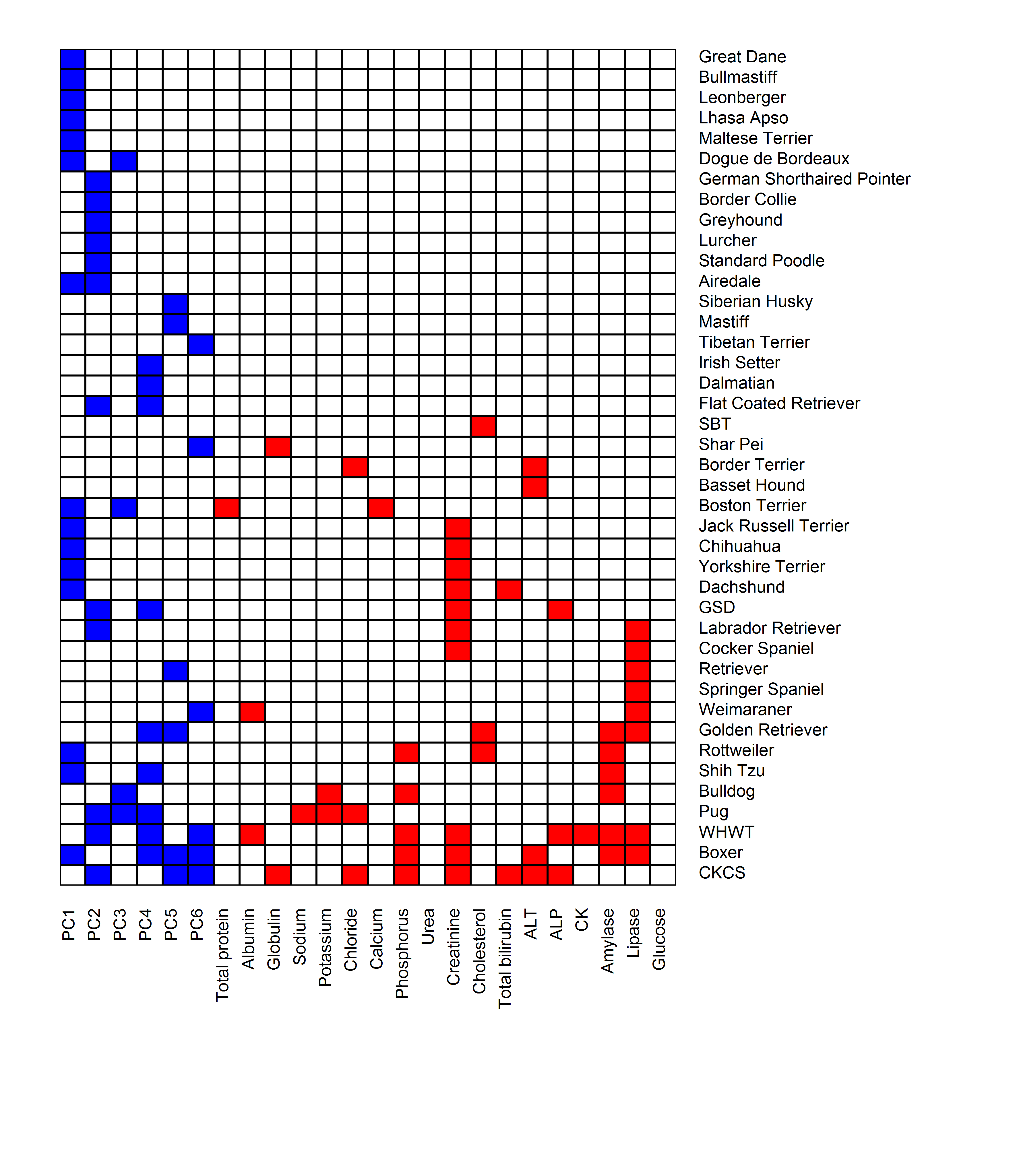

Supplement: S2 Fig — Breeds with a distinctive phenotype identified by principal component analysis are designated in blue, and breeds identified by pairwise comparisons with the mixed breed group are indicated in red. Complementarity was generally observed between the methods. (TIF) [file pone.0149650.s003.tif]

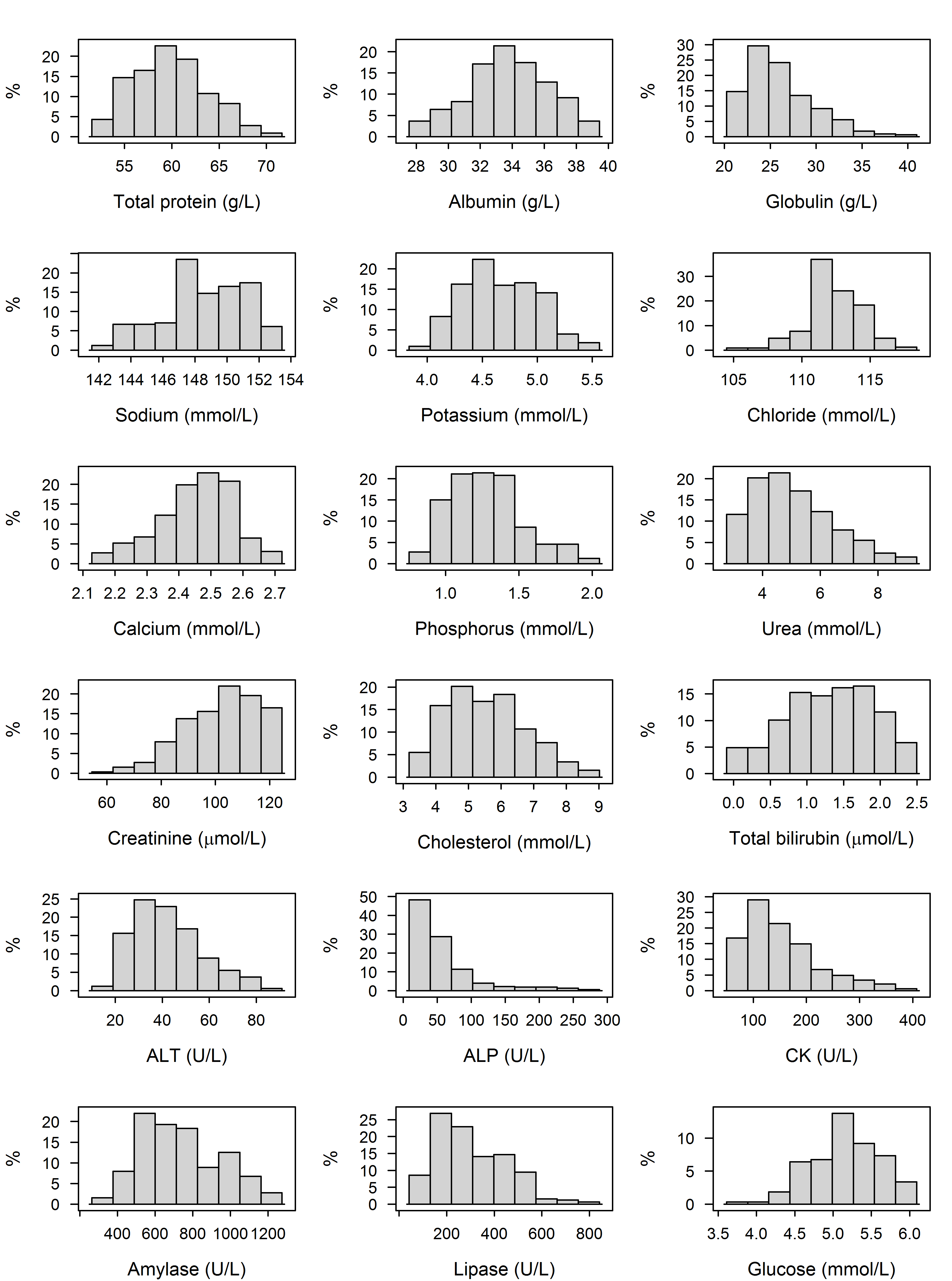

Supplement: S3 Fig — (TIF) [file pone.0149650.s004.tif]

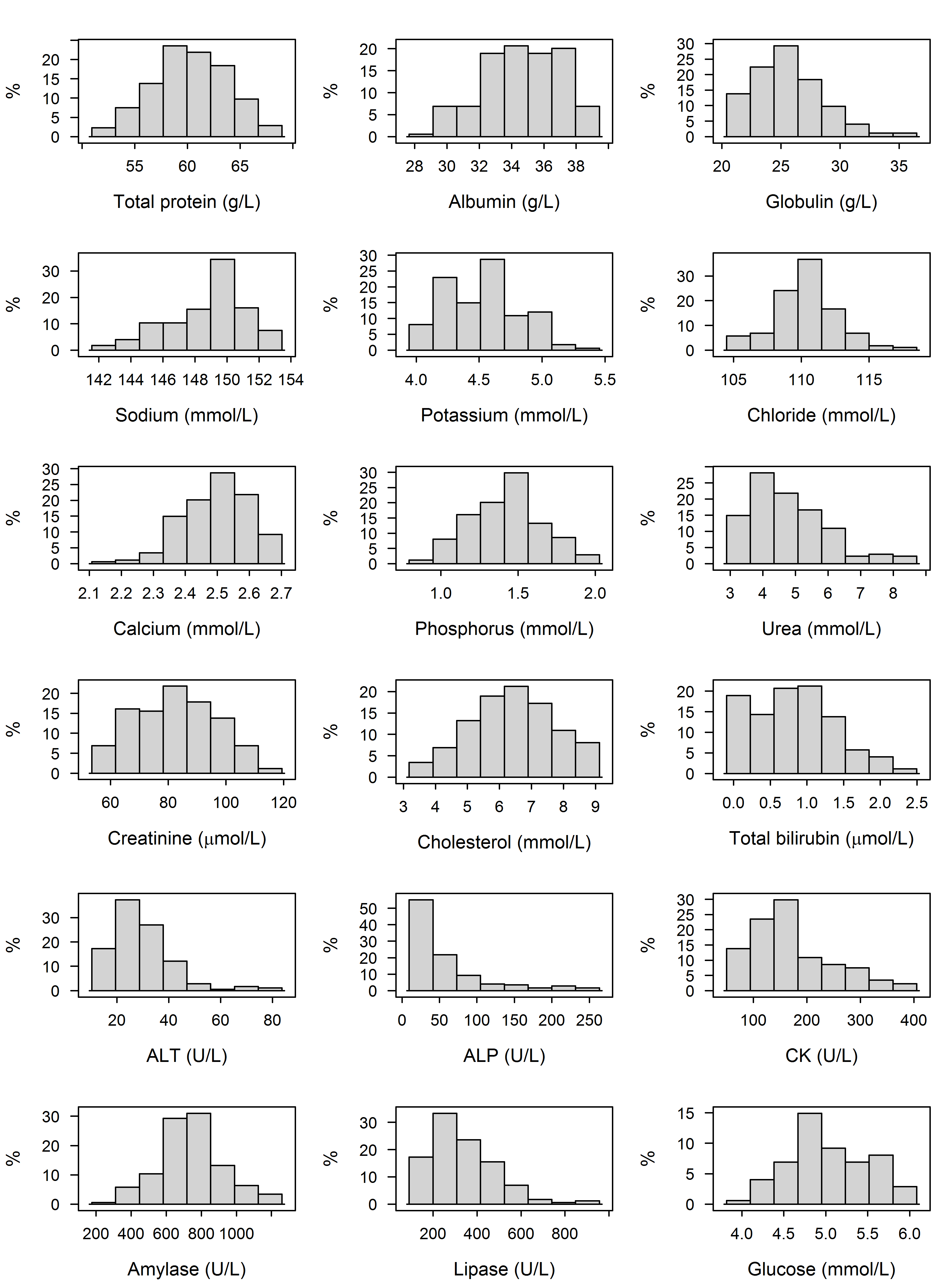

Supplement: S4 Fig — (TIF) [file pone.0149650.s005.tif]

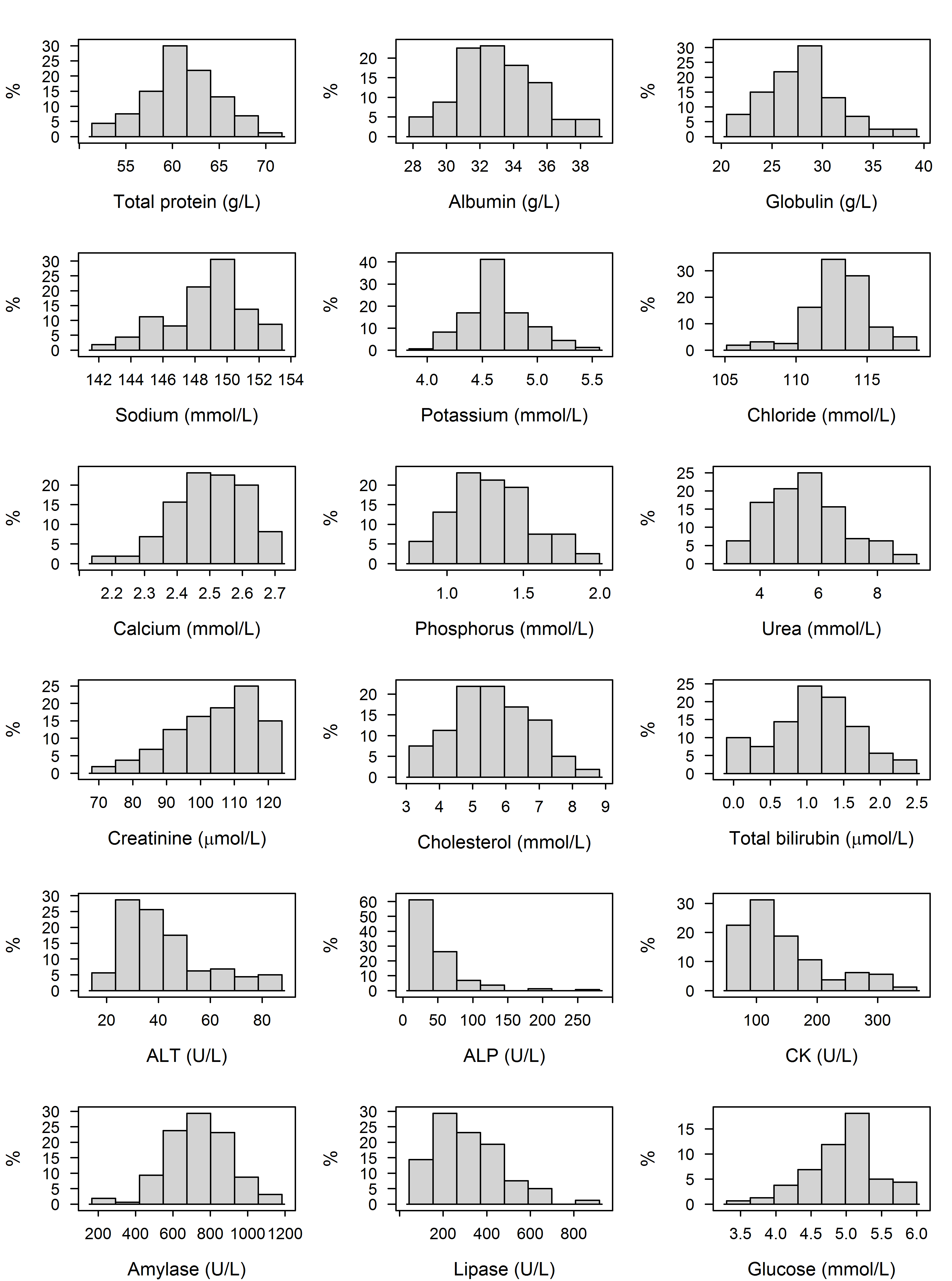

Supplement: S5 Fig — (TIF) [file pone.0149650.s006.tif]

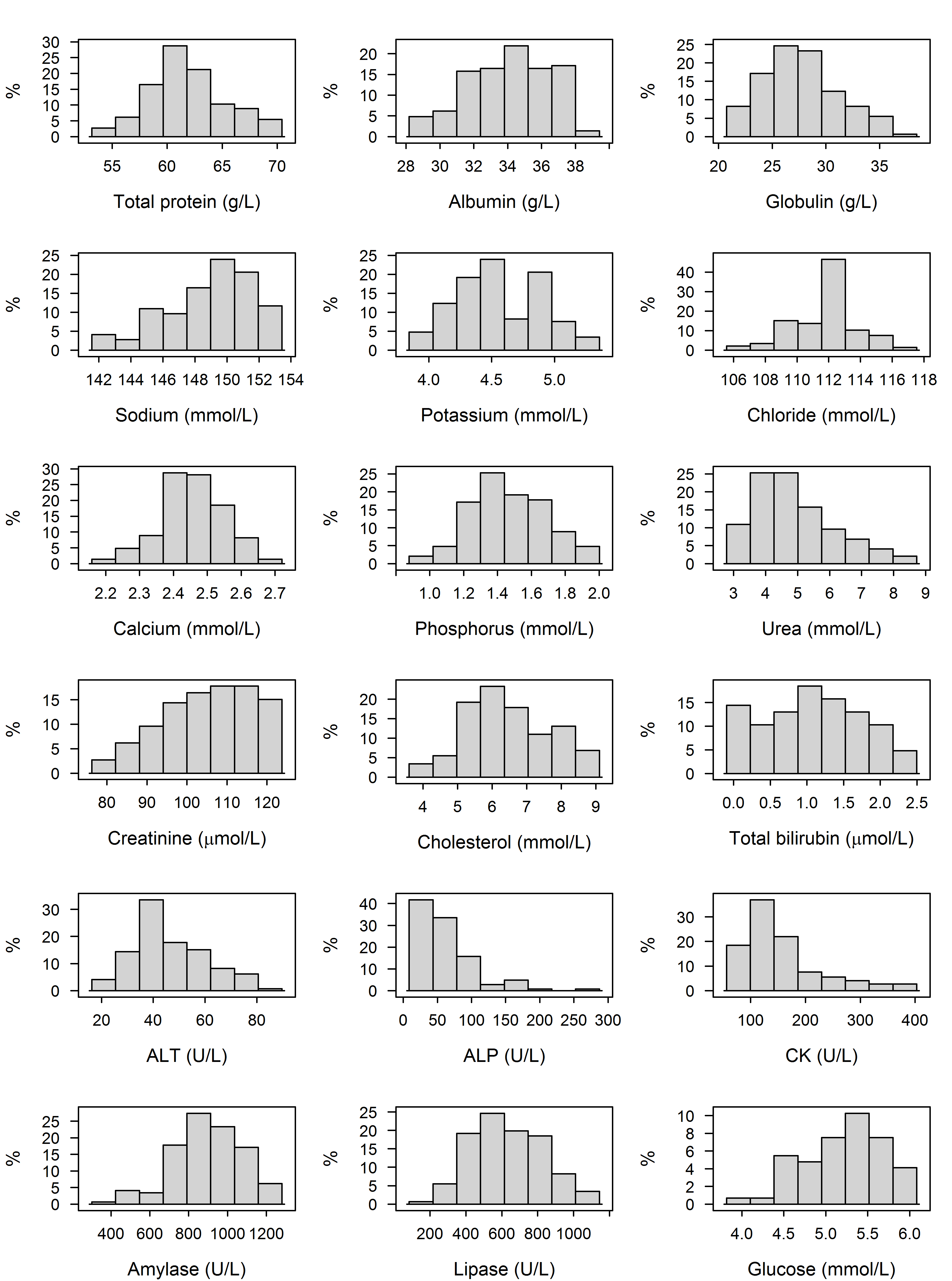

Supplement: S6 Fig — (TIF) [file pone.0149650.s007.tif]

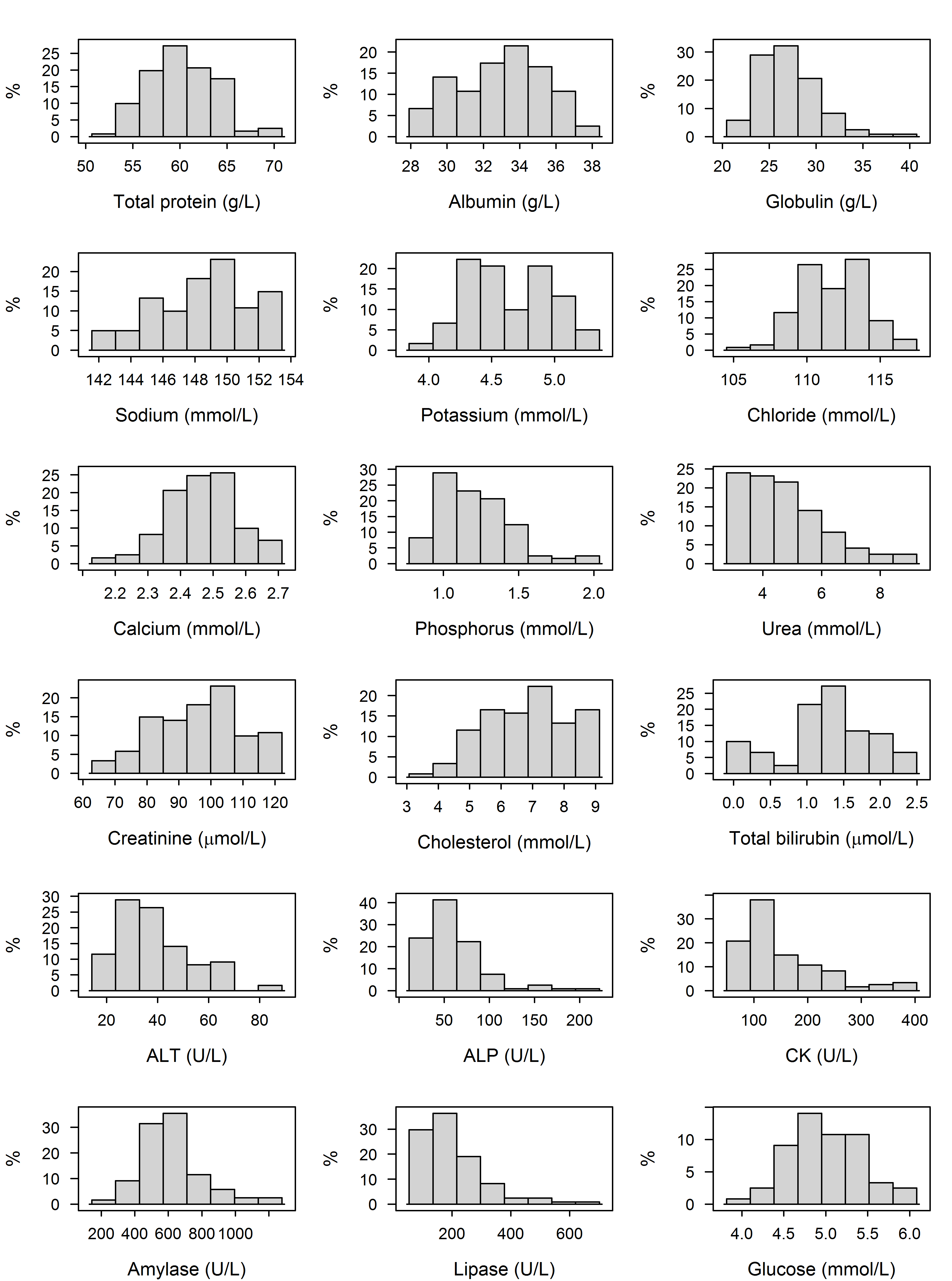

Supplement: S7 Fig — (TIF) [file pone.0149650.s008.tif]
