# Supplementary material for: Serum Biochemical Phenotypes in the Domestic Dog
Source: PLoS One. 2016 Feb 26;11(2):e0149650. doi: 10.1371/journal.pone.0149650 (PMC4769346; doi:10.1371/journal.pone.0149650)
Supplement: S1 File — Tables A-R, descriptive statistics for all serum biochemical data. Table S, p-values for the effects of age, sex and neutering status on serum biochemical profile. Table T, eigenvalues for the principal component analysis. Table U, pairwise comparison results between the pure breed and mixed breed dogs. Tables V-Z, tentative breed-specific reference intervals. (DOC) [file pone.0149650.s009.doc]

**Table A in S1 File. Descriptive statistics – total protein^§^**

| **Breed** | **N** | **Mean** | **SD** | **Median** | **IQR** | **Min.** | **Max.** |
| --- | --- | --- | --- | --- | --- | --- | --- |
| Mixed breed | 242 | 61.0 | 4.2 | 61.0 | 5.8 | 50.7 | 70.8 |
|  |  |  |  |  |  |  |  |
| **Ancient** |  |  |  |  |  |  |  |
| Maltese Terrier | 16 | 60.1 | 4.3 | 60.0 | 3.1 | 50.8 | 70.6 |
| Shar Pei | 19 | 64.5 | 3.3 | 65.7 | 5.0 | 56.6 | 68.6 |
| Siberian Husky | 12 | 61.8 | 3.7 | 61.7 | 4.3 | 56.5 | 70.0 |
| Tibetan Terrier | 17 | 59.2 | 4.2 | 59.5 | 4.7 | 51.3 | 65.9 |
|  |  |  |  |  |  |  |  |
| **Toy** |  |  |  |  |  |  |  |
| Chihuahua | 14 | 60.5 | 3.8 | 60.6 | 6.5 | 54.5 | 66.4 |
| Pug | 28 | 61.1 | 4.4 | 62.5 | 6.5 | 53.3 | 67.9 |
| Shih Tzu | 29 | 60.4 | 2.6 | 60.8 | 3.6 | 55.0 | 65.9 |
|  |  |  |  |  |  |  |  |
| **Working** |  |  |  |  |  |  |  |
| Dobermann | 77 | 60.9 | 4.0 | 61.0 | 4.4 | 49.8 | 70.3 |
| German Shepherd Dog | 160 | 61.0 | 3.8 | 60.9 | 5.1 | 52.1 | 71.0 |
| Miniature Schnauzer | 24 | 60.9 | 3.5 | 60.7 | 5.2 | 55.5 | 67.3 |
| Schnauzer | 13 | 60.1 | 3.7 | 60.2 | 4.4 | 52.6 | 66.9 |
|  |  |  |  |  |  |  |  |
| **Sight hound** |  |  |  |  |  |  |  |
| Greyhound | 14 | 57.9 | 4.1 | 58.4 | 5.2 | 50.6 | 65.6 |
| Lurcher | 19 | 59.1 | 2.9 | 59.3 | 4.0 | 54.5 | 64.5 |
|  |  |  |  |  |  |  |  |
| **Mastiff-like** |  |  |  |  |  |  |  |
| Boston Terrier | 15 | 56.8 | 2.6 | 56.3 | 3.2 | 50.8 | 61.4 |
| Boxer | 146 | 61.9 | 3.5 | 61.4 | 4.5 | 53.8 | 69.8 |
| Bullmastiff | 23 | 62.3 | 3.5 | 62.5 | 5.9 | 57.2 | 69.0 |
| Bulldog | 39 | 60.0 | 4.7 | 60.5 | 6.3 | 50.2 | 70.5 |
| Dogue de Bordeaux | 13 | 60.7 | 4.3 | 61.0 | 6.4 | 54.1 | 68.6 |
| French Bulldog | 20 | 59.9 | 4.1 | 59.6 | 3.5 | 53.6 | 68.4 |
| Mastiff | 13 | 63.1 | 3.7 | 63.7 | 4.5 | 56.3 | 69.1 |
| Staffordshire Bull Terrier | 95 | 60.9 | 4.0 | 60.6 | 4.7 | 51.7 | 70.4 |
|  |  |  |  |  |  |  |  |
| **Retriever/other Mastiff-like** |  |  |  |  |  |  |  |
| Flat Coated Retriever | 12 | 57.2 | 3.0 | 56.4 | 3.6 | 53.2 | 63.1 |
| Golden Retriever | 121 | 60.1 | 3.5 | 59.9 | 5.2 | 51.4 | 70.1 |
| Great Dane | 25 | 62.3 | 4.2 | 62.7 | 3.7 | 52.5 | 70.4 |
| Labrador Retriever | 327 | 59.9 | 3.9 | 59.6 | 5.8 | 52.3 | 70.9 |
| Leonberger | 15 | 63.0 | 3.4 | 62.9 | 4.0 | 56.4 | 68.5 |
| Newfoundland | 29 | 62.2 | 4.0 | 61.2 | 5.6 | 52.9 | 70.8 |
| Retriever | 36 | 59.7 | 4.0 | 59.0 | 5.6 | 51.1 | 69.3 |
| Rottweiler | 49 | 61.9 | 4.0 | 61.7 | 5.7 | 55.1 | 69.5 |
|  |  |  |  |  |  |  |  |
| **Herding** |  |  |  |  |  |  |  |
| Border Collie | 73 | 59.6 | 4.3 | 59.1 | 5.4 | 50.8 | 70.1 |
| Collie | 14 | 59.6 | 4.6 | 58.3 | 8.6 | 53.8 | 66.5 |
| Shetland Sheepdog | 11 | 62.2 | 3.7 | 62.0 | 5.3 | 56.0 | 68.0 |
|  |  |  |  |  |  |  |  |
| **Terrier** |  |  |  |  |  |  |  |
| Airedale | 10 | 64.8 | 3.9 | 65.8 | 4.4 | 57.7 | 69.8 |
| Border Terrier | 35 | 61.3 | 4.6 | 61.0 | 6.1 | 52.4 | 69.7 |
| Norfolk Terrier | 12 | 60.4 | 3.3 | 60.1 | 4.7 | 55.5 | 66.1 |
| West Highland White Terrier | 78 | 62.4 | 4.0 | 62.5 | 6.1 | 54.1 | 69.4 |
| Yorkshire Terrier | 41 | 61.0 | 3.8 | 60.6 | 5.3 | 53.3 | 67.7 |
|  |  |  |  |  |  |  |  |
| **Scent hound** |  |  |  |  |  |  |  |
| Basset Hound | 21 | 61.2 | 3.6 | 59.6 | 6.1 | 56.5 | 69.7 |
| Beagle | 29 | 60.3 | 4.1 | 60.3 | 5.2 | 49.8 | 68.3 |
| Dachshund | 34 | 60.8 | 4.1 | 60.3 | 4.6 | 53.4 | 71.0 |
| Rhodesian Ridgeback | 32 | 61.7 | 3.9 | 62.2 | 5.2 | 51.8 | 68.4 |
|  |  |  |  |  |  |  |  |
| **Spaniel/Pointer** |  |  |  |  |  |  |  |
| Cavalier King Charles Spaniel | 174 | 60.3 | 3.6 | 60.4 | 4.9 | 51.6 | 68.3 |
| Cocker Spaniel | 98 | 61.5 | 3.6 | 61.3 | 3.7 | 52.6 | 70.5 |
| English Springer Spaniel | 41 | 60.3 | 3.6 | 59.9 | 4.4 | 54.2 | 69.3 |
| German Shorthaired Pointer | 19 | 58.4 | 3.6 | 59.1 | 3.1 | 50.6 | 66.6 |
| Gordon Setter | 14 | 62.4 | 3.9 | 64.0 | 3.2 | 54.3 | 67.7 |
| Hungarian Vizsla | 15 | 59.5 | 3.7 | 59.4 | 3.0 | 54.1 | 69.5 |
| Irish Setter | 23 | 61.1 | 4.3 | 60.5 | 5.4 | 54.1 | 70.5 |
| Italian Spinone | 21 | 61.4 | 4.9 | 60.2 | 6.4 | 53.2 | 70.4 |
| Pointer | 13 | 60.3 | 4.5 | 57.4 | 6.2 | 55.7 | 69.1 |
| Springer Spaniel | 71 | 61.9 | 4.1 | 61.7 | 5.1 | 51.9 | 70.4 |
| Weimaraner | 39 | 60.3 | 4.9 | 59.3 | 6.2 | 50.4 | 70.6 |
|  |  |  |  |  |  |  |  |
| **Other** |  |  |  |  |  |  |  |
| Bichon Frise | 14 | 60.9 | 3.8 | 61.5 | 3.4 | 53.5 | 67.1 |
| Collie cross | 16 | 60.6 | 5.1 | 60.9 | 5.2 | 53.7 | 70.7 |
| Dalmatian | 21 | 59.6 | 3.6 | 59.1 | 4.2 | 54.2 | 67.1 |
| Jack Russell Terrier | 78 | 60.8 | 3.7 | 60.5 | 4.9 | 54.7 | 70.2 |
| Labrador cross | 15 | 60.5 | 3.2 | 61.1 | 4.0 | 54.9 | 65.6 |
| Lhasa Apso | 17 | 57.4 | 2.5 | 57.3 | 2.9 | 52.2 | 61.2 |
| Poodle | 18 | 59.1 | 2.6 | 59.7 | 3.5 | 54.6 | 63.0 |
| Standard Poodle | 11 | 58.0 | 4.9 | 57.4 | 6.4 | 52.0 | 66.7 |

§ Unit of measurement: g/L; SD = standard deviation; IQR = interquartile range; Min. = minimum value recorded; Max. = maximum value recorded.

**Table B in S1 File. Descriptive statistics – albumin^§^**

| **Breed** | **N** | **Mean** | **SD** | **Median** | **IQR** | **Min.** | **Max.** |
| --- | --- | --- | --- | --- | --- | --- | --- |
| Mixed breed | 242 | 33.8 | 2.6 | 33.9 | 3.6 | 28.4 | 39.0 |
|  |  |  |  |  |  |  |  |
| **Ancient** |  |  |  |  |  |  |  |
| Maltese Terrier | 16 | 34.6 | 2.6 | 34.6 | 3.2 | 28.4 | 38.1 |
| Shar Pei | 19 | 32.8 | 1.9 | 33.3 | 2.5 | 29.8 | 37.5 |
| Siberian Husky | 12 | 35.6 | 1.8 | 35.7 | 1.5 | 32.3 | 38.6 |
| Tibetan Terrier | 17 | 32.7 | 2.3 | 32.3 | 2.0 | 29.1 | 37.3 |
|  |  |  |  |  |  |  |  |
| **Toy** |  |  |  |  |  |  |  |
| Chihuahua | 14 | 35.5 | 2.4 | 36.1 | 3.7 | 30.8 | 38.6 |
| Pug | 28 | 33.3 | 2.0 | 33.4 | 2.3 | 29.2 | 37.6 |
| Shih Tzu | 29 | 35.0 | 1.9 | 35.1 | 2.4 | 31.2 | 38.7 |
|  |  |  |  |  |  |  |  |
| **Working** |  |  |  |  |  |  |  |
| Dobermann | 77 | 33.5 | 2.5 | 33.4 | 3.3 | 28.5 | 38.7 |
| German Shepherd Dog | 160 | 33.1 | 2.4 | 33.0 | 3.3 | 28.2 | 38.7 |
| Miniature Schnauzer | 24 | 34.7 | 2.4 | 34.4 | 2.7 | 30.4 | 39.0 |
| Schnauzer | 13 | 33.9 | 2.3 | 34.6 | 4.0 | 30.7 | 37.4 |
|  |  |  |  |  |  |  |  |
| **Sight hound** |  |  |  |  |  |  |  |
| Greyhound | 14 | 33.5 | 3.0 | 33.2 | 4.4 | 28.4 | 37.5 |
| Lurcher | 19 | 34.5 | 2.2 | 34.6 | 2.5 | 30.8 | 38.6 |
|  |  |  |  |  |  |  |  |
| **Mastiff-like** |  |  |  |  |  |  |  |
| Boston Terrier | 15 | 32.7 | 2.5 | 32.4 | 1.9 | 29.5 | 38.7 |
| Boxer | 146 | 34.1 | 2.5 | 34.2 | 3.8 | 28.6 | 39.0 |
| Bullmastiff | 23 | 33.4 | 2.5 | 33.9 | 4.5 | 28.3 | 36.5 |
| Bulldog | 39 | 33.1 | 2.6 | 33.4 | 3.4 | 28.0 | 38.7 |
| Dogue de Bordeaux | 13 | 32.5 | 2.2 | 32.5 | 3.0 | 29.0 | 35.3 |
| French Bulldog | 20 | 34.5 | 2.6 | 35.0 | 2.6 | 29.2 | 38.8 |
| Mastiff | 13 | 34.5 | 1.8 | 35.0 | 2.8 | 30.7 | 36.8 |
| Staffordshire Bull Terrier | 95 | 34.5 | 2.6 | 35.0 | 3.8 | 28.4 | 38.6 |
|  |  |  |  |  |  |  |  |
| **Retriever/other Mastiff-like** |  |  |  |  |  |  |  |
| Flat Coated Retriever | 12 | 32.1 | 1.9 | 32.3 | 3.4 | 29.2 | 34.8 |
| Golden Retriever | 121 | 33.0 | 2.4 | 33.3 | 3.6 | 28.3 | 38.0 |
| Great Dane | 25 | 33.3 | 2.7 | 33.6 | 4.0 | 29.8 | 38.3 |
| Labrador Retriever | 327 | 33.7 | 2.5 | 33.6 | 3.4 | 28.0 | 39.0 |
| Leonberger | 15 | 33.9 | 3.2 | 34.7 | 5.8 | 28.7 | 37.9 |
| Newfoundland | 29 | 34.3 | 2.7 | 34.2 | 3.7 | 29.0 | 38.8 |
| Retriever | 36 | 32.4 | 2.2 | 32.5 | 2.8 | 28.2 | 37.3 |
| Rottweiler | 49 | 32.9 | 2.5 | 33.0 | 3.3 | 28.0 | 38.4 |
|  |  |  |  |  |  |  |  |
| **Herding** |  |  |  |  |  |  |  |
| Border Collie | 73 | 33.5 | 2.5 | 33.9 | 4.0 | 28.0 | 39.0 |
| Collie | 14 | 32.2 | 2.2 | 32.6 | 1.9 | 28.6 | 36.0 |
| Shetland Sheepdog | 11 | 35.0 | 2.7 | 34.8 | 3.6 | 30.6 | 38.9 |
|  |  |  |  |  |  |  |  |
| **Terrier** |  |  |  |  |  |  |  |
| Airedale | 10 | 35.2 | 2.2 | 35.2 | 3.1 | 31.8 | 38.7 |
| Border Terrier | 35 | 34.8 | 2.7 | 35.2 | 4.6 | 30.1 | 38.8 |
| Norfolk Terrier | 12 | 34.1 | 2.5 | 34.5 | 3.9 | 30.3 | 37.8 |
| West Highland White Terrier | 78 | 35.5 | 2.5 | 35.8 | 3.6 | 29.2 | 38.9 |
| Yorkshire Terrier | 41 | 34.8 | 2.4 | 35.4 | 3.5 | 29.7 | 38.5 |
|  |  |  |  |  |  |  |  |
| **Scent hound** |  |  |  |  |  |  |  |
| Basset Hound | 21 | 34.1 | 2.7 | 34.5 | 2.3 | 28.6 | 38.2 |
| Beagle | 29 | 33.3 | 3.2 | 34.4 | 4.2 | 28.2 | 38.2 |
| Dachshund | 34 | 34.5 | 2.6 | 34.6 | 4.5 | 29.1 | 38.3 |
| Rhodesian Ridgeback | 32 | 33.8 | 3.2 | 33.9 | 4.9 | 28.0 | 39.0 |
|  |  |  |  |  |  |  |  |
| **Spaniel/Pointer** |  |  |  |  |  |  |  |
| Cavalier King Charles Spaniel | 174 | 34.7 | 2.4 | 34.8 | 3.5 | 28.1 | 39.0 |
| Cocker Spaniel | 98 | 33.9 | 2.4 | 33.9 | 3.7 | 28.3 | 38.6 |
| English Springer Spaniel | 41 | 33.6 | 2.0 | 33.1 | 2.1 | 29.7 | 38.2 |
| German Shorthaired Pointer | 19 | 33.0 | 2.3 | 32.9 | 2.7 | 28.9 | 36.4 |
| Gordon Setter | 14 | 34.4 | 2.4 | 35.0 | 2.6 | 28.6 | 37.0 |
| Hungarian Vizsla | 15 | 33.5 | 2.0 | 33.6 | 2.8 | 31.1 | 38.1 |
| Irish Setter | 23 | 33.6 | 1.7 | 33.6 | 2.8 | 31.1 | 36.3 |
| Italian Spinone | 21 | 34.2 | 2.7 | 33.9 | 4.4 | 28.7 | 38.4 |
| Pointer | 13 | 32.2 | 2.5 | 32.8 | 2.4 | 28.2 | 37.2 |
| Springer Spaniel | 71 | 33.7 | 2.4 | 33.3 | 3.7 | 28.8 | 38.9 |
| Weimaraner | 39 | 31.5 | 2.3 | 30.7 | 3.5 | 28.1 | 36.2 |
|  |  |  |  |  |  |  |  |
| **Other** |  |  |  |  |  |  |  |
| Bichon Frise | 14 | 34.6 | 3.2 | 34.5 | 5.8 | 29.4 | 38.3 |
| Collie cross | 16 | 32.5 | 2.6 | 32.7 | 3.7 | 28.8 | 38.1 |
| Dalmatian | 21 | 34.1 | 2.0 | 34.1 | 2.8 | 30.8 | 37.2 |
| Jack Russell Terrier | 78 | 34.9 | 2.4 | 35.2 | 2.8 | 28.4 | 38.8 |
| Labrador cross | 15 | 34.3 | 2.4 | 34.3 | 3.0 | 29.9 | 38.0 |
| Lhasa Apso | 17 | 33.1 | 2.5 | 33.4 | 3.4 | 28.3 | 36.3 |
| Poodle | 18 | 33.8 | 2.3 | 34.3 | 2.7 | 29.7 | 39.0 |
| Standard Poodle | 11 | 33.2 | 2.5 | 32.9 | 3.9 | 29.5 | 37.2 |

§ Unit of measurement: g/L; SD = standard deviation; IQR = interquartile range; Min. = minimum value recorded; Max. = maximum value recorded.

**Table C in S1 File. Descriptive statistics – globulin^§^**

| **Breed** | **N** | **Mean** | **SD** | **Median** | **IQR** | **Min.** | **Max.** |
| --- | --- | --- | --- | --- | --- | --- | --- |
| Mixed breed | 242 | 27.2 | 3.6 | 26.9 | 5.4 | 21.0 | 39.3 |
|  |  |  |  |  |  |  |  |
| **Ancient** |  |  |  |  |  |  |  |
| Maltese Terrier | 16 | 25.4 | 3.1 | 24.5 | 3.4 | 22.4 | 33.5 |
| Shar Pei | 19 | 31.7 | 3.6 | 32.1 | 4.8 | 21.7 | 36.4 |
| Siberian Husky | 12 | 26.2 | 3.8 | 25.4 | 2.9 | 22.6 | 36.6 |
| Tibetan Terrier | 17 | 26.4 | 3.4 | 25.3 | 4.0 | 21.3 | 33.9 |
|  |  |  |  |  |  |  |  |
| **Toy** |  |  |  |  |  |  |  |
| Chihuahua | 14 | 25.0 | 2.7 | 24.9 | 4.3 | 21.0 | 30.0 |
| Pug | 28 | 27.9 | 3.8 | 28.2 | 4.8 | 21.2 | 34.4 |
| Shih Tzu | 29 | 25.4 | 2.6 | 25.2 | 3.4 | 21.3 | 31.2 |
|  |  |  |  |  |  |  |  |
| **Working** |  |  |  |  |  |  |  |
| Dobermann | 77 | 27.4 | 3.6 | 26.8 | 4.7 | 21.1 | 35.5 |
| German Shepherd Dog | 160 | 28.0 | 3.6 | 27.8 | 4.2 | 21.2 | 38.6 |
| Miniature Schnauzer | 24 | 26.2 | 4.0 | 25.2 | 5.9 | 21.0 | 34.9 |
| Schnauzer | 13 | 26.2 | 2.4 | 26.0 | 2.3 | 21.7 | 29.5 |
|  |  |  |  |  |  |  |  |
| **Sight hound** |  |  |  |  |  |  |  |
| Greyhound | 14 | 24.5 | 2.4 | 23.6 | 2.8 | 21.4 | 29.8 |
| Lurcher | 19 | 24.6 | 2.2 | 24.2 | 2.7 | 22.0 | 28.9 |
|  |  |  |  |  |  |  |  |
| **Mastiff-like** |  |  |  |  |  |  |  |
| Boston Terrier | 15 | 24.2 | 2.0 | 23.6 | 2.7 | 21.3 | 28.0 |
| Boxer | 146 | 27.8 | 3.6 | 27.4 | 4.9 | 21.4 | 37.8 |
| Bullmastiff | 23 | 28.9 | 2.7 | 28.8 | 3.5 | 24.1 | 35.4 |
| Bulldog | 39 | 26.9 | 3.7 | 26.8 | 4.9 | 21.0 | 34.2 |
| Dogue de Bordeaux | 13 | 28.2 | 3.3 | 29.2 | 4.4 | 22.7 | 34.2 |
| French Bulldog | 20 | 25.4 | 3.1 | 24.6 | 2.9 | 21.1 | 33.0 |
| Mastiff | 13 | 28.6 | 2.7 | 29.5 | 4.3 | 24.6 | 32.9 |
| Staffordshire Bull Terrier | 95 | 26.4 | 3.3 | 25.8 | 4.8 | 21.4 | 35.9 |
|  |  |  |  |  |  |  |  |
| **Retriever/other Mastiff-like** |  |  |  |  |  |  |  |
| Flat Coated Retriever | 12 | 25.1 | 2.6 | 24.8 | 1.7 | 21.0 | 29.9 |
| Golden Retriever | 121 | 27.0 | 3.2 | 26.7 | 3.9 | 21.2 | 40.0 |
| Great Dane | 25 | 29.0 | 3.1 | 29.5 | 4.6 | 22.5 | 35.8 |
| Labrador Retriever | 327 | 26.2 | 3.6 | 25.7 | 4.9 | 21.0 | 40.2 |
| Leonberger | 15 | 29.2 | 5.1 | 28.4 | 5.6 | 21.5 | 39.4 |
| Newfoundland | 29 | 28.0 | 3.6 | 27.1 | 3.4 | 23.3 | 39.8 |
| Retriever | 36 | 27.3 | 3.1 | 27.1 | 4.7 | 22.1 | 33.9 |
| Rottweiler | 49 | 29.0 | 3.9 | 28.0 | 5.8 | 21.3 | 38.8 |
|  |  |  |  |  |  |  |  |
| **Herding** |  |  |  |  |  |  |  |
| Border Collie | 73 | 26.1 | 3.8 | 25.8 | 5.0 | 21.1 | 38.6 |
| Collie | 14 | 27.4 | 5.0 | 26.0 | 9.1 | 21.9 | 35.7 |
| Shetland Sheepdog | 11 | 27.2 | 2.5 | 27.5 | 2.5 | 21.1 | 30.3 |
|  |  |  |  |  |  |  |  |
| **Terrier** |  |  |  |  |  |  |  |
| Airedale | 10 | 29.6 | 3.7 | 30.5 | 3.4 | 22.8 | 35.1 |
| Border Terrier | 35 | 26.5 | 3.7 | 26.2 | 6.0 | 21.4 | 34.8 |
| Norfolk Terrier | 12 | 26.2 | 3.8 | 26.1 | 2.6 | 21.5 | 35.8 |
| West Highland White Terrier | 78 | 26.9 | 3.8 | 27.3 | 4.9 | 21.0 | 40.1 |
| Yorkshire Terrier | 41 | 26.1 | 3.3 | 25.5 | 5.2 | 21.4 | 33.4 |
|  |  |  |  |  |  |  |  |
| **Scent hound** |  |  |  |  |  |  |  |
| Basset Hound | 21 | 27.0 | 2.4 | 27.7 | 2.1 | 21.8 | 31.8 |
| Beagle | 29 | 26.9 | 4.6 | 26.3 | 5.0 | 21.2 | 40.0 |
| Dachshund | 34 | 26.3 | 3.9 | 25.6 | 5.0 | 21.8 | 36.1 |
| Rhodesian Ridgeback | 32 | 27.9 | 3.8 | 26.9 | 4.7 | 22.8 | 38.7 |
|  |  |  |  |  |  |  |  |
| **Spaniel/Pointer** |  |  |  |  |  |  |  |
| Cavalier King Charles Spaniel | 174 | 25.7 | 2.9 | 25.5 | 3.9 | 21.0 | 35.9 |
| Cocker Spaniel | 98 | 27.6 | 3.4 | 27.1 | 4.5 | 21.8 | 36.7 |
| English Springer Spaniel | 41 | 26.7 | 3.4 | 27.0 | 4.8 | 21.4 | 35.8 |
| German Shorthaired Pointer | 19 | 25.4 | 2.4 | 24.9 | 3.0 | 21.4 | 30.4 |
| Gordon Setter | 14 | 28.0 | 3.2 | 28.4 | 3.1 | 23.0 | 35.7 |
| Hungarian Vizsla | 15 | 26.0 | 2.3 | 25.4 | 3.0 | 23.0 | 31.4 |
| Irish Setter | 23 | 27.5 | 4.2 | 26.9 | 4.1 | 21.5 | 38.2 |
| Italian Spinone | 21 | 27.2 | 3.8 | 26.0 | 4.7 | 22.6 | 35.5 |
| Pointer | 13 | 28.1 | 4.7 | 27.5 | 4.7 | 22.7 | 40.3 |
| Springer Spaniel | 71 | 28.2 | 4.0 | 27.4 | 6.6 | 21.2 | 37.0 |
| Weimaraner | 39 | 28.8 | 5.3 | 28.1 | 8.0 | 21.2 | 39.6 |
|  |  |  |  |  |  |  |  |
| **Other** |  |  |  |  |  |  |  |
| Bichon Frise | 14 | 26.3 | 4.3 | 24.4 | 6.2 | 21.6 | 34.4 |
| Collie cross | 16 | 28.1 | 3.9 | 27.9 | 4.8 | 21.7 | 36.3 |
| Dalmatian | 21 | 25.6 | 3.4 | 23.9 | 6.3 | 21.7 | 32.1 |
| Jack Russell Terrier | 78 | 25.9 | 3.4 | 25.2 | 4.2 | 21.2 | 35.2 |
| Labrador cross | 15 | 26.2 | 2.2 | 25.9 | 3.1 | 22.1 | 29.8 |
| Lhasa Apso | 17 | 24.4 | 2.3 | 23.9 | 3.8 | 21.2 | 28.9 |
| Poodle | 18 | 25.2 | 1.8 | 25.7 | 2.4 | 21.3 | 28.8 |
| Standard Poodle | 11 | 24.8 | 3.0 | 24.3 | 4.6 | 21.0 | 30.1 |

§ Unit of measurement: g/L; SD = standard deviation; IQR = interquartile range; Min. = minimum value recorded; Max. = maximum value recorded.

**Table D in S1 File. Descriptive statistics – sodium^§^**

| **Breed** | **N** | **Mean** | **SD** | **Median** | **IQR** | **Min.** | **Max.** |
| --- | --- | --- | --- | --- | --- | --- | --- |
| Mixed breed | 242 | 148.5 | 2.5 | 149.0 | 3.4 | 142.0 | 153.0 |
|  |  |  |  |  |  |  |  |
| **Ancient** |  |  |  |  |  |  |  |
| Maltese Terrier | 16 | 146.5 | 3.0 | 146.0 | 5.0 | 143.0 | 151.0 |
| Shar Pei | 19 | 148.2 | 2.4 | 148.0 | 3.0 | 143.0 | 152.0 |
| Siberian Husky | 12 | 148.6 | 2.8 | 149.0 | 4.2 | 144.0 | 152.9 |
| Tibetan Terrier | 17 | 149.1 | 2.1 | 149.0 | 3.0 | 145.0 | 152.0 |
|  |  |  |  |  |  |  |  |
| **Toy** |  |  |  |  |  |  |  |
| Chihuahua | 14 | 147.4 | 3.0 | 147.5 | 3.5 | 142.0 | 151.1 |
| Pug | 28 | 145.8 | 1.9 | 145.1 | 2.0 | 142.0 | 149.0 |
| Shih Tzu | 29 | 147.5 | 2.3 | 148.0 | 4.6 | 143.5 | 150.8 |
|  |  |  |  |  |  |  |  |
| **Working** |  |  |  |  |  |  |  |
| Dobermann | 77 | 148.4 | 2.4 | 148.0 | 3.6 | 143.0 | 153.0 |
| German Shepherd Dog | 160 | 148.6 | 2.4 | 149.0 | 2.7 | 142.0 | 153.0 |
| Miniature Schnauzer | 24 | 147.7 | 2.1 | 147.4 | 2.7 | 143.0 | 152.2 |
| Schnauzer | 13 | 149.4 | 2.1 | 149.7 | 3.0 | 145.1 | 153.0 |
|  |  |  |  |  |  |  |  |
| **Sight hound** |  |  |  |  |  |  |  |
| Greyhound | 14 | 148.5 | 2.6 | 148.4 | 5.0 | 144.8 | 152.9 |
| Lurcher | 19 | 149.2 | 2.2 | 149.4 | 3.0 | 145.0 | 153.0 |
|  |  |  |  |  |  |  |  |
| **Mastiff-like** |  |  |  |  |  |  |  |
| Boston Terrier | 15 | 148.1 | 3.0 | 148.2 | 2.4 | 143.0 | 153.0 |
| Boxer | 146 | 148.9 | 2.6 | 149.0 | 4.0 | 142.0 | 153.0 |
| Bullmastiff | 23 | 147.7 | 2.9 | 148.0 | 3.5 | 142.6 | 153.0 |
| Bulldog | 39 | 147.9 | 2.5 | 148.4 | 3.4 | 142.0 | 151.0 |
| Dogue de Bordeaux | 13 | 146.0 | 2.1 | 146.2 | 3.5 | 143.0 | 150.0 |
| French Bulldog | 20 | 148.2 | 2.4 | 148.6 | 3.8 | 144.0 | 152.3 |
| Mastiff | 13 | 147.7 | 2.4 | 147.4 | 3.6 | 145.0 | 152.0 |
| Staffordshire Bull Terrier | 95 | 148.0 | 2.3 | 148.0 | 2.4 | 142.6 | 153.0 |
|  |  |  |  |  |  |  |  |
| **Retriever/other Mastiff-like** |  |  |  |  |  |  |  |
| Flat Coated Retriever | 12 | 149.0 | 3.1 | 148.0 | 5.3 | 144.9 | 152.9 |
| Golden Retriever | 121 | 148.5 | 2.7 | 148.8 | 3.7 | 142.0 | 153.0 |
| Great Dane | 25 | 148.5 | 2.7 | 148.4 | 4.8 | 143.0 | 153.0 |
| Labrador Retriever | 327 | 148.6 | 2.6 | 148.9 | 3.8 | 142.0 | 153.0 |
| Leonberger | 15 | 150.0 | 1.7 | 149.8 | 2.9 | 147.8 | 152.9 |
| Newfoundland | 29 | 149.4 | 2.1 | 150.0 | 2.6 | 145.0 | 152.4 |
| Retriever | 36 | 148.4 | 2.7 | 148.9 | 3.3 | 142.0 | 152.0 |
| Rottweiler | 49 | 148.5 | 2.5 | 149.0 | 2.5 | 143.0 | 152.9 |
|  |  |  |  |  |  |  |  |
| **Herding** |  |  |  |  |  |  |  |
| Border Collie | 73 | 149.0 | 2.4 | 149.1 | 3.0 | 143.0 | 153.0 |
| Collie | 14 | 148.6 | 3.6 | 148.2 | 5.9 | 142.0 | 153.0 |
| Shetland Sheepdog | 11 | 149.1 | 2.0 | 149.2 | 3.2 | 146.1 | 152.0 |
|  |  |  |  |  |  |  |  |
| **Terrier** |  |  |  |  |  |  |  |
| Airedale | 10 | 149.5 | 3.2 | 150.6 | 2.7 | 142.0 | 153.0 |
| Border Terrier | 35 | 147.7 | 2.5 | 148.0 | 3.0 | 143.0 | 152.0 |
| Norfolk Terrier | 12 | 148.1 | 2.4 | 148.0 | 2.3 | 143.0 | 152.0 |
| West Highland White Terrier | 78 | 149.1 | 2.5 | 149.3 | 4.0 | 143.0 | 153.0 |
| Yorkshire Terrier | 41 | 149.3 | 2.5 | 149.3 | 3.4 | 144.0 | 153.0 |
|  |  |  |  |  |  |  |  |
| **Scent hound** |  |  |  |  |  |  |  |
| Basset Hound | 21 | 148.9 | 2.5 | 148.9 | 3.7 | 144.0 | 153.0 |
| Beagle | 29 | 148.5 | 2.8 | 149.0 | 4.9 | 143.0 | 153.0 |
| Dachshund | 34 | 149.0 | 2.4 | 149.4 | 3.5 | 145.0 | 153.0 |
| Rhodesian Ridgeback | 32 | 149.0 | 1.9 | 149.0 | 2.2 | 146.0 | 153.0 |
|  |  |  |  |  |  |  |  |
| **Spaniel/Pointer** |  |  |  |  |  |  |  |
| Cavalier King Charles Spaniel | 174 | 148.8 | 2.3 | 149.0 | 3.1 | 142.0 | 153.0 |
| Cocker Spaniel | 98 | 148.4 | 2.5 | 148.5 | 3.6 | 142.0 | 153.0 |
| English Springer Spaniel | 41 | 148.8 | 2.6 | 149.5 | 4.2 | 143.2 | 153.0 |
| German Shorthaired Pointer | 19 | 147.8 | 2.8 | 147.0 | 3.7 | 143.0 | 153.0 |
| Gordon Setter | 14 | 146.9 | 2.9 | 147.0 | 4.3 | 142.0 | 151.0 |
| Hungarian Vizsla | 15 | 148.4 | 2.6 | 148.8 | 3.5 | 143.0 | 152.0 |
| Irish Setter | 23 | 149.3 | 2.1 | 150.0 | 3.4 | 145.0 | 152.0 |
| Italian Spinone | 21 | 149.4 | 1.7 | 149.8 | 3.0 | 145.9 | 152.0 |
| Pointer | 13 | 147.5 | 3.4 | 147.0 | 2.5 | 142.0 | 152.5 |
| Springer Spaniel | 71 | 148.6 | 2.4 | 149.0 | 2.7 | 142.0 | 152.9 |
| Weimaraner | 39 | 148.7 | 2.4 | 149.0 | 3.7 | 142.0 | 153.0 |
|  |  |  |  |  |  |  |  |
| **Other** |  |  |  |  |  |  |  |
| Bichon Frise | 14 | 148.2 | 2.9 | 149.0 | 2.0 | 143.0 | 152.6 |
| Collie cross | 16 | 148.8 | 3.0 | 149.1 | 4.5 | 142.4 | 152.9 |
| Dalmatian | 21 | 149.6 | 1.7 | 150.0 | 2.0 | 146.0 | 152.0 |
| Jack Russell Terrier | 78 | 148.7 | 2.3 | 149.0 | 3.0 | 142.0 | 153.0 |
| Labrador cross | 15 | 149.5 | 2.0 | 149.7 | 2.3 | 145.3 | 152.6 |
| Lhasa Apso | 17 | 147.4 | 2.5 | 148.0 | 3.8 | 143.0 | 152.0 |
| Poodle | 18 | 149.4 | 2.8 | 149.5 | 3.8 | 142.0 | 153.0 |
| Standard Poodle | 11 | 147.9 | 2.6 | 148.0 | 2.7 | 142.0 | 151.0 |

§ Unit of measurement: mmol/L; SD = standard deviation; IQR = interquartile range; Min. = minimum value recorded; Max. = maximum value recorded.

**Table E in S1 File. Descriptive statistics – potassium^§^**

| **Breed** | **N** | **Mean** | **SD** | **Median** | **IQR** | **Min.** | **Max.** |
| --- | --- | --- | --- | --- | --- | --- | --- |
| Mixed breed | 242 | 4.64 | 0.33 | 4.60 | 0.40 | 4.00 | 5.50 |
|  |  |  |  |  |  |  |  |
| **Ancient** |  |  |  |  |  |  |  |
| Maltese Terrier | 16 | 4.53 | 0.26 | 4.60 | 0.40 | 4.10 | 4.90 |
| Shar Pei | 19 | 4.58 | 0.30 | 4.60 | 0.20 | 4.00 | 5.20 |
| Siberian Husky | 12 | 4.91 | 0.38 | 4.90 | 0.63 | 4.30 | 5.40 |
| Tibetan Terrier | 17 | 4.73 | 0.32 | 4.80 | 0.60 | 4.30 | 5.30 |
|  |  |  |  |  |  |  |  |
| **Toy** |  |  |  |  |  |  |  |
| Chihuahua | 14 | 4.56 | 0.32 | 4.70 | 0.43 | 3.90 | 5.00 |
| Pug | 28 | 4.95 | 0.26 | 4.95 | 0.40 | 4.40 | 5.40 |
| Shih Tzu | 29 | 4.57 | 0.25 | 4.60 | 0.30 | 4.10 | 5.20 |
|  |  |  |  |  |  |  |  |
| **Working** |  |  |  |  |  |  |  |
| Dobermann | 77 | 4.62 | 0.31 | 4.60 | 0.40 | 3.90 | 5.40 |
| German Shepherd Dog | 160 | 4.65 | 0.31 | 4.60 | 0.40 | 3.90 | 5.50 |
| Miniature Schnauzer | 24 | 4.55 | 0.32 | 4.50 | 0.40 | 4.20 | 5.40 |
| Schnauzer | 13 | 4.91 | 0.31 | 5.00 | 0.40 | 4.40 | 5.40 |
|  |  |  |  |  |  |  |  |
| **Sight hound** |  |  |  |  |  |  |  |
| Greyhound | 14 | 4.52 | 0.31 | 4.50 | 0.48 | 3.90 | 4.90 |
| Lurcher | 19 | 4.35 | 0.30 | 4.30 | 0.30 | 3.90 | 5.20 |
|  |  |  |  |  |  |  |  |
| **Mastiff-like** |  |  |  |  |  |  |  |
| Boston Terrier | 15 | 4.64 | 0.25 | 4.70 | 0.30 | 4.20 | 5.10 |
| Boxer | 146 | 4.57 | 0.32 | 4.60 | 0.50 | 3.90 | 5.30 |
| Bullmastiff | 23 | 4.85 | 0.35 | 4.80 | 0.45 | 4.10 | 5.50 |
| Bulldog | 39 | 4.84 | 0.25 | 4.80 | 0.30 | 4.30 | 5.40 |
| Dogue de Bordeaux | 13 | 4.62 | 0.31 | 4.70 | 0.40 | 4.00 | 5.10 |
| French Bulldog | 20 | 4.72 | 0.33 | 4.70 | 0.43 | 4.10 | 5.40 |
| Mastiff | 13 | 4.72 | 0.33 | 4.80 | 0.40 | 4.10 | 5.40 |
| Staffordshire Bull Terrier | 95 | 4.70 | 0.36 | 4.70 | 0.40 | 4.00 | 5.50 |
|  |  |  |  |  |  |  |  |
| **Retriever/other Mastiff-like** |  |  |  |  |  |  |  |
| Flat Coated Retriever | 12 | 4.84 | 0.35 | 4.85 | 0.33 | 4.00 | 5.30 |
| Golden Retriever | 121 | 4.64 | 0.32 | 4.60 | 0.50 | 3.90 | 5.30 |
| Great Dane | 25 | 4.68 | 0.31 | 4.70 | 0.30 | 4.00 | 5.30 |
| Labrador Retriever | 327 | 4.66 | 0.31 | 4.70 | 0.50 | 3.90 | 5.50 |
| Leonberger | 15 | 4.74 | 0.40 | 4.70 | 0.60 | 4.00 | 5.30 |
| Newfoundland | 29 | 4.72 | 0.33 | 4.80 | 0.40 | 4.10 | 5.40 |
| Retriever | 36 | 4.74 | 0.36 | 4.80 | 0.40 | 3.90 | 5.50 |
| Rottweiler | 49 | 4.67 | 0.30 | 4.70 | 0.40 | 4.00 | 5.20 |
|  |  |  |  |  |  |  |  |
| **Herding** |  |  |  |  |  |  |  |
| Border Collie | 73 | 4.55 | 0.33 | 4.50 | 0.50 | 4.00 | 5.40 |
| Collie | 14 | 4.55 | 0.38 | 4.60 | 0.50 | 3.90 | 5.20 |
| Shetland Sheepdog | 11 | 4.65 | 0.33 | 4.70 | 0.15 | 4.20 | 5.50 |
|  |  |  |  |  |  |  |  |
| **Terrier** |  |  |  |  |  |  |  |
| Airedale | 10 | 4.59 | 0.31 | 4.60 | 0.48 | 4.10 | 5.00 |
| Border Terrier | 35 | 4.52 | 0.36 | 4.50 | 0.50 | 3.90 | 5.20 |
| Norfolk Terrier | 12 | 4.65 | 0.33 | 4.65 | 0.35 | 4.10 | 5.30 |
| West Highland White Terrier | 78 | 4.74 | 0.38 | 4.80 | 0.50 | 3.90 | 5.50 |
| Yorkshire Terrier | 41 | 4.48 | 0.28 | 4.40 | 0.40 | 4.00 | 5.40 |
|  |  |  |  |  |  |  |  |
| **Scent hound** |  |  |  |  |  |  |  |
| Basset Hound | 21 | 4.64 | 0.28 | 4.60 | 0.50 | 4.20 | 5.20 |
| Beagle | 29 | 4.63 | 0.35 | 4.60 | 0.50 | 3.90 | 5.40 |
| Dachshund | 34 | 4.62 | 0.26 | 4.60 | 0.30 | 4.20 | 5.30 |
| Rhodesian Ridgeback | 32 | 4.63 | 0.34 | 4.60 | 0.42 | 4.00 | 5.30 |
|  |  |  |  |  |  |  |  |
| **Spaniel/Pointer** |  |  |  |  |  |  |  |
| Cavalier King Charles Spaniel | 174 | 4.55 | 0.28 | 4.60 | 0.48 | 4.00 | 5.40 |
| Cocker Spaniel | 98 | 4.63 | 0.36 | 4.60 | 0.40 | 3.90 | 5.40 |
| English Springer Spaniel | 41 | 4.61 | 0.26 | 4.70 | 0.30 | 4.00 | 5.00 |
| German Shorthaired Pointer | 19 | 4.71 | 0.19 | 4.70 | 0.25 | 4.50 | 5.10 |
| Gordon Setter | 14 | 4.85 | 0.33 | 4.85 | 0.35 | 4.10 | 5.40 |
| Hungarian Vizsla | 15 | 4.51 | 0.24 | 4.60 | 0.35 | 4.00 | 4.80 |
| Irish Setter | 23 | 4.52 | 0.26 | 4.50 | 0.20 | 4.00 | 5.10 |
| Italian Spinone | 21 | 4.86 | 0.27 | 4.90 | 0.30 | 4.30 | 5.30 |
| Pointer | 13 | 4.82 | 0.32 | 4.90 | 0.40 | 4.30 | 5.30 |
| Springer Spaniel | 71 | 4.66 | 0.27 | 4.70 | 0.30 | 4.00 | 5.30 |
| Weimaraner | 39 | 4.70 | 0.38 | 4.70 | 0.60 | 4.00 | 5.50 |
|  |  |  |  |  |  |  |  |
| **Other** |  |  |  |  |  |  |  |
| Bichon Frise | 14 | 4.57 | 0.36 | 4.55 | 0.35 | 3.90 | 5.30 |
| Collie cross | 16 | 4.53 | 0.23 | 4.50 | 0.23 | 4.00 | 4.90 |
| Dalmatian | 21 | 4.76 | 0.30 | 4.80 | 0.30 | 4.20 | 5.40 |
| Jack Russell Terrier | 78 | 4.56 | 0.35 | 4.55 | 0.40 | 3.90 | 5.50 |
| Labrador cross | 15 | 4.57 | 0.30 | 4.60 | 0.50 | 4.10 | 5.00 |
| Lhasa Apso | 17 | 4.65 | 0.33 | 4.60 | 0.30 | 4.20 | 5.30 |
| Poodle | 18 | 4.71 | 0.24 | 4.75 | 0.27 | 4.40 | 5.30 |
| Standard Poodle | 11 | 4.65 | 0.25 | 4.60 | 0.40 | 4.20 | 5.00 |

§ Unit of measurement: mmol/L; SD = standard deviation; IQR = interquartile range; Min. = minimum value recorded; Max. = maximum value recorded.

**Table F in S1 File. Descriptive statistics – chloride^§^**

| **Breed** | **N** | **Mean** | **SD** | **Median** | **IQR** | **Min.** | **Max.** |
| --- | --- | --- | --- | --- | --- | --- | --- |
| Mixed breed | 242 | 112.3 | 2.4 | 112.0 | 3.6 | 106.0 | 118.0 |
|  |  |  |  |  |  |  |  |
| **Ancient** |  |  |  |  |  |  |  |
| Maltese Terrier | 16 | 111.1 | 2.8 | 111.0 | 2.7 | 107.0 | 116.0 |
| Shar Pei | 19 | 111.5 | 2.4 | 112.0 | 3.0 | 106.0 | 116.0 |
| Siberian Husky | 12 | 113.3 | 2.4 | 113.7 | 3.3 | 109.0 | 116.7 |
| Tibetan Terrier | 17 | 112.0 | 2.7 | 112.0 | 3.1 | 105.0 | 116.0 |
|  |  |  |  |  |  |  |  |
| **Toy** |  |  |  |  |  |  |  |
| Chihuahua | 14 | 111.5 | 2.9 | 112.4 | 4.6 | 106.0 | 115.0 |
| Pug | 28 | 108.8 | 1.9 | 109.0 | 2.5 | 105.0 | 113.0 |
| Shih Tzu | 29 | 110.2 | 2.0 | 110.1 | 2.9 | 106.0 | 114.0 |
|  |  |  |  |  |  |  |  |
| **Working** |  |  |  |  |  |  |  |
| Dobermann | 77 | 111.8 | 2.2 | 112.0 | 3.0 | 106.5 | 116.0 |
| German Shepherd Dog | 160 | 113.0 | 2.3 | 113.0 | 2.1 | 105.6 | 118.0 |
| Miniature Schnauzer | 24 | 111.1 | 2.8 | 111.0 | 3.8 | 105.0 | 116.7 |
| Schnauzer | 13 | 112.0 | 2.8 | 111.3 | 4.4 | 107.4 | 116.0 |
|  |  |  |  |  |  |  |  |
| **Sight hound** |  |  |  |  |  |  |  |
| Greyhound | 14 | 112.2 | 1.6 | 112.4 | 1.8 | 109.4 | 115.0 |
| Lurcher | 19 | 112.6 | 2.8 | 113.0 | 3.4 | 107.0 | 117.0 |
|  |  |  |  |  |  |  |  |
| **Mastiff-like** |  |  |  |  |  |  |  |
| Boston Terrier | 15 | 110.1 | 2.8 | 110.0 | 4.4 | 107.0 | 116.4 |
| Boxer | 146 | 111.9 | 2.0 | 112.0 | 2.1 | 106.0 | 117.1 |
| Bullmastiff | 23 | 110.5 | 2.3 | 110.0 | 1.7 | 105.3 | 116.3 |
| Bulldog | 39 | 110.6 | 2.1 | 111.0 | 3.1 | 106.1 | 115.0 |
| Dogue de Bordeaux | 13 | 110.5 | 2.8 | 110.2 | 4.0 | 107.0 | 115.9 |
| French Bulldog | 20 | 109.7 | 2.3 | 110.0 | 2.9 | 105.2 | 114.0 |
| Mastiff | 13 | 110.1 | 2.3 | 110.0 | 1.5 | 105.9 | 113.0 |
| Staffordshire Bull Terrier | 95 | 110.8 | 2.4 | 111.0 | 2.7 | 105.0 | 115.8 |
|  |  |  |  |  |  |  |  |
| **Retriever/other Mastiff-like** |  |  |  |  |  |  |  |
| Flat Coated Retriever | 12 | 113.1 | 2.9 | 113.0 | 4.3 | 108.0 | 117.4 |
| Golden Retriever | 121 | 111.8 | 2.2 | 112.0 | 3.0 | 105.0 | 117.0 |
| Great Dane | 25 | 112.7 | 2.2 | 113.0 | 2.9 | 108.7 | 117.0 |
| Labrador Retriever | 327 | 112.4 | 2.0 | 112.0 | 2.7 | 105.0 | 117.9 |
| Leonberger | 15 | 113.7 | 1.8 | 114.0 | 2.6 | 111.0 | 117.1 |
| Newfoundland | 29 | 111.1 | 2.2 | 111.0 | 3.1 | 105.0 | 114.0 |
| Retriever | 36 | 112.3 | 1.9 | 112.0 | 3.0 | 107.2 | 116.0 |
| Rottweiler | 49 | 111.2 | 2.0 | 111.0 | 2.1 | 107.8 | 115.7 |
|  |  |  |  |  |  |  |  |
| **Herding** |  |  |  |  |  |  |  |
| Border Collie | 73 | 112.9 | 2.2 | 113.0 | 2.9 | 107.0 | 117.1 |
| Collie | 14 | 112.9 | 2.5 | 112.2 | 3.4 | 108.0 | 117.6 |
| Shetland Sheepdog | 11 | 111.0 | 2.1 | 111.0 | 2.4 | 108.0 | 115.6 |
|  |  |  |  |  |  |  |  |
| **Terrier** |  |  |  |  |  |  |  |
| Airedale | 10 | 110.6 | 3.0 | 111.4 | 4.7 | 106.4 | 115.3 |
| Border Terrier | 35 | 110.5 | 1.8 | 110.0 | 2.4 | 106.8 | 115.0 |
| Norfolk Terrier | 12 | 111.1 | 2.2 | 110.5 | 2.6 | 108.0 | 115.1 |
| West Highland White Terrier | 78 | 111.0 | 2.5 | 111.0 | 2.8 | 105.4 | 116.4 |
| Yorkshire Terrier | 41 | 112.2 | 2.4 | 112.5 | 3.0 | 106.0 | 116.7 |
|  |  |  |  |  |  |  |  |
| **Scent hound** |  |  |  |  |  |  |  |
| Basset Hound | 21 | 111.2 | 2.7 | 111.0 | 2.2 | 107.0 | 117.4 |
| Beagle | 29 | 111.8 | 2.5 | 111.7 | 2.2 | 106.4 | 118.0 |
| Dachshund | 34 | 111.7 | 2.3 | 112.0 | 3.4 | 105.2 | 115.0 |
| Rhodesian Ridgeback | 32 | 111.1 | 2.1 | 111.0 | 2.9 | 108.0 | 115.0 |
|  |  |  |  |  |  |  |  |
| **Spaniel/Pointer** |  |  |  |  |  |  |  |
| Cavalier King Charles Spaniel | 174 | 110.4 | 2.4 | 110.5 | 2.7 | 105.0 | 118.0 |
| Cocker Spaniel | 98 | 111.6 | 2.5 | 112.0 | 3.2 | 105.0 | 117.0 |
| English Springer Spaniel | 41 | 112.4 | 2.0 | 112.6 | 3.0 | 106.8 | 115.6 |
| German Shorthaired Pointer | 19 | 111.9 | 2.0 | 112.0 | 2.4 | 108.0 | 116.9 |
| Gordon Setter | 14 | 112.7 | 2.2 | 113.0 | 3.0 | 110.0 | 118.0 |
| Hungarian Vizsla | 15 | 112.7 | 2.9 | 113.0 | 2.4 | 107.0 | 117.0 |
| Irish Setter | 23 | 112.7 | 1.7 | 112.1 | 1.3 | 110.1 | 117.0 |
| Italian Spinone | 21 | 112.4 | 1.9 | 112.2 | 2.4 | 107.5 | 116.1 |
| Pointer | 13 | 111.6 | 2.9 | 111.0 | 4.0 | 107.2 | 116.0 |
| Springer Spaniel | 71 | 111.7 | 2.3 | 112.0 | 2.3 | 105.0 | 116.0 |
| Weimaraner | 39 | 112.9 | 2.0 | 113.0 | 2.0 | 107.7 | 117.2 |
|  |  |  |  |  |  |  |  |
| **Other** |  |  |  |  |  |  |  |
| Bichon Frise | 14 | 112.1 | 3.1 | 112.0 | 2.6 | 107.4 | 117.0 |
| Collie cross | 16 | 112.8 | 1.7 | 113.0 | 2.3 | 110.0 | 115.5 |
| Dalmatian | 21 | 112.1 | 1.5 | 112.0 | 2.0 | 109.8 | 114.6 |
| Jack Russell Terrier | 78 | 111.9 | 2.5 | 112.0 | 3.9 | 105.0 | 117.0 |
| Labrador cross | 15 | 112.4 | 2.1 | 112.4 | 2.0 | 108.0 | 117.0 |
| Lhasa Apso | 17 | 110.6 | 2.9 | 112.0 | 3.4 | 105.0 | 114.1 |
| Poodle | 18 | 113.4 | 1.7 | 113.5 | 1.8 | 110.0 | 116.0 |
| Standard Poodle | 11 | 113.6 | 1.3 | 114.0 | 2.0 | 111.0 | 115.0 |

§ Unit of measurement: mmol/L; SD = standard deviation; IQR = interquartile range; Min. = minimum value recorded; Max. = maximum value recorded.

**Table G in S1 File. Descriptive statistics – calcium^§^**

| **Breed** | **N** | **Mean** | **SD** | **Median** | **IQR** | **Min.** | **Max.** |
| --- | --- | --- | --- | --- | --- | --- | --- |
| Mixed breed | 242 | 2.48 | 0.12 | 2.49 | 0.18 | 2.15 | 2.70 |
|  |  |  |  |  |  |  |  |
| **Ancient** |  |  |  |  |  |  |  |
| Maltese Terrier | 16 | 2.44 | 0.13 | 2.44 | 0.19 | 2.17 | 2.62 |
| Shar Pei | 19 | 2.42 | 0.13 | 2.42 | 0.21 | 2.25 | 2.67 |
| Siberian Husky | 12 | 2.47 | 0.09 | 2.45 | 0.08 | 2.32 | 2.69 |
| Tibetan Terrier | 17 | 2.52 | 0.10 | 2.52 | 0.11 | 2.30 | 2.67 |
|  |  |  |  |  |  |  |  |
| **Toy** |  |  |  |  |  |  |  |
| Chihuahua | 14 | 2.51 | 0.11 | 2.52 | 0.12 | 2.31 | 2.67 |
| Pug | 28 | 2.52 | 0.13 | 2.54 | 0.16 | 2.24 | 2.70 |
| Shih Tzu | 29 | 2.48 | 0.14 | 2.51 | 0.18 | 2.16 | 2.66 |
|  |  |  |  |  |  |  |  |
| **Working** |  |  |  |  |  |  |  |
| Dobermann | 77 | 2.48 | 0.11 | 2.50 | 0.16 | 2.22 | 2.68 |
| German Shepherd Dog | 160 | 2.50 | 0.11 | 2.51 | 0.15 | 2.16 | 2.70 |
| Miniature Schnauzer | 24 | 2.51 | 0.09 | 2.53 | 0.11 | 2.28 | 2.63 |
| Schnauzer | 13 | 2.49 | 0.11 | 2.50 | 0.12 | 2.33 | 2.67 |
|  |  |  |  |  |  |  |  |
| **Sight hound** |  |  |  |  |  |  |  |
| Greyhound | 14 | 2.46 | 0.13 | 2.46 | 0.16 | 2.28 | 2.69 |
| Lurcher | 19 | 2.43 | 0.11 | 2.44 | 0.17 | 2.24 | 2.67 |
|  |  |  |  |  |  |  |  |
| **Mastiff-like** |  |  |  |  |  |  |  |
| Boston Terrier | 15 | 2.30 | 0.12 | 2.30 | 0.15 | 2.13 | 2.54 |
| Boxer | 146 | 2.46 | 0.10 | 2.47 | 0.13 | 2.18 | 2.70 |
| Bullmastiff | 23 | 2.49 | 0.13 | 2.51 | 0.13 | 2.16 | 2.66 |
| Bulldog | 39 | 2.47 | 0.12 | 2.47 | 0.16 | 2.13 | 2.70 |
| Dogue de Bordeaux | 13 | 2.48 | 0.11 | 2.45 | 0.20 | 2.32 | 2.64 |
| French Bulldog | 20 | 2.48 | 0.08 | 2.49 | 0.11 | 2.32 | 2.63 |
| Mastiff | 13 | 2.52 | 0.10 | 2.49 | 0.12 | 2.34 | 2.70 |
| Staffordshire Bull Terrier | 95 | 2.48 | 0.12 | 2.50 | 0.14 | 2.13 | 2.70 |
|  |  |  |  |  |  |  |  |
| **Retriever/other Mastiff-like** |  |  |  |  |  |  |  |
| Flat Coated Retriever | 12 | 2.39 | 0.09 | 2.40 | 0.08 | 2.23 | 2.53 |
| Golden Retriever | 121 | 2.47 | 0.11 | 2.48 | 0.15 | 2.15 | 2.69 |
| Great Dane | 25 | 2.48 | 0.14 | 2.46 | 0.17 | 2.20 | 2.70 |
| Labrador Retriever | 327 | 2.46 | 0.12 | 2.47 | 0.15 | 2.15 | 2.70 |
| Leonberger | 15 | 2.49 | 0.13 | 2.49 | 0.21 | 2.28 | 2.66 |
| Newfoundland | 29 | 2.50 | 0.13 | 2.53 | 0.16 | 2.26 | 2.70 |
| Retriever | 36 | 2.46 | 0.09 | 2.45 | 0.14 | 2.25 | 2.64 |
| Rottweiler | 49 | 2.47 | 0.11 | 2.45 | 0.15 | 2.16 | 2.69 |
|  |  |  |  |  |  |  |  |
| **Herding** |  |  |  |  |  |  |  |
| Border Collie | 73 | 2.47 | 0.10 | 2.47 | 0.14 | 2.27 | 2.70 |
| Collie | 14 | 2.46 | 0.10 | 2.48 | 0.06 | 2.18 | 2.63 |
| Shetland Sheepdog | 11 | 2.44 | 0.12 | 2.40 | 0.19 | 2.31 | 2.64 |
|  |  |  |  |  |  |  |  |
| **Terrier** |  |  |  |  |  |  |  |
| Airedale | 10 | 2.60 | 0.06 | 2.62 | 0.10 | 2.52 | 2.67 |
| Border Terrier | 35 | 2.47 | 0.11 | 2.47 | 0.14 | 2.18 | 2.69 |
| Norfolk Terrier | 12 | 2.53 | 0.12 | 2.56 | 0.16 | 2.28 | 2.69 |
| West Highland White Terrier | 78 | 2.50 | 0.10 | 2.50 | 0.14 | 2.26 | 2.69 |
| Yorkshire Terrier | 41 | 2.47 | 0.10 | 2.47 | 0.12 | 2.26 | 2.67 |
|  |  |  |  |  |  |  |  |
| **Scent hound** |  |  |  |  |  |  |  |
| Basset Hound | 21 | 2.49 | 0.11 | 2.51 | 0.10 | 2.26 | 2.65 |
| Beagle | 29 | 2.50 | 0.11 | 2.52 | 0.12 | 2.27 | 2.68 |
| Dachshund | 34 | 2.44 | 0.14 | 2.41 | 0.23 | 2.14 | 2.68 |
| Rhodesian Ridgeback | 32 | 2.53 | 0.09 | 2.54 | 0.09 | 2.34 | 2.68 |
|  |  |  |  |  |  |  |  |
| **Spaniel/Pointer** |  |  |  |  |  |  |  |
| Cavalier King Charles Spaniel | 174 | 2.49 | 0.10 | 2.50 | 0.14 | 2.13 | 2.68 |
| Cocker Spaniel | 98 | 2.50 | 0.11 | 2.50 | 0.13 | 2.13 | 2.69 |
| English Springer Spaniel | 41 | 2.51 | 0.10 | 2.51 | 0.13 | 2.25 | 2.70 |
| German Shorthaired Pointer | 19 | 2.41 | 0.11 | 2.42 | 0.14 | 2.15 | 2.58 |
| Gordon Setter | 14 | 2.42 | 0.12 | 2.44 | 0.09 | 2.15 | 2.63 |
| Hungarian Vizsla | 15 | 2.40 | 0.10 | 2.42 | 0.09 | 2.20 | 2.51 |
| Irish Setter | 23 | 2.45 | 0.10 | 2.45 | 0.11 | 2.18 | 2.64 |
| Italian Spinone | 21 | 2.50 | 0.14 | 2.51 | 0.10 | 2.14 | 2.70 |
| Pointer | 13 | 2.45 | 0.11 | 2.46 | 0.09 | 2.24 | 2.65 |
| Springer Spaniel | 71 | 2.47 | 0.12 | 2.49 | 0.17 | 2.14 | 2.65 |
| Weimaraner | 39 | 2.48 | 0.12 | 2.48 | 0.15 | 2.19 | 2.68 |
|  |  |  |  |  |  |  |  |
| **Other** |  |  |  |  |  |  |  |
| Bichon Frise | 14 | 2.53 | 0.12 | 2.54 | 0.16 | 2.25 | 2.69 |
| Collie cross | 16 | 2.47 | 0.14 | 2.47 | 0.24 | 2.19 | 2.63 |
| Dalmatian | 21 | 2.44 | 0.14 | 2.47 | 0.15 | 2.16 | 2.64 |
| Jack Russell Terrier | 78 | 2.47 | 0.11 | 2.49 | 0.14 | 2.19 | 2.69 |
| Labrador cross | 15 | 2.46 | 0.13 | 2.48 | 0.18 | 2.19 | 2.68 |
| Lhasa Apso | 17 | 2.48 | 0.08 | 2.49 | 0.10 | 2.32 | 2.64 |
| Poodle | 18 | 2.45 | 0.10 | 2.45 | 0.11 | 2.24 | 2.68 |
| Standard Poodle | 11 | 2.50 | 0.11 | 2.53 | 0.12 | 2.30 | 2.65 |

§ Unit of measurement: mmol/L; SD = standard deviation; IQR = interquartile range; Min. = minimum value recorded; Max. = maximum value recorded.

**Table H in S1 File. Descriptive statistics – phosphorus^§^**

| **Breed** | **N** | **Mean** | **SD** | **Median** | **IQR** | **Min.** | **Max.** |
| --- | --- | --- | --- | --- | --- | --- | --- |
| Mixed breed | 242 | 1.26 | 0.24 | 1.25 | 0.35 | 0.80 | 1.89 |
|  |  |  |  |  |  |  |  |
| **Ancient** |  |  |  |  |  |  |  |
| Maltese Terrier | 16 | 1.28 | 0.33 | 1.20 | 0.47 | 0.87 | 1.95 |
| Shar Pei | 19 | 1.22 | 0.15 | 1.23 | 0.16 | 0.88 | 1.49 |
| Siberian Husky | 12 | 1.28 | 0.26 | 1.29 | 0.35 | 0.91 | 1.70 |
| Tibetan Terrier | 17 | 1.25 | 0.30 | 1.22 | 0.28 | 0.89 | 2.00 |
|  |  |  |  |  |  |  |  |
| **Toy** |  |  |  |  |  |  |  |
| Chihuahua | 14 | 1.22 | 0.29 | 1.09 | 0.45 | 0.91 | 1.80 |
| Pug | 28 | 1.36 | 0.33 | 1.34 | 0.55 | 0.85 | 1.97 |
| Shih Tzu | 29 | 1.28 | 0.23 | 1.28 | 0.20 | 0.92 | 1.95 |
|  |  |  |  |  |  |  |  |
| **Working** |  |  |  |  |  |  |  |
| Dobermann | 77 | 1.38 | 0.28 | 1.36 | 0.38 | 0.84 | 1.97 |
| German Shepherd Dog | 160 | 1.30 | 0.26 | 1.28 | 0.34 | 0.80 | 1.95 |
| Miniature Schnauzer | 24 | 1.34 | 0.24 | 1.34 | 0.30 | 0.97 | 1.95 |
| Schnauzer | 13 | 1.36 | 0.27 | 1.36 | 0.22 | 0.83 | 1.94 |
|  |  |  |  |  |  |  |  |
| **Sight hound** |  |  |  |  |  |  |  |
| Greyhound | 14 | 1.37 | 0.28 | 1.38 | 0.35 | 0.89 | 1.81 |
| Lurcher | 19 | 1.24 | 0.21 | 1.30 | 0.35 | 0.94 | 1.70 |
|  |  |  |  |  |  |  |  |
| **Mastiff-like** |  |  |  |  |  |  |  |
| Boston Terrier | 15 | 1.30 | 0.24 | 1.25 | 0.30 | 0.90 | 1.75 |
| Boxer | 146 | 1.46 | 0.23 | 1.45 | 0.35 | 0.92 | 1.96 |
| Bullmastiff | 23 | 1.38 | 0.21 | 1.41 | 0.27 | 1.03 | 1.76 |
| Bulldog | 39 | 1.52 | 0.27 | 1.48 | 0.41 | 0.82 | 2.00 |
| Dogue de Bordeaux | 13 | 1.59 | 0.20 | 1.55 | 0.27 | 1.35 | 1.92 |
| French Bulldog | 20 | 1.47 | 0.24 | 1.44 | 0.21 | 0.98 | 1.94 |
| Mastiff | 13 | 1.20 | 0.31 | 1.19 | 0.46 | 0.85 | 1.71 |
| Staffordshire Bull Terrier | 95 | 1.22 | 0.28 | 1.20 | 0.38 | 0.80 | 1.94 |
|  |  |  |  |  |  |  |  |
| **Retriever/other Mastiff-like** |  |  |  |  |  |  |  |
| Flat Coated Retriever | 12 | 1.41 | 0.30 | 1.44 | 0.41 | 0.95 | 1.89 |
| Golden Retriever | 121 | 1.20 | 0.24 | 1.16 | 0.29 | 0.82 | 1.99 |
| Great Dane | 25 | 1.32 | 0.27 | 1.24 | 0.40 | 0.92 | 1.80 |
| Labrador Retriever | 327 | 1.28 | 0.25 | 1.25 | 0.32 | 0.80 | 2.00 |
| Leonberger | 15 | 1.38 | 0.23 | 1.37 | 0.31 | 0.97 | 1.69 |
| Newfoundland | 29 | 1.36 | 0.22 | 1.29 | 0.26 | 0.93 | 1.98 |
| Retriever | 36 | 1.21 | 0.24 | 1.22 | 0.31 | 0.80 | 1.80 |
| Rottweiler | 49 | 1.42 | 0.22 | 1.41 | 0.21 | 0.97 | 1.93 |
|  |  |  |  |  |  |  |  |
| **Herding** |  |  |  |  |  |  |  |
| Border Collie | 73 | 1.31 | 0.25 | 1.29 | 0.31 | 0.84 | 1.98 |
| Collie | 14 | 1.31 | 0.26 | 1.27 | 0.35 | 0.83 | 1.81 |
| Shetland Sheepdog | 11 | 1.04 | 0.14 | 1.02 | 0.14 | 0.87 | 1.36 |
|  |  |  |  |  |  |  |  |
| **Terrier** |  |  |  |  |  |  |  |
| Airedale | 10 | 1.31 | 0.21 | 1.38 | 0.25 | 0.94 | 1.60 |
| Border Terrier | 35 | 1.30 | 0.25 | 1.31 | 0.37 | 0.83 | 1.82 |
| Norfolk Terrier | 12 | 1.26 | 0.32 | 1.20 | 0.25 | 0.86 | 1.96 |
| West Highland White Terrier | 78 | 1.41 | 0.23 | 1.44 | 0.31 | 0.81 | 1.91 |
| Yorkshire Terrier | 41 | 1.26 | 0.27 | 1.20 | 0.33 | 0.92 | 1.96 |
|  |  |  |  |  |  |  |  |
| **Scent hound** |  |  |  |  |  |  |  |
| Basset Hound | 21 | 1.27 | 0.29 | 1.29 | 0.35 | 0.86 | 1.80 |
| Beagle | 29 | 1.38 | 0.25 | 1.36 | 0.30 | 0.99 | 1.99 |
| Dachshund | 34 | 1.27 | 0.24 | 1.31 | 0.31 | 0.82 | 1.72 |
| Rhodesian Ridgeback | 32 | 1.27 | 0.28 | 1.21 | 0.37 | 0.82 | 1.96 |
|  |  |  |  |  |  |  |  |
| **Spaniel/Pointer** |  |  |  |  |  |  |  |
| Cavalier King Charles Spaniel | 174 | 1.43 | 0.24 | 1.44 | 0.31 | 0.84 | 1.98 |
| Cocker Spaniel | 98 | 1.33 | 0.24 | 1.27 | 0.33 | 0.84 | 1.97 |
| English Springer Spaniel | 41 | 1.26 | 0.26 | 1.21 | 0.25 | 0.83 | 1.99 |
| German Shorthaired Pointer | 19 | 1.27 | 0.21 | 1.23 | 0.23 | 0.91 | 1.67 |
| Gordon Setter | 14 | 1.26 | 0.28 | 1.24 | 0.28 | 0.81 | 1.95 |
| Hungarian Vizsla | 15 | 1.35 | 0.23 | 1.28 | 0.22 | 1.01 | 1.79 |
| Irish Setter | 23 | 1.37 | 0.31 | 1.26 | 0.34 | 0.94 | 1.96 |
| Italian Spinone | 21 | 1.36 | 0.25 | 1.33 | 0.22 | 0.82 | 1.84 |
| Pointer | 13 | 1.38 | 0.25 | 1.45 | 0.32 | 1.00 | 1.77 |
| Springer Spaniel | 71 | 1.35 | 0.23 | 1.33 | 0.29 | 0.87 | 1.95 |
| Weimaraner | 39 | 1.30 | 0.29 | 1.24 | 0.39 | 0.86 | 1.94 |
|  |  |  |  |  |  |  |  |
| **Other** |  |  |  |  |  |  |  |
| Bichon Frise | 14 | 1.43 | 0.28 | 1.41 | 0.47 | 1.06 | 1.93 |
| Collie cross | 16 | 1.28 | 0.17 | 1.25 | 0.28 | 1.09 | 1.63 |
| Dalmatian | 21 | 1.47 | 0.21 | 1.41 | 0.29 | 1.08 | 1.91 |
| Jack Russell Terrier | 78 | 1.30 | 0.26 | 1.32 | 0.41 | 0.84 | 1.95 |
| Labrador cross | 15 | 1.24 | 0.23 | 1.22 | 0.33 | 0.88 | 1.62 |
| Lhasa Apso | 17 | 1.44 | 0.28 | 1.36 | 0.34 | 1.03 | 1.92 |
| Poodle | 18 | 1.40 | 0.19 | 1.39 | 0.16 | 0.99 | 1.74 |
| Standard Poodle | 11 | 1.36 | 0.25 | 1.30 | 0.28 | 0.98 | 1.86 |

§ Unit of measurement: mmol/L; SD = standard deviation; IQR = interquartile range; Min. = minimum value recorded; Max. = maximum value recorded.

**Table I in S1 File. Descriptive statistics – urea^§^**

| **Breed** | **N** | **Mean** | **SD** | **Median** | **IQR** | **Min.** | **Max.** |
| --- | --- | --- | --- | --- | --- | --- | --- |
| Mixed breed | 242 | 5.3 | 1.5 | 5.2 | 2.2 | 3.0 | 9.1 |
|  |  |  |  |  |  |  |  |
| **Ancient** |  |  |  |  |  |  |  |
| Maltese Terrier | 16 | 5.8 | 1.2 | 5.9 | 1.8 | 3.6 | 7.5 |
| Shar Pei | 19 | 5.8 | 1.5 | 5.9 | 1.8 | 3.0 | 8.8 |
| Siberian Husky | 12 | 5.1 | 1.1 | 4.9 | 1.4 | 3.4 | 7.3 |
| Tibetan Terrier | 17 | 5.9 | 1.7 | 5.1 | 3.0 | 3.3 | 8.4 |
|  |  |  |  |  |  |  |  |
| **Toy** |  |  |  |  |  |  |  |
| Chihuahua | 14 | 6.6 | 1.5 | 6.6 | 2.4 | 4.3 | 9.1 |
| Pug | 28 | 5.7 | 1.2 | 5.8 | 1.3 | 3.3 | 8.7 |
| Shih Tzu | 29 | 5.7 | 1.7 | 6.0 | 2.5 | 3.0 | 9.0 |
|  |  |  |  |  |  |  |  |
| **Working** |  |  |  |  |  |  |  |
| Dobermann | 77 | 5.0 | 1.4 | 4.7 | 2.3 | 3.0 | 8.8 |
| German Shepherd Dog | 160 | 5.6 | 1.4 | 5.4 | 1.9 | 3.1 | 9.1 |
| Miniature Schnauzer | 24 | 4.9 | 1.0 | 4.8 | 1.1 | 3.2 | 7.6 |
| Schnauzer | 13 | 4.5 | 1.3 | 4.1 | 1.6 | 3.1 | 7.2 |
|  |  |  |  |  |  |  |  |
| **Sight hound** |  |  |  |  |  |  |  |
| Greyhound | 14 | 4.9 | 1.2 | 4.9 | 0.7 | 3.0 | 7.2 |
| Lurcher | 19 | 5.0 | 1.4 | 4.4 | 2.0 | 3.3 | 8.1 |
|  |  |  |  |  |  |  |  |
| **Mastiff-like** |  |  |  |  |  |  |  |
| Boston Terrier | 15 | 6.6 | 1.4 | 6.8 | 2.0 | 3.9 | 8.7 |
| Boxer | 146 | 4.9 | 1.2 | 4.7 | 1.6 | 3.0 | 8.5 |
| Bullmastiff | 23 | 4.9 | 1.1 | 4.8 | 1.6 | 3.1 | 7.2 |
| Bulldog | 39 | 5.1 | 1.7 | 4.8 | 2.5 | 3.0 | 9.1 |
| Dogue de Bordeaux | 13 | 5.4 | 1.4 | 5.1 | 1.4 | 3.7 | 8.6 |
| French Bulldog | 20 | 5.5 | 1.6 | 5.5 | 2.0 | 3.0 | 8.3 |
| Mastiff | 13 | 5.2 | 1.4 | 4.8 | 2.1 | 3.4 | 7.8 |
| Staffordshire Bull Terrier | 95 | 5.1 | 1.4 | 4.9 | 2.2 | 3.0 | 9.0 |
|  |  |  |  |  |  |  |  |
| **Retriever/other Mastiff-like** |  |  |  |  |  |  |  |
| Flat Coated Retriever | 12 | 5.1 | 1.2 | 5.1 | 1.7 | 3.6 | 7.2 |
| Golden Retriever | 121 | 4.8 | 1.4 | 4.5 | 1.8 | 3.0 | 9.0 |
| Great Dane | 25 | 5.2 | 1.6 | 4.6 | 2.0 | 3.3 | 9.0 |
| Labrador Retriever | 327 | 5.1 | 1.4 | 4.9 | 1.8 | 3.0 | 9.1 |
| Leonberger | 15 | 5.0 | 1.5 | 4.8 | 2.0 | 3.1 | 7.9 |
| Newfoundland | 29 | 4.7 | 1.3 | 4.6 | 2.0 | 3.0 | 7.4 |
| Retriever | 36 | 4.9 | 1.1 | 4.7 | 1.5 | 3.1 | 7.8 |
| Rottweiler | 49 | 5.1 | 1.1 | 5.1 | 1.6 | 3.0 | 7.6 |
|  |  |  |  |  |  |  |  |
| **Herding** |  |  |  |  |  |  |  |
| Border Collie | 73 | 5.2 | 1.2 | 5.3 | 1.7 | 3.0 | 9.0 |
| Collie | 14 | 5.9 | 1.3 | 5.8 | 1.5 | 4.3 | 8.2 |
| Shetland Sheepdog | 11 | 5.3 | 1.0 | 5.0 | 1.8 | 4.1 | 6.7 |
|  |  |  |  |  |  |  |  |
| **Terrier** |  |  |  |  |  |  |  |
| Airedale | 10 | 4.8 | 1.2 | 4.5 | 1.2 | 3.5 | 7.5 |
| Border Terrier | 35 | 5.0 | 1.2 | 4.9 | 1.9 | 3.0 | 7.7 |
| Norfolk Terrier | 12 | 4.8 | 0.9 | 4.8 | 1.3 | 3.4 | 6.6 |
| West Highland White Terrier | 78 | 5.2 | 1.4 | 4.9 | 2.1 | 3.0 | 8.9 |
| Yorkshire Terrier | 41 | 5.8 | 1.6 | 5.6 | 2.4 | 3.0 | 8.8 |
|  |  |  |  |  |  |  |  |
| **Scent hound** |  |  |  |  |  |  |  |
| Basset Hound | 21 | 4.8 | 1.5 | 4.8 | 1.8 | 3.0 | 8.4 |
| Beagle | 29 | 5.0 | 1.5 | 4.8 | 2.1 | 3.0 | 9.0 |
| Dachshund | 34 | 4.8 | 1.3 | 4.7 | 1.5 | 3.0 | 8.3 |
| Rhodesian Ridgeback | 32 | 4.9 | 1.5 | 4.7 | 2.1 | 3.0 | 9.0 |
|  |  |  |  |  |  |  |  |
| **Spaniel/Pointer** |  |  |  |  |  |  |  |
| Cavalier King Charles Spaniel | 174 | 4.8 | 1.2 | 4.6 | 1.6 | 3.1 | 8.5 |
| Cocker Spaniel | 98 | 4.9 | 1.2 | 4.7 | 1.6 | 3.0 | 8.5 |
| English Springer Spaniel | 41 | 4.9 | 1.3 | 4.6 | 1.5 | 3.2 | 8.9 |
| German Shorthaired Pointer | 19 | 5.3 | 1.0 | 5.3 | 1.5 | 3.3 | 7.2 |
| Gordon Setter | 14 | 4.5 | 0.8 | 4.3 | 1.3 | 3.5 | 5.9 |
| Hungarian Vizsla | 15 | 4.0 | 0.8 | 3.8 | 0.9 | 3.0 | 5.8 |
| Irish Setter | 23 | 4.6 | 1.0 | 4.6 | 1.2 | 3.0 | 7.2 |
| Italian Spinone | 21 | 6.2 | 1.3 | 6.4 | 2.1 | 3.8 | 8.6 |
| Pointer | 13 | 4.8 | 1.3 | 4.2 | 1.6 | 3.1 | 7.6 |
| Springer Spaniel | 71 | 5.0 | 1.0 | 4.8 | 1.4 | 3.1 | 7.2 |
| Weimaraner | 39 | 5.0 | 1.2 | 4.8 | 2.1 | 3.1 | 7.5 |
|  |  |  |  |  |  |  |  |
| **Other** |  |  |  |  |  |  |  |
| Bichon Frise | 14 | 6.2 | 1.5 | 6.4 | 1.5 | 3.0 | 8.6 |
| Collie cross | 16 | 5.3 | 1.3 | 5.2 | 1.8 | 3.1 | 7.8 |
| Dalmatian | 21 | 4.7 | 1.0 | 4.5 | 1.0 | 3.4 | 7.4 |
| Jack Russell Terrier | 78 | 5.3 | 1.4 | 5.0 | 2.1 | 3.1 | 8.6 |
| Labrador cross | 15 | 4.9 | 1.1 | 4.8 | 1.6 | 3.0 | 6.4 |
| Lhasa Apso | 17 | 6.1 | 1.5 | 6.4 | 1.7 | 3.5 | 9.0 |
| Poodle | 18 | 4.7 | 1.1 | 4.7 | 1.5 | 3.1 | 7.5 |
| Standard Poodle | 11 | 4.7 | 0.9 | 4.7 | 1.1 | 3.3 | 6.0 |

§ Unit of measurement: mmol/L; SD = standard deviation; IQR = interquartile range; Min. = minimum value recorded; Max. = maximum value recorded.

**Table J in S1 File. Descriptive statistics – creatinine^§^**

| **Breed** | **N** | **Mean** | **SD** | **Median** | **IQR** | **Min.** | **Max.** |
| --- | --- | --- | --- | --- | --- | --- | --- |
| Mixed breed | 242 | 96 | 15 | 97 | 19 | 54 | 122 |
|  |  |  |  |  |  |  |  |
| **Ancient** |  |  |  |  |  |  |  |
| Maltese Terrier | 16 | 80 | 14 | 79 | 17 | 59 | 106 |
| Shar Pei | 19 | 101 | 12 | 101 | 14 | 79 | 118 |
| Siberian Husky | 12 | 92 | 12 | 94 | 21 | 75 | 106 |
| Tibetan Terrier | 17 | 101 | 14 | 105 | 12 | 73 | 122 |
|  |  |  |  |  |  |  |  |
| **Toy** |  |  |  |  |  |  |  |
| Chihuahua | 14 | 67 | 11 | 63 | 10 | 53 | 87 |
| Pug | 28 | 89 | 14 | 89 | 20 | 58 | 121 |
| Shih Tzu | 29 | 88 | 12 | 90 | 12 | 55 | 108 |
|  |  |  |  |  |  |  |  |
| **Working** |  |  |  |  |  |  |  |
| Dobermann | 77 | 97 | 12 | 97 | 19 | 59 | 118 |
| German Shepherd Dog | 160 | 105 | 12 | 107 | 19 | 70 | 122 |
| Miniature Schnauzer | 24 | 85 | 12 | 85 | 11 | 57 | 110 |
| Schnauzer | 13 | 88 | 14 | 85 | 21 | 66 | 112 |
|  |  |  |  |  |  |  |  |
| **Sight hound** |  |  |  |  |  |  |  |
| Greyhound | 14 | 107 | 14 | 112 | 11 | 70 | 121 |
| Lurcher | 19 | 101 | 12 | 98 | 19 | 82 | 121 |
|  |  |  |  |  |  |  |  |
| **Mastiff-like** |  |  |  |  |  |  |  |
| Boston Terrier | 15 | 98 | 14 | 96 | 13 | 64 | 118 |
| Boxer | 146 | 105 | 11 | 106 | 19 | 78 | 122 |
| Bullmastiff | 23 | 104 | 11 | 106 | 16 | 82 | 118 |
| Bulldog | 39 | 95 | 14 | 98 | 17 | 56 | 122 |
| Dogue de Bordeaux | 13 | 105 | 11 | 108 | 19 | 85 | 122 |
| French Bulldog | 20 | 93 | 11 | 93 | 22 | 76 | 110 |
| Mastiff | 13 | 110 | 6 | 111 | 5 | 101 | 120 |
| Staffordshire Bull Terrier | 95 | 99 | 12 | 102 | 18 | 66 | 121 |
|  |  |  |  |  |  |  |  |
| **Retriever/other Mastiff-like** |  |  |  |  |  |  |  |
| Flat Coated Retriever | 12 | 103 | 10 | 102 | 8 | 86 | 120 |
| Golden Retriever | 121 | 96 | 13 | 97 | 20 | 65 | 120 |
| Great Dane | 25 | 106 | 12 | 107 | 10 | 78 | 119 |
| Labrador Retriever | 327 | 102 | 13 | 104 | 20 | 57 | 122 |
| Leonberger | 15 | 109 | 9 | 113 | 17 | 96 | 122 |
| Newfoundland | 29 | 102 | 14 | 105 | 24 | 67 | 121 |
| Retriever | 36 | 101 | 12 | 101 | 12 | 66 | 121 |
| Rottweiler | 49 | 103 | 13 | 102 | 20 | 74 | 121 |
|  |  |  |  |  |  |  |  |
| **Herding** |  |  |  |  |  |  |  |
| Border Collie | 73 | 99 | 12 | 100 | 17 | 71 | 122 |
| Collie | 14 | 103 | 18 | 113 | 24 | 65 | 122 |
| Shetland Sheepdog | 11 | 98 | 12 | 96 | 12 | 77 | 118 |
|  |  |  |  |  |  |  |  |
| **Terrier** |  |  |  |  |  |  |  |
| Airedale | 10 | 100 | 11 | 99 | 8 | 84 | 119 |
| Border Terrier | 35 | 93 | 13 | 95 | 15 | 65 | 119 |
| Norfolk Terrier | 12 | 96 | 9 | 98 | 10 | 81 | 112 |
| West Highland White Terrier | 78 | 84 | 15 | 84 | 19 | 53 | 121 |
| Yorkshire Terrier | 41 | 86 | 16 | 83 | 19 | 55 | 119 |
|  |  |  |  |  |  |  |  |
| **Scent hound** |  |  |  |  |  |  |  |
| Basset Hound | 21 | 81 | 16 | 77 | 22 | 58 | 122 |
| Beagle | 29 | 86 | 17 | 84 | 29 | 61 | 120 |
| Dachshund | 34 | 73 | 15 | 70 | 22 | 52 | 107 |
| Rhodesian Ridgeback | 32 | 98 | 13 | 103 | 16 | 70 | 118 |
|  |  |  |  |  |  |  |  |
| **Spaniel/Pointer** |  |  |  |  |  |  |  |
| Cavalier King Charles Spaniel | 174 | 83 | 14 | 83 | 21 | 56 | 117 |
| Cocker Spaniel | 98 | 80 | 13 | 80 | 17 | 54 | 120 |
| English Springer Spaniel | 41 | 93 | 13 | 94 | 18 | 64 | 122 |
| German Shorthaired Pointer | 19 | 97 | 13 | 99 | 14 | 68 | 119 |
| Gordon Setter | 14 | 86 | 15 | 88 | 21 | 56 | 109 |
| Hungarian Vizsla | 15 | 89 | 12 | 90 | 20 | 72 | 115 |
| Irish Setter | 23 | 86 | 9 | 87 | 16 | 71 | 100 |
| Italian Spinone | 21 | 105 | 11 | 106 | 11 | 68 | 118 |
| Pointer | 13 | 93 | 15 | 95 | 21 | 57 | 111 |
| Springer Spaniel | 71 | 90 | 14 | 90 | 19 | 52 | 121 |
| Weimaraner | 39 | 96 | 15 | 96 | 18 | 53 | 119 |
|  |  |  |  |  |  |  |  |
| **Other** |  |  |  |  |  |  |  |
| Bichon Frise | 14 | 79 | 10 | 78 | 9 | 62 | 97 |
| Collie cross | 16 | 92 | 15 | 93 | 19 | 53 | 108 |
| Dalmatian | 21 | 101 | 13 | 105 | 23 | 77 | 120 |
| Jack Russell Terrier | 78 | 81 | 12 | 80 | 16 | 54 | 115 |
| Labrador cross | 15 | 99 | 16 | 98 | 29 | 76 | 122 |
| Lhasa Apso | 17 | 88 | 17 | 89 | 18 | 52 | 110 |
| Poodle | 18 | 97 | 13 | 100 | 20 | 68 | 113 |
| Standard Poodle | 11 | 101 | 10 | 104 | 10 | 80 | 115 |

§ Unit of measurement: µmol/L; SD = standard deviation; IQR = interquartile range; Min. = minimum value recorded; Max. = maximum value recorded.

**Table K in S1 File. Descriptive statistics – cholesterol^§^**

| **Breed** | **N** | **Mean** | **SD** | **Median** | **IQR** | **Min.** | **Max.** |
| --- | --- | --- | --- | --- | --- | --- | --- |
| Mixed breed | 242 | 6.0 | 1.3 | 5.9 | 2.1 | 3.4 | 8.9 |
|  |  |  |  |  |  |  |  |
| **Ancient** |  |  |  |  |  |  |  |
| Maltese Terrier | 16 | 5.1 | 1.4 | 5.2 | 1.7 | 3.5 | 8.9 |
| Shar Pei | 19 | 5.1 | 1.0 | 4.9 | 1.4 | 3.9 | 7.2 |
| Siberian Husky | 12 | 6.2 | 1.2 | 6.6 | 1.5 | 4.3 | 8.2 |
| Tibetan Terrier | 17 | 4.9 | 0.9 | 4.9 | 1.2 | 3.6 | 6.9 |
|  |  |  |  |  |  |  |  |
| **Toy** |  |  |  |  |  |  |  |
| Chihuahua | 14 | 5.0 | 0.7 | 5.1 | 1.1 | 3.6 | 6.1 |
| Pug | 28 | 6.8 | 1.2 | 6.8 | 1.8 | 5.0 | 8.8 |
| Shih Tzu | 29 | 6.6 | 1.6 | 6.8 | 2.8 | 3.5 | 8.9 |
|  |  |  |  |  |  |  |  |
| **Working** |  |  |  |  |  |  |  |
| Dobermann | 77 | 5.8 | 1.2 | 5.7 | 1.8 | 3.5 | 8.9 |
| German Shepherd Dog | 160 | 5.6 | 1.2 | 5.5 | 1.5 | 3.3 | 8.6 |
| Miniature Schnauzer | 24 | 6.1 | 1.4 | 6.0 | 1.9 | 3.6 | 8.8 |
| Schnauzer | 13 | 5.7 | 1.8 | 5.3 | 3.3 | 3.6 | 8.6 |
|  |  |  |  |  |  |  |  |
| **Sight hound** |  |  |  |  |  |  |  |
| Greyhound | 14 | 5.4 | 1.1 | 5.2 | 0.9 | 3.8 | 7.6 |
| Lurcher | 19 | 5.3 | 1.3 | 5.1 | 2.1 | 3.9 | 8.1 |
|  |  |  |  |  |  |  |  |
| **Mastiff-like** |  |  |  |  |  |  |  |
| Boston Terrier | 15 | 5.0 | 0.7 | 4.9 | 1.2 | 3.8 | 5.9 |
| Boxer | 146 | 6.5 | 1.2 | 6.3 | 1.8 | 3.8 | 8.9 |
| Bullmastiff | 23 | 7.1 | 1.0 | 7.2 | 1.2 | 5.1 | 8.8 |
| Bulldog | 39 | 6.3 | 1.0 | 6.5 | 1.4 | 4.3 | 8.4 |
| Dogue de Bordeaux | 13 | 7.6 | 0.9 | 7.7 | 1.3 | 6.1 | 8.9 |
| French Bulldog | 20 | 5.6 | 1.1 | 5.4 | 1.3 | 3.8 | 7.9 |
| Mastiff | 13 | 5.8 | 1.2 | 5.5 | 1.4 | 4.5 | 8.8 |
| Staffordshire Bull Terrier | 95 | 6.4 | 1.0 | 6.3 | 1.1 | 4.2 | 8.9 |
|  |  |  |  |  |  |  |  |
| **Retriever/other Mastiff-like** |  |  |  |  |  |  |  |
| Flat Coated Retriever | 12 | 6.1 | 1.5 | 5.8 | 2.4 | 4.0 | 8.3 |
| Golden Retriever | 121 | 6.8 | 1.3 | 7.0 | 1.9 | 3.3 | 8.9 |
| Great Dane | 25 | 5.9 | 1.4 | 6.3 | 2.5 | 3.9 | 8.9 |
| Labrador Retriever | 327 | 5.5 | 1.2 | 5.4 | 1.8 | 3.4 | 8.8 |
| Leonberger | 15 | 6.6 | 1.1 | 6.8 | 1.1 | 4.4 | 8.6 |
| Newfoundland | 29 | 5.7 | 1.4 | 5.5 | 1.8 | 3.9 | 8.9 |
| Retriever | 36 | 6.7 | 1.2 | 6.8 | 1.4 | 3.7 | 8.9 |
| Rottweiler | 49 | 7.0 | 1.1 | 6.8 | 1.7 | 4.3 | 8.9 |
|  |  |  |  |  |  |  |  |
| **Herding** |  |  |  |  |  |  |  |
| Border Collie | 73 | 6.2 | 1.3 | 6.3 | 1.9 | 3.6 | 8.9 |
| Collie | 14 | 6.5 | 1.0 | 6.4 | 1.2 | 4.9 | 8.3 |
| Shetland Sheepdog | 11 | 6.2 | 1.5 | 6.5 | 1.7 | 3.7 | 8.5 |
|  |  |  |  |  |  |  |  |
| **Terrier** |  |  |  |  |  |  |  |
| Airedale | 10 | 6.5 | 0.8 | 6.4 | 0.5 | 5.3 | 8.0 |
| Border Terrier | 35 | 6.0 | 1.4 | 5.9 | 2.7 | 3.5 | 8.6 |
| Norfolk Terrier | 12 | 5.3 | 0.9 | 5.2 | 1.0 | 3.7 | 7.2 |
| West Highland White Terrier | 78 | 5.9 | 1.4 | 5.8 | 2.0 | 3.4 | 8.9 |
| Yorkshire Terrier | 41 | 5.2 | 1.3 | 4.9 | 1.4 | 3.3 | 8.4 |
|  |  |  |  |  |  |  |  |
| **Scent hound** |  |  |  |  |  |  |  |
| Basset Hound | 21 | 5.7 | 0.9 | 5.8 | 1.4 | 3.9 | 6.8 |
| Beagle | 29 | 5.5 | 1.0 | 5.3 | 1.2 | 3.6 | 8.1 |
| Dachshund | 34 | 5.2 | 1.1 | 5.1 | 1.9 | 3.4 | 7.4 |
| Rhodesian Ridgeback | 32 | 5.8 | 1.3 | 5.6 | 1.7 | 3.7 | 8.9 |
|  |  |  |  |  |  |  |  |
| **Spaniel/Pointer** |  |  |  |  |  |  |  |
| Cavalier King Charles Spaniel | 174 | 6.4 | 1.3 | 6.4 | 1.8 | 3.4 | 8.9 |
| Cocker Spaniel | 98 | 5.9 | 1.3 | 5.6 | 1.8 | 3.5 | 8.7 |
| English Springer Spaniel | 41 | 5.7 | 1.2 | 5.6 | 1.7 | 3.5 | 8.7 |
| German Shorthaired Pointer | 19 | 5.6 | 1.2 | 5.5 | 1.7 | 3.6 | 7.7 |
| Gordon Setter | 14 | 5.4 | 1.2 | 5.0 | 1.7 | 4.1 | 7.7 |
| Hungarian Vizsla | 15 | 5.4 | 1.2 | 5.0 | 1.1 | 4.3 | 8.2 |
| Irish Setter | 23 | 6.1 | 1.4 | 6.4 | 2.3 | 3.6 | 8.4 |
| Italian Spinone | 21 | 5.7 | 1.4 | 5.5 | 2.0 | 3.3 | 8.6 |
| Pointer | 13 | 6.6 | 1.1 | 6.4 | 1.8 | 5.2 | 8.2 |
| Springer Spaniel | 71 | 6.0 | 1.2 | 6.0 | 1.8 | 3.6 | 8.9 |
| Weimaraner | 39 | 6.1 | 1.4 | 6.3 | 2.3 | 3.5 | 8.9 |
|  |  |  |  |  |  |  |  |
| **Other** |  |  |  |  |  |  |  |
| Bichon Frise | 14 | 6.1 | 1.4 | 6.3 | 2.0 | 4.1 | 8.7 |
| Collie cross | 16 | 6.0 | 1.4 | 5.5 | 1.8 | 4.7 | 8.6 |
| Dalmatian | 21 | 6.0 | 1.1 | 6.0 | 1.4 | 4.0 | 7.8 |
| Jack Russell Terrier | 78 | 5.6 | 1.2 | 5.5 | 1.4 | 3.4 | 8.9 |
| Labrador cross | 15 | 5.7 | 1.0 | 5.5 | 0.9 | 4.1 | 8.1 |
| Lhasa Apso | 17 | 5.6 | 1.1 | 5.5 | 1.4 | 3.8 | 8.4 |
| Poodle | 18 | 6.2 | 1.2 | 6.0 | 1.5 | 3.4 | 7.7 |
| Standard Poodle | 11 | 6.1 | 1.5 | 5.7 | 1.7 | 3.9 | 8.9 |

§ Unit of measurement: mmol/L; SD = standard deviation; IQR = interquartile range; Min. = minimum value recorded; Max. = maximum value recorded.

**Table L in S1 File. Descriptive statistics – total bilirubin^§^**

| **Breed** | **N** | **Mean** | **SD** | **Median** | **IQR** | **Min.** | **Max.** |
| --- | --- | --- | --- | --- | --- | --- | --- |
| Mixed breed | 242 | 1.2 | 0.6 | 1.3 | 0.9 | 0.0 | 2.4 |
|  |  |  |  |  |  |  |  |
| **Ancient** |  |  |  |  |  |  |  |
| Maltese Terrier | 16 | 0.7 | 0.5 | 0.8 | 0.8 | 0.0 | 1.6 |
| Shar Pei | 19 | 1.2 | 0.6 | 1.2 | 0.7 | 0.0 | 2.0 |
| Siberian Husky | 12 | 1.4 | 0.7 | 1.4 | 0.7 | 0.0 | 2.3 |
| Tibetan Terrier | 17 | 1.1 | 0.6 | 1.0 | 0.3 | 0.0 | 2.2 |
|  |  |  |  |  |  |  |  |
| **Toy** |  |  |  |  |  |  |  |
| Chihuahua | 14 | 0.7 | 0.5 | 0.8 | 0.8 | 0.0 | 1.5 |
| Pug | 28 | 0.9 | 0.7 | 0.9 | 1.1 | 0.0 | 2.2 |
| Shih Tzu | 29 | 1.0 | 0.6 | 1.0 | 0.7 | 0.0 | 2.3 |
|  |  |  |  |  |  |  |  |
| **Working** |  |  |  |  |  |  |  |
| Dobermann | 77 | 1.2 | 0.6 | 1.2 | 0.8 | 0.1 | 2.4 |
| German Shepherd Dog | 160 | 1.1 | 0.6 | 1.1 | 0.8 | 0.0 | 2.4 |
| Miniature Schnauzer | 24 | 1.1 | 0.7 | 1.2 | 1.1 | 0.1 | 2.3 |
| Schnauzer | 13 | 0.7 | 0.6 | 0.5 | 0.9 | 0.0 | 2.1 |
|  |  |  |  |  |  |  |  |
| **Sight hound** |  |  |  |  |  |  |  |
| Greyhound | 14 | 1.4 | 0.8 | 1.7 | 1.0 | 0.0 | 2.4 |
| Lurcher | 19 | 1.4 | 0.6 | 1.5 | 0.9 | 0.4 | 2.3 |
|  |  |  |  |  |  |  |  |
| **Mastiff-like** |  |  |  |  |  |  |  |
| Boston Terrier | 15 | 0.5 | 0.6 | 0.2 | 0.8 | 0.0 | 1.8 |
| Boxer | 146 | 1.1 | 0.7 | 1.1 | 1.0 | 0.0 | 2.4 |
| Bullmastiff | 23 | 1.3 | 0.7 | 1.3 | 1.1 | 0.0 | 2.4 |
| Bulldog | 39 | 0.9 | 0.7 | 1.0 | 1.2 | 0.0 | 2.3 |
| Dogue de Bordeaux | 13 | 1.2 | 0.5 | 1.2 | 0.4 | 0.0 | 2.1 |
| French Bulldog | 20 | 0.9 | 0.7 | 0.7 | 0.9 | 0.0 | 2.0 |
| Mastiff | 13 | 1.3 | 0.7 | 1.4 | 0.4 | 0.0 | 2.4 |
| Staffordshire Bull Terrier | 95 | 1.1 | 0.7 | 1.1 | 1.0 | 0.0 | 2.4 |
|  |  |  |  |  |  |  |  |
| **Retriever/other Mastiff-like** |  |  |  |  |  |  |  |
| Flat Coated Retriever | 12 | 1.1 | 0.5 | 1.1 | 0.5 | 0.0 | 1.9 |
| Golden Retriever | 121 | 1.3 | 0.6 | 1.3 | 0.9 | 0.0 | 2.4 |
| Great Dane | 25 | 1.7 | 0.5 | 1.7 | 0.6 | 0.7 | 2.4 |
| Labrador Retriever | 327 | 1.3 | 0.6 | 1.4 | 0.9 | 0.0 | 2.4 |
| Leonberger | 15 | 1.3 | 0.7 | 1.4 | 1.2 | 0.0 | 2.4 |
| Newfoundland | 29 | 1.3 | 0.6 | 1.2 | 0.8 | 0.0 | 2.4 |
| Retriever | 36 | 1.3 | 0.6 | 1.5 | 0.7 | 0.0 | 2.3 |
| Rottweiler | 49 | 1.4 | 0.7 | 1.5 | 1.2 | 0.0 | 2.4 |
|  |  |  |  |  |  |  |  |
| **Herding** |  |  |  |  |  |  |  |
| Border Collie | 73 | 1.2 | 0.6 | 1.2 | 0.8 | 0.0 | 2.4 |
| Collie | 14 | 1.2 | 0.7 | 1.3 | 1.1 | 0.0 | 2.3 |
| Shetland Sheepdog | 11 | 0.5 | 0.7 | 0.2 | 0.5 | 0.0 | 2.3 |
|  |  |  |  |  |  |  |  |
| **Terrier** |  |  |  |  |  |  |  |
| Airedale | 10 | 0.9 | 0.5 | 0.9 | 0.6 | 0.1 | 1.6 |
| Border Terrier | 35 | 1.0 | 0.6 | 1.0 | 0.8 | 0.0 | 2.2 |
| Norfolk Terrier | 12 | 0.9 | 0.6 | 1.0 | 0.9 | 0.0 | 1.8 |
| West Highland White Terrier | 78 | 0.8 | 0.6 | 0.9 | 1.0 | 0.0 | 2.3 |
| Yorkshire Terrier | 41 | 0.9 | 0.7 | 0.9 | 0.9 | 0.0 | 2.4 |
|  |  |  |  |  |  |  |  |
| **Scent hound** |  |  |  |  |  |  |  |
| Basset Hound | 21 | 1.0 | 0.7 | 0.8 | 1.2 | 0.0 | 2.2 |
| Beagle | 29 | 1.1 | 0.6 | 1.0 | 0.7 | 0.0 | 2.4 |
| Dachshund | 34 | 0.7 | 0.5 | 0.7 | 0.8 | 0.0 | 2.2 |
| Rhodesian Ridgeback | 32 | 1.2 | 0.7 | 1.2 | 1.1 | 0.0 | 2.4 |
|  |  |  |  |  |  |  |  |
| **Spaniel/Pointer** |  |  |  |  |  |  |  |
| Cavalier King Charles Spaniel | 174 | 0.8 | 0.6 | 0.8 | 0.8 | 0.0 | 2.4 |
| Cocker Spaniel | 98 | 1.0 | 0.7 | 1.1 | 1.3 | 0.0 | 2.4 |
| English Springer Spaniel | 41 | 1.1 | 0.6 | 1.1 | 0.7 | 0.0 | 2.3 |
| German Shorthaired Pointer | 19 | 1.2 | 0.7 | 1.1 | 1.0 | 0.0 | 2.4 |
| Gordon Setter | 14 | 1.2 | 0.6 | 1.3 | 0.6 | 0.0 | 2.2 |
| Hungarian Vizsla | 15 | 1.1 | 0.5 | 1.1 | 0.4 | 0.4 | 2.3 |
| Irish Setter | 23 | 1.0 | 0.5 | 1.0 | 0.7 | 0.0 | 2.0 |
| Italian Spinone | 21 | 1.2 | 0.6 | 1.1 | 0.7 | 0.3 | 2.4 |
| Pointer | 13 | 1.1 | 0.8 | 1.0 | 1.5 | 0.0 | 2.3 |
| Springer Spaniel | 71 | 1.2 | 0.7 | 1.1 | 1.1 | 0.0 | 2.4 |
| Weimaraner | 39 | 1.2 | 0.5 | 1.1 | 0.7 | 0.0 | 2.2 |
|  |  |  |  |  |  |  |  |
| **Other** |  |  |  |  |  |  |  |
| Bichon Frise | 14 | 0.8 | 0.7 | 0.7 | 1.2 | 0.0 | 2.2 |
| Collie cross | 16 | 1.0 | 0.7 | 1.0 | 0.9 | 0.0 | 2.4 |
| Dalmatian | 21 | 1.3 | 0.5 | 1.1 | 0.6 | 0.5 | 2.3 |
| Jack Russell Terrier | 78 | 0.9 | 0.6 | 0.9 | 1.0 | 0.0 | 2.4 |
| Labrador cross | 15 | 1.5 | 0.5 | 1.4 | 0.6 | 0.6 | 2.4 |
| Lhasa Apso | 17 | 1.0 | 0.7 | 0.9 | 1.1 | 0.0 | 2.2 |
| Poodle | 18 | 1.4 | 0.5 | 1.4 | 0.7 | 0.4 | 2.4 |
| Standard Poodle | 11 | 1.3 | 0.6 | 1.5 | 0.8 | 0.1 | 2.2 |

§ Unit of measurement: µmol/L; SD = standard deviation; IQR = interquartile range; Min. = minimum value recorded; Max. = maximum value recorded.

**Table M in S1 File. Descriptive statistics – ALT^§^**

| **Breed** | **N** | **Mean** | **SD** | **Median** | **IQR** | **Min.** | **Max.** |
| --- | --- | --- | --- | --- | --- | --- | --- |
| Mixed breed | 242 | 41 | 17 | 38 | 22 | 13 | 88 |
|  |  |  |  |  |  |  |  |
| **Ancient** |  |  |  |  |  |  |  |
| Maltese Terrier | 16 | 51 | 17 | 51 | 25 | 29 | 86 |
| Shar Pei | 19 | 32 | 20 | 23 | 16 | 16 | 80 |
| Siberian Husky | 12 | 42 | 19 | 35 | 28 | 17 | 76 |
| Tibetan Terrier | 17 | 41 | 25 | 30 | 35 | 13 | 88 |
|  |  |  |  |  |  |  |  |
| **Toy** |  |  |  |  |  |  |  |
| Chihuahua | 14 | 48 | 19 | 44 | 25 | 18 | 75 |
| Pug | 28 | 43 | 15 | 40 | 16 | 24 | 81 |
| Shih Tzu | 29 | 36 | 10 | 37 | 13 | 22 | 60 |
|  |  |  |  |  |  |  |  |
| **Working** |  |  |  |  |  |  |  |
| Dobermann | 77 | 43 | 17 | 38 | 18 | 19 | 88 |
| German Shepherd Dog | 160 | 42 | 16 | 38 | 18 | 17 | 85 |
| Miniature Schnauzer | 24 | 38 | 16 | 36 | 20 | 19 | 72 |
| Schnauzer | 13 | 42 | 18 | 35 | 35 | 19 | 65 |
|  |  |  |  |  |  |  |  |
| **Sight hound** |  |  |  |  |  |  |  |
| Greyhound | 14 | 52 | 19 | 52 | 26 | 20 | 85 |
| Lurcher | 19 | 42 | 14 | 42 | 15 | 24 | 82 |
|  |  |  |  |  |  |  |  |
| **Mastiff-like** |  |  |  |  |  |  |  |
| Boston Terrier | 15 | 40 | 9 | 41 | 15 | 26 | 58 |
| Boxer | 146 | 47 | 14 | 43 | 19 | 19 | 87 |
| Bullmastiff | 23 | 32 | 14 | 27 | 11 | 17 | 66 |
| Bulldog | 39 | 33 | 14 | 29 | 12 | 19 | 69 |
| Dogue de Bordeaux | 13 | 36 | 12 | 31 | 20 | 18 | 56 |
| French Bulldog | 20 | 40 | 12 | 38 | 15 | 20 | 63 |
| Mastiff | 13 | 48 | 19 | 42 | 16 | 18 | 84 |
| Staffordshire Bull Terrier | 95 | 43 | 17 | 39 | 21 | 14 | 85 |
|  |  |  |  |  |  |  |  |
| **Retriever/other Mastiff-like** |  |  |  |  |  |  |  |
| Flat Coated Retriever | 12 | 43 | 10 | 40 | 10 | 30 | 61 |
| Golden Retriever | 121 | 38 | 14 | 34 | 17 | 17 | 86 |
| Great Dane | 25 | 38 | 13 | 37 | 11 | 16 | 64 |
| Labrador Retriever | 327 | 43 | 15 | 41 | 17 | 13 | 88 |
| Leonberger | 15 | 39 | 14 | 35 | 9 | 27 | 83 |
| Newfoundland | 29 | 34 | 16 | 28 | 15 | 13 | 72 |
| Retriever | 36 | 37 | 13 | 36 | 14 | 22 | 85 |
| Rottweiler | 49 | 32 | 11 | 31 | 14 | 16 | 64 |
|  |  |  |  |  |  |  |  |
| **Herding** |  |  |  |  |  |  |  |
| Border Collie | 73 | 38 | 15 | 34 | 21 | 17 | 76 |
| Collie | 14 | 38 | 19 | 34 | 24 | 17 | 86 |
| Shetland Sheepdog | 11 | 41 | 10 | 37 | 14 | 25 | 57 |
|  |  |  |  |  |  |  |  |
| **Terrier** |  |  |  |  |  |  |  |
| Airedale | 10 | 30 | 10 | 29 | 14 | 18 | 50 |
| Border Terrier | 35 | 28 | 13 | 25 | 19 | 13 | 73 |
| Norfolk Terrier | 12 | 45 | 20 | 35 | 27 | 28 | 85 |
| West Highland White Terrier | 78 | 34 | 15 | 31 | 17 | 14 | 88 |
| Yorkshire Terrier | 41 | 40 | 14 | 40 | 23 | 17 | 69 |
|  |  |  |  |  |  |  |  |
| **Scent hound** |  |  |  |  |  |  |  |
| Basset Hound | 21 | 27 | 14 | 24 | 14 | 13 | 63 |
| Beagle | 29 | 33 | 12 | 28 | 15 | 20 | 59 |
| Dachshund | 34 | 45 | 19 | 39 | 25 | 17 | 87 |
| Rhodesian Ridgeback | 32 | 32 | 11 | 31 | 15 | 17 | 59 |
|  |  |  |  |  |  |  |  |
| **Spaniel/Pointer** |  |  |  |  |  |  |  |
| Cavalier King Charles Spaniel | 174 | 29 | 12 | 27 | 12 | 13 | 81 |
| Cocker Spaniel | 98 | 36 | 16 | 32 | 18 | 15 | 81 |
| English Springer Spaniel | 41 | 35 | 17 | 29 | 17 | 17 | 84 |
| German Shorthaired Pointer | 19 | 48 | 15 | 45 | 24 | 24 | 76 |
| Gordon Setter | 14 | 40 | 21 | 34 | 4 | 17 | 84 |
| Hungarian Vizsla | 15 | 41 | 14 | 40 | 8 | 22 | 71 |
| Irish Setter | 23 | 40 | 12 | 38 | 18 | 24 | 66 |
| Italian Spinone | 21 | 41 | 17 | 35 | 19 | 22 | 80 |
| Pointer | 13 | 37 | 14 | 37 | 11 | 15 | 65 |
| Springer Spaniel | 71 | 33 | 12 | 31 | 13 | 13 | 72 |
| Weimaraner | 39 | 43 | 19 | 35 | 27 | 18 | 86 |
|  |  |  |  |  |  |  |  |
| **Other** |  |  |  |  |  |  |  |
| Bichon Frise | 14 | 44 | 19 | 37 | 11 | 18 | 80 |
| Collie cross | 16 | 31 | 8 | 32 | 13 | 19 | 44 |
| Dalmatian | 21 | 32 | 11 | 28 | 14 | 17 | 56 |
| Jack Russell Terrier | 78 | 42 | 16 | 38 | 20 | 15 | 86 |
| Labrador cross | 15 | 40 | 11 | 39 | 21 | 24 | 59 |
| Lhasa Apso | 17 | 33 | 15 | 28 | 12 | 20 | 78 |
| Poodle | 18 | 44 | 16 | 39 | 20 | 25 | 81 |
| Standard Poodle | 11 | 47 | 21 | 42 | 34 | 20 | 78 |

§ Unit of measurement: U/L; SD = standard deviation; IQR = interquartile range; Min. = minimum value recorded; Max. = maximum value recorded.

**Table N in S1 File. Descriptive statistics – ALP^§^**

| **Breed** | **N** | **Mean** | **SD** | **Median** | **IQR** | **Min.** | **Max.** |
| --- | --- | --- | --- | --- | --- | --- | --- |
| Mixed breed | 242 | 75 | 57 | 56 | 53 | 19 | 269 |
|  |  |  |  |  |  |  |  |
| **Ancient** |  |  |  |  |  |  |  |
| Maltese Terrier | 16 | 65 | 48 | 46 | 56 | 22 | 172 |
| Shar Pei | 19 | 71 | 63 | 36 | 81 | 19 | 222 |
| Siberian Husky | 12 | 62 | 36 | 45 | 54 | 26 | 138 |
| Tibetan Terrier | 17 | 114 | 81 | 103 | 141 | 26 | 271 |
|  |  |  |  |  |  |  |  |
| **Toy** |  |  |  |  |  |  |  |
| Chihuahua | 14 | 51 | 39 | 38 | 27 | 19 | 140 |
| Pug | 28 | 41 | 21 | 36 | 19 | 21 | 118 |
| Shih Tzu | 29 | 50 | 33 | 33 | 33 | 20 | 142 |
|  |  |  |  |  |  |  |  |
| **Working** |  |  |  |  |  |  |  |
| Dobermann | 77 | 77 | 53 | 61 | 50 | 22 | 281 |
| German Shepherd Dog | 160 | 47 | 36 | 34 | 33 | 19 | 271 |
| Miniature Schnauzer | 24 | 83 | 63 | 65 | 60 | 23 | 249 |
| Schnauzer | 13 | 75 | 68 | 49 | 35 | 19 | 251 |
|  |  |  |  |  |  |  |  |
| **Sight hound** |  |  |  |  |  |  |  |
| Greyhound | 14 | 56 | 32 | 46 | 39 | 19 | 129 |
| Lurcher | 19 | 55 | 25 | 52 | 37 | 23 | 114 |
|  |  |  |  |  |  |  |  |
| **Mastiff-like** |  |  |  |  |  |  |  |
| Boston Terrier | 15 | 69 | 29 | 57 | 42 | 27 | 123 |
| Boxer | 146 | 62 | 42 | 51 | 47 | 19 | 277 |
| Bullmastiff | 23 | 83 | 47 | 63 | 56 | 21 | 205 |
| Bulldog | 39 | 67 | 43 | 57 | 34 | 21 | 226 |
| Dogue de Bordeaux | 13 | 49 | 19 | 41 | 27 | 21 | 87 |
| French Bulldog | 20 | 49 | 45 | 32 | 18 | 19 | 220 |
| Mastiff | 13 | 66 | 49 | 54 | 18 | 20 | 201 |
| Staffordshire Bull Terrier | 95 | 80 | 61 | 55 | 61 | 19 | 280 |
|  |  |  |  |  |  |  |  |
| **Retriever/other Mastiff-like** |  |  |  |  |  |  |  |
| Flat Coated Retriever | 12 | 89 | 49 | 76 | 73 | 30 | 194 |
| Golden Retriever | 121 | 60 | 33 | 53 | 31 | 19 | 214 |
| Great Dane | 25 | 83 | 35 | 77 | 46 | 33 | 166 |
| Labrador Retriever | 327 | 57 | 47 | 41 | 37 | 19 | 278 |
| Leonberger | 15 | 68 | 31 | 70 | 52 | 25 | 119 |
| Newfoundland | 29 | 55 | 38 | 44 | 22 | 21 | 215 |
| Retriever | 36 | 72 | 52 | 55 | 40 | 25 | 259 |
| Rottweiler | 49 | 85 | 49 | 73 | 42 | 24 | 276 |
|  |  |  |  |  |  |  |  |
| **Herding** |  |  |  |  |  |  |  |
| Border Collie | 73 | 65 | 41 | 52 | 50 | 19 | 202 |
| Collie | 14 | 75 | 60 | 55 | 36 | 28 | 211 |
| Shetland Sheepdog | 11 | 66 | 74 | 40 | 44 | 22 | 282 |
|  |  |  |  |  |  |  |  |
| **Terrier** |  |  |  |  |  |  |  |
| Airedale | 10 | 64 | 31 | 49 | 55 | 32 | 111 |
| Border Terrier | 35 | 69 | 46 | 57 | 44 | 21 | 208 |
| Norfolk Terrier | 12 | 86 | 68 | 76 | 68 | 20 | 245 |
| West Highland White Terrier | 78 | 110 | 65 | 100 | 83 | 20 | 283 |
| Yorkshire Terrier | 41 | 51 | 31 | 38 | 37 | 19 | 153 |
|  |  |  |  |  |  |  |  |
| **Scent hound** |  |  |  |  |  |  |  |
| Basset Hound | 21 | 61 | 32 | 50 | 34 | 32 | 177 |
| Beagle | 29 | 90 | 51 | 77 | 68 | 24 | 231 |
| Dachshund | 34 | 55 | 44 | 41 | 37 | 19 | 222 |
| Rhodesian Ridgeback | 32 | 50 | 25 | 44 | 34 | 19 | 135 |
|  |  |  |  |  |  |  |  |
| **Spaniel/Pointer** |  |  |  |  |  |  |  |
| Cavalier King Charles Spaniel | 174 | 59 | 50 | 38 | 30 | 19 | 254 |
| Cocker Spaniel | 98 | 68 | 48 | 51 | 44 | 19 | 261 |
| English Springer Spaniel | 41 | 74 | 52 | 60 | 43 | 22 | 233 |
| German Shorthaired Pointer | 19 | 69 | 55 | 47 | 49 | 23 | 234 |
| Gordon Setter | 14 | 49 | 48 | 32 | 31 | 19 | 204 |
| Hungarian Vizsla | 15 | 65 | 42 | 49 | 33 | 28 | 191 |
| Irish Setter | 23 | 65 | 51 | 40 | 55 | 23 | 199 |
| Italian Spinone | 21 | 73 | 62 | 50 | 63 | 20 | 244 |
| Pointer | 13 | 90 | 65 | 70 | 46 | 27 | 227 |
| Springer Spaniel | 71 | 78 | 51 | 52 | 78 | 20 | 270 |
| Weimaraner | 39 | 90 | 52 | 74 | 82 | 23 | 218 |
|  |  |  |  |  |  |  |  |
| **Other** |  |  |  |  |  |  |  |
| Bichon Frise | 14 | 61 | 47 | 51 | 29 | 19 | 180 |
| Collie cross | 16 | 56 | 25 | 54 | 23 | 19 | 104 |
| Dalmatian | 21 | 80 | 62 | 46 | 66 | 20 | 203 |
| Jack Russell Terrier | 78 | 67 | 49 | 49 | 60 | 20 | 223 |
| Labrador cross | 15 | 54 | 29 | 38 | 39 | 25 | 110 |
| Lhasa Apso | 17 | 54 | 38 | 38 | 37 | 20 | 143 |
| Poodle | 18 | 44 | 21 | 38 | 16 | 20 | 87 |
| Standard Poodle | 11 | 57 | 22 | 58 | 31 | 29 | 104 |

§ Unit of measurement: U/L; SD = standard deviation; IQR = interquartile range; Min. = minimum value recorded; Max. = maximum value recorded.

**Table O in S1 File. Descriptive statistics – CK^§^**

| **Breed** | **N** | **Mean** | **SD** | **Median** | **IQR** | **Min.** | **Max.** |
| --- | --- | --- | --- | --- | --- | --- | --- |
| Mixed breed | 242 | 153 | 76 | 134 | 87 | 61 | 390 |
|  |  |  |  |  |  |  |  |
| **Ancient** |  |  |  |  |  |  |  |
| Maltese Terrier | 16 | 163 | 79 | 136 | 62 | 81 | 350 |
| Shar Pei | 19 | 188 | 67 | 175 | 82 | 78 | 326 |
| Siberian Husky | 12 | 135 | 55 | 124 | 26 | 79 | 258 |
| Tibetan Terrier | 17 | 157 | 62 | 147 | 71 | 61 | 323 |
|  |  |  |  |  |  |  |  |
| **Toy** |  |  |  |  |  |  |  |
| Chihuahua | 14 | 164 | 66 | 153 | 89 | 72 | 267 |
| Pug | 28 | 178 | 65 | 163 | 67 | 90 | 334 |
| Shih Tzu | 29 | 134 | 55 | 122 | 55 | 61 | 279 |
|  |  |  |  |  |  |  |  |
| **Working** |  |  |  |  |  |  |  |
| Dobermann | 77 | 153 | 69 | 132 | 98 | 66 | 359 |
| German Shepherd Dog | 160 | 143 | 70 | 122 | 80 | 62 | 353 |
| Miniature Schnauzer | 24 | 183 | 85 | 172 | 98 | 64 | 388 |
| Schnauzer | 13 | 167 | 87 | 138 | 29 | 79 | 351 |
|  |  |  |  |  |  |  |  |
| **Sight hound** |  |  |  |  |  |  |  |
| Greyhound | 14 | 147 | 56 | 137 | 94 | 67 | 254 |
| Lurcher | 19 | 167 | 81 | 132 | 87 | 75 | 383 |
|  |  |  |  |  |  |  |  |
| **Mastiff-like** |  |  |  |  |  |  |  |
| Boston Terrier | 15 | 174 | 45 | 190 | 66 | 88 | 230 |
| Boxer | 146 | 157 | 74 | 137 | 73 | 69 | 390 |
| Bullmastiff | 23 | 161 | 68 | 137 | 92 | 85 | 372 |
| Bulldog | 39 | 176 | 79 | 150 | 94 | 70 | 394 |
| Dogue de Bordeaux | 13 | 143 | 70 | 127 | 99 | 73 | 302 |
| French Bulldog | 20 | 179 | 57 | 173 | 37 | 87 | 344 |
| Mastiff | 13 | 155 | 44 | 147 | 49 | 94 | 249 |
| Staffordshire Bull Terrier | 95 | 175 | 72 | 168 | 96 | 63 | 389 |
|  |  |  |  |  |  |  |  |
| **Retriever/other Mastiff-like** |  |  |  |  |  |  |  |
| Flat Coated Retriever | 12 | 185 | 73 | 180 | 119 | 94 | 310 |
| Golden Retriever | 121 | 149 | 76 | 122 | 87 | 62 | 390 |
| Great Dane | 25 | 127 | 63 | 106 | 36 | 66 | 305 |
| Labrador Retriever | 327 | 153 | 70 | 137 | 89 | 63 | 394 |
| Leonberger | 15 | 172 | 65 | 155 | 78 | 83 | 278 |
| Newfoundland | 29 | 151 | 52 | 151 | 64 | 73 | 267 |
| Retriever | 36 | 171 | 79 | 144 | 118 | 76 | 361 |
| Rottweiler | 49 | 167 | 60 | 145 | 81 | 95 | 349 |
|  |  |  |  |  |  |  |  |
| **Herding** |  |  |  |  |  |  |  |
| Border Collie | 73 | 141 | 73 | 113 | 93 | 61 | 372 |
| Collie | 14 | 126 | 77 | 94 | 63 | 61 | 324 |
| Shetland Sheepdog | 11 | 170 | 68 | 153 | 49 | 92 | 336 |
|  |  |  |  |  |  |  |  |
| **Terrier** |  |  |  |  |  |  |  |
| Airedale | 10 | 146 | 66 | 130 | 74 | 81 | 297 |
| Border Terrier | 35 | 150 | 57 | 132 | 96 | 69 | 266 |
| Norfolk Terrier | 12 | 124 | 73 | 95 | 52 | 65 | 310 |
| West Highland White Terrier | 78 | 213 | 75 | 201 | 125 | 67 | 382 |
| Yorkshire Terrier | 41 | 173 | 72 | 163 | 96 | 71 | 356 |
|  |  |  |  |  |  |  |  |
| **Scent hound** |  |  |  |  |  |  |  |
| Basset Hound | 21 | 198 | 72 | 193 | 109 | 78 | 328 |
| Beagle | 29 | 158 | 71 | 144 | 82 | 70 | 304 |
| Dachshund | 34 | 186 | 78 | 174 | 123 | 85 | 352 |
| Rhodesian Ridgeback | 32 | 160 | 68 | 151 | 95 | 63 | 318 |
|  |  |  |  |  |  |  |  |
| **Spaniel/Pointer** |  |  |  |  |  |  |  |
| Cavalier King Charles Spaniel | 174 | 173 | 74 | 159 | 98 | 63 | 392 |
| Cocker Spaniel | 98 | 147 | 66 | 133 | 64 | 62 | 370 |
| English Springer Spaniel | 41 | 171 | 80 | 135 | 107 | 67 | 348 |
| German Shorthaired Pointer | 19 | 160 | 80 | 147 | 98 | 66 | 369 |
| Gordon Setter | 14 | 122 | 48 | 108 | 55 | 71 | 228 |
| Hungarian Vizsla | 15 | 141 | 63 | 118 | 61 | 71 | 312 |
| Irish Setter | 23 | 169 | 66 | 156 | 93 | 83 | 318 |
| Italian Spinone | 21 | 130 | 56 | 116 | 49 | 74 | 316 |
| Pointer | 13 | 149 | 80 | 125 | 115 | 66 | 310 |
| Springer Spaniel | 71 | 179 | 86 | 158 | 136 | 74 | 393 |
| Weimaraner | 39 | 170 | 82 | 149 | 116 | 71 | 382 |
|  |  |  |  |  |  |  |  |
| **Other** |  |  |  |  |  |  |  |
| Bichon Frise | 14 | 145 | 57 | 127 | 56 | 84 | 307 |
| Collie cross | 16 | 144 | 58 | 139 | 70 | 77 | 298 |
| Dalmatian | 21 | 152 | 60 | 139 | 45 | 72 | 281 |
| Jack Russell Terrier | 78 | 164 | 71 | 143 | 75 | 64 | 387 |
| Labrador cross | 15 | 162 | 79 | 136 | 79 | 88 | 356 |
| Lhasa Apso | 17 | 140 | 50 | 130 | 65 | 77 | 255 |
| Poodle | 18 | 174 | 85 | 152 | 87 | 69 | 339 |
| Standard Poodle | 11 | 131 | 43 | 127 | 57 | 72 | 214 |

§ Unit of measurement: U/L; SD = standard deviation; IQR = interquartile range; Min. = minimum value recorded; Max. = maximum value recorded.

**Table P in S1 File. Descriptive statistics – amylase^§^**

| **Breed** | **N** | **Mean** | **SD** | **Median** | **IQR** | **Min.** | **Max.** |
| --- | --- | --- | --- | --- | --- | --- | --- |
| Mixed breed | 242 | 700 | 212 | 700 | 314 | 223 | 1234 |
|  |  |  |  |  |  |  |  |
| **Ancient** |  |  |  |  |  |  |  |
| Maltese Terrier | 16 | 603 | 185 | 560 | 251 | 364 | 1007 |
| Shar Pei | 19 | 632 | 145 | 599 | 114 | 377 | 964 |
| Siberian Husky | 12 | 609 | 293 | 550 | 341 | 210 | 1156 |
| Tibetan Terrier | 17 | 684 | 187 | 692 | 221 | 368 | 1027 |
|  |  |  |  |  |  |  |  |
| **Toy** |  |  |  |  |  |  |  |
| Chihuahua | 14 | 541 | 184 | 546 | 241 | 224 | 839 |
| Pug | 28 | 653 | 206 | 643 | 263 | 383 | 1135 |
| Shih Tzu | 29 | 527 | 132 | 507 | 129 | 317 | 746 |
|  |  |  |  |  |  |  |  |
| **Working** |  |  |  |  |  |  |  |
| Dobermann | 77 | 644 | 180 | 603 | 235 | 226 | 1201 |
| German Shepherd Dog | 160 | 732 | 170 | 729 | 207 | 204 | 1146 |
| Miniature Schnauzer | 24 | 674 | 220 | 599 | 268 | 365 | 1156 |
| Schnauzer | 13 | 709 | 203 | 697 | 125 | 272 | 1088 |
|  |  |  |  |  |  |  |  |
| **Sight hound** |  |  |  |  |  |  |  |
| Greyhound | 14 | 680 | 219 | 607 | 308 | 344 | 1175 |
| Lurcher | 19 | 693 | 245 | 682 | 207 | 253 | 1213 |
|  |  |  |  |  |  |  |  |
| **Mastiff-like** |  |  |  |  |  |  |  |
| Boston Terrier | 15 | 555 | 134 | 511 | 157 | 356 | 840 |
| Boxer | 146 | 898 | 179 | 902 | 232 | 338 | 1244 |
| Bullmastiff | 23 | 796 | 226 | 713 | 357 | 435 | 1171 |
| Bulldog | 39 | 886 | 189 | 880 | 229 | 311 | 1229 |
| Dogue de Bordeaux | 13 | 857 | 152 | 867 | 238 | 604 | 1121 |
| French Bulldog | 20 | 649 | 149 | 653 | 155 | 421 | 1024 |
| Mastiff | 13 | 861 | 155 | 861 | 193 | 655 | 1124 |
| Staffordshire Bull Terrier | 95 | 744 | 187 | 729 | 229 | 418 | 1230 |
|  |  |  |  |  |  |  |  |
| **Retriever/other Mastiff-like** |  |  |  |  |  |  |  |
| Flat Coated Retriever | 12 | 918 | 236 | 912 | 310 | 557 | 1220 |
| Golden Retriever | 121 | 615 | 186 | 589 | 191 | 186 | 1236 |
| Great Dane | 25 | 795 | 202 | 826 | 341 | 423 | 1134 |
| Labrador Retriever | 327 | 736 | 212 | 707 | 317 | 301 | 1237 |
| Leonberger | 15 | 832 | 224 | 765 | 407 | 570 | 1152 |
| Newfoundland | 29 | 726 | 209 | 708 | 186 | 364 | 1236 |
| Retriever | 36 | 623 | 198 | 611 | 237 | 205 | 1190 |
| Rottweiler | 49 | 849 | 189 | 816 | 230 | 351 | 1208 |
|  |  |  |  |  |  |  |  |
| **Herding** |  |  |  |  |  |  |  |
| Border Collie | 73 | 688 | 235 | 653 | 390 | 233 | 1214 |
| Collie | 14 | 717 | 160 | 689 | 172 | 412 | 1027 |
| Shetland Sheepdog | 11 | 782 | 237 | 790 | 427 | 400 | 1032 |
|  |  |  |  |  |  |  |  |
| **Terrier** |  |  |  |  |  |  |  |
| Airedale | 10 | 875 | 225 | 831 | 363 | 588 | 1183 |
| Border Terrier | 35 | 636 | 184 | 623 | 214 | 222 | 1111 |
| Norfolk Terrier | 12 | 703 | 162 | 733 | 182 | 376 | 883 |
| West Highland White Terrier | 78 | 562 | 147 | 564 | 216 | 326 | 1055 |
| Yorkshire Terrier | 41 | 614 | 179 | 584 | 223 | 337 | 1131 |
|  |  |  |  |  |  |  |  |
| **Scent hound** |  |  |  |  |  |  |  |
| Basset Hound | 21 | 717 | 176 | 714 | 234 | 433 | 1109 |
| Beagle | 29 | 822 | 213 | 847 | 273 | 367 | 1183 |
| Dachshund | 34 | 600 | 218 | 556 | 264 | 289 | 1169 |
| Rhodesian Ridgeback | 32 | 699 | 197 | 645 | 274 | 409 | 1084 |
|  |  |  |  |  |  |  |  |
| **Spaniel/Pointer** |  |  |  |  |  |  |  |
| Cavalier King Charles Spaniel | 174 | 737 | 184 | 728 | 210 | 216 | 1220 |
| Cocker Spaniel | 98 | 787 | 219 | 762 | 265 | 307 | 1242 |
| English Springer Spaniel | 41 | 656 | 211 | 655 | 279 | 225 | 1237 |
| German Shorthaired Pointer | 19 | 688 | 188 | 654 | 272 | 357 | 1041 |
| Gordon Setter | 14 | 645 | 187 | 672 | 216 | 372 | 966 |
| Hungarian Vizsla | 15 | 865 | 189 | 922 | 229 | 472 | 1192 |
| Irish Setter | 23 | 811 | 193 | 804 | 267 | 431 | 1188 |
| Italian Spinone | 21 | 714 | 187 | 731 | 223 | 288 | 1207 |
| Pointer | 13 | 843 | 231 | 788 | 280 | 411 | 1179 |
| Springer Spaniel | 71 | 706 | 209 | 705 | 254 | 286 | 1203 |
| Weimaraner | 39 | 687 | 212 | 663 | 272 | 260 | 1185 |
|  |  |  |  |  |  |  |  |
| **Other** |  |  |  |  |  |  |  |
| Bichon Frise | 14 | 667 | 259 | 531 | 458 | 380 | 1087 |
| Collie cross | 16 | 719 | 170 | 741 | 242 | 403 | 1054 |
| Dalmatian | 21 | 776 | 189 | 760 | 177 | 436 | 1153 |
| Jack Russell Terrier | 78 | 632 | 219 | 598 | 322 | 235 | 1221 |
| Labrador cross | 15 | 694 | 196 | 656 | 237 | 486 | 1230 |
| Lhasa Apso | 17 | 573 | 123 | 547 | 128 | 423 | 904 |
| Poodle | 18 | 796 | 171 | 783 | 235 | 512 | 1138 |
| Standard Poodle | 11 | 781 | 161 | 785 | 175 | 501 | 1097 |

§ Unit of measurement: U/L; SD = standard deviation; IQR = interquartile range; Min. = minimum value recorded; Max. = maximum value recorded.

**Table Q in S1 File. Descriptive statistics – lipase^§^**

| **Breed** | **N** | **Mean** | **SD** | **Median** | **IQR** | **Min.** | **Max.** |
| --- | --- | --- | --- | --- | --- | --- | --- |
| Mixed breed | 242 | 406 | 197 | 386 | 238 | 85 | 1083 |
|  |  |  |  |  |  |  |  |
| **Ancient** |  |  |  |  |  |  |  |
| Maltese Terrier | 16 | 382 | 190 | 402 | 266 | 110 | 779 |
| Shar Pei | 19 | 301 | 79 | 277 | 75 | 168 | 484 |
| Siberian Husky | 12 | 590 | 380 | 631 | 679 | 152 | 1112 |
| Tibetan Terrier | 17 | 550 | 178 | 489 | 291 | 323 | 909 |
|  |  |  |  |  |  |  |  |
| **Toy** |  |  |  |  |  |  |  |
| Chihuahua | 14 | 277 | 151 | 268 | 98 | 80 | 568 |
| Pug | 28 | 435 | 187 | 420 | 248 | 118 | 883 |
| Shih Tzu | 29 | 282 | 122 | 265 | 157 | 99 | 583 |
|  |  |  |  |  |  |  |  |
| **Working** |  |  |  |  |  |  |  |
| Dobermann | 77 | 438 | 190 | 451 | 256 | 153 | 902 |
| German Shepherd Dog | 160 | 316 | 156 | 308 | 228 | 76 | 885 |
| Miniature Schnauzer | 24 | 274 | 114 | 262 | 133 | 102 | 534 |
| Schnauzer | 13 | 296 | 146 | 241 | 98 | 133 | 621 |
|  |  |  |  |  |  |  |  |
| **Sight hound** |  |  |  |  |  |  |  |
| Greyhound | 14 | 439 | 266 | 395 | 504 | 93 | 785 |
| Lurcher | 19 | 351 | 124 | 339 | 124 | 172 | 719 |
|  |  |  |  |  |  |  |  |
| **Mastiff-like** |  |  |  |  |  |  |  |
| Boston Terrier | 15 | 506 | 191 | 554 | 286 | 187 | 808 |
| Boxer | 146 | 635 | 194 | 617 | 300 | 124 | 1104 |
| Bullmastiff | 23 | 542 | 194 | 554 | 306 | 230 | 998 |
| Bulldog | 39 | 353 | 197 | 358 | 311 | 81 | 769 |
| Dogue de Bordeaux | 13 | 381 | 201 | 374 | 253 | 115 | 774 |
| French Bulldog | 20 | 355 | 194 | 269 | 318 | 98 | 716 |
| Mastiff | 13 | 477 | 245 | 424 | 284 | 195 | 931 |
| Staffordshire Bull Terrier | 95 | 348 | 212 | 293 | 222 | 83 | 1075 |
|  |  |  |  |  |  |  |  |
| **Retriever/other Mastiff-like** |  |  |  |  |  |  |  |
| Flat Coated Retriever | 12 | 322 | 144 | 308 | 113 | 109 | 601 |
| Golden Retriever | 121 | 200 | 109 | 164 | 120 | 78 | 677 |
| Great Dane | 25 | 308 | 160 | 320 | 258 | 80 | 717 |
| Labrador Retriever | 327 | 302 | 144 | 274 | 218 | 72 | 812 |
| Leonberger | 15 | 405 | 201 | 351 | 323 | 97 | 740 |
| Newfoundland | 29 | 313 | 122 | 294 | 154 | 99 | 544 |
| Retriever | 36 | 224 | 98 | 200 | 159 | 78 | 450 |
| Rottweiler | 49 | 391 | 149 | 376 | 184 | 118 | 869 |
|  |  |  |  |  |  |  |  |
| **Herding** |  |  |  |  |  |  |  |
| Border Collie | 73 | 296 | 138 | 278 | 170 | 82 | 834 |
| Collie | 14 | 277 | 121 | 281 | 117 | 120 | 565 |
| Shetland Sheepdog | 11 | 326 | 183 | 301 | 269 | 97 | 637 |
|  |  |  |  |  |  |  |  |
| **Terrier** |  |  |  |  |  |  |  |
| Airedale | 10 | 526 | 143 | 509 | 105 | 306 | 830 |
| Border Terrier | 35 | 348 | 173 | 326 | 253 | 110 | 709 |
| Norfolk Terrier | 12 | 476 | 253 | 424 | 255 | 170 | 1114 |
| West Highland White Terrier | 78 | 313 | 173 | 262 | 169 | 85 | 834 |
| Yorkshire Terrier | 41 | 484 | 200 | 462 | 230 | 150 | 959 |
|  |  |  |  |  |  |  |  |
| **Scent hound** |  |  |  |  |  |  |  |
| Basset Hound | 21 | 276 | 123 | 261 | 92 | 111 | 710 |
| Beagle | 29 | 460 | 261 | 449 | 413 | 115 | 1069 |
| Dachshund | 34 | 347 | 209 | 281 | 279 | 86 | 842 |
| Rhodesian Ridgeback | 32 | 427 | 188 | 394 | 277 | 112 | 892 |
|  |  |  |  |  |  |  |  |
| **Spaniel/Pointer** |  |  |  |  |  |  |  |
| Cavalier King Charles Spaniel | 174 | 339 | 145 | 308 | 198 | 124 | 924 |
| Cocker Spaniel | 98 | 286 | 181 | 217 | 164 | 90 | 926 |
| English Springer Spaniel | 41 | 339 | 189 | 305 | 287 | 106 | 834 |
| German Shorthaired Pointer | 19 | 362 | 171 | 404 | 249 | 96 | 663 |
| Gordon Setter | 14 | 461 | 304 | 300 | 486 | 144 | 1064 |
| Hungarian Vizsla | 15 | 343 | 91 | 326 | 102 | 159 | 558 |
| Irish Setter | 23 | 448 | 179 | 442 | 274 | 171 | 912 |
| Italian Spinone | 21 | 432 | 156 | 428 | 157 | 193 | 806 |
| Pointer | 13 | 361 | 198 | 346 | 316 | 80 | 691 |
| Springer Spaniel | 71 | 273 | 154 | 229 | 206 | 72 | 719 |
| Weimaraner | 39 | 579 | 224 | 584 | 352 | 85 | 944 |
|  |  |  |  |  |  |  |  |
| **Other** |  |  |  |  |  |  |  |
| Bichon Frise | 14 | 487 | 278 | 383 | 331 | 75 | 1074 |
| Collie cross | 16 | 305 | 184 | 264 | 238 | 110 | 746 |
| Dalmatian | 21 | 340 | 152 | 334 | 168 | 116 | 653 |
| Jack Russell Terrier | 78 | 392 | 189 | 355 | 237 | 78 | 909 |
| Labrador cross | 15 | 340 | 176 | 296 | 193 | 121 | 816 |
| Lhasa Apso | 17 | 301 | 122 | 324 | 199 | 79 | 515 |
| Poodle | 18 | 371 | 134 | 355 | 240 | 163 | 606 |
| Standard Poodle | 11 | 438 | 199 | 382 | 223 | 178 | 794 |

§ Unit of measurement: U/L; SD = standard deviation; IQR = interquartile range; Min. = minimum value recorded; Max. = maximum value recorded.

**Table R in S1 File. Descriptive statistics – glucose^§^**

| **Breed** | **N** | **Mean** | **SD** | **Median** | **IQR** | **Min.** | **Max.** |
| --- | --- | --- | --- | --- | --- | --- | --- |
| Mixed breed | 108 | 5.1 | 0.5 | 5.0 | 0.8 | 3.8 | 6.0 |
|  |  |  |  |  |  |  |  |
| **Ancient** |  |  |  |  |  |  |  |
| Shar Pei | 11 | 5.1 | 0.2 | 5.1 | 0.3 | 4.7 | 5.4 |
|  |  |  |  |  |  |  |  |
| **Toy** |  |  |  |  |  |  |  |
| Shih Tzu | 22 | 4.9 | 0.5 | 5.1 | 0.7 | 4.0 | 5.9 |
|  |  |  |  |  |  |  |  |
| **Working** |  |  |  |  |  |  |  |
| Dobermann | 36 | 5.0 | 0.4 | 4.9 | 0.6 | 4.2 | 5.8 |
| German Shepherd Dog | 83 | 5.0 | 0.5 | 5.0 | 0.5 | 3.4 | 5.9 |
|  |  |  |  |  |  |  |  |
| **Mastiff-like** |  |  |  |  |  |  |  |
| Boxer | 60 | 5.2 | 0.5 | 5.3 | 0.7 | 3.9 | 6.0 |
| Bullmastiff | 12 | 5.2 | 0.5 | 5.1 | 0.7 | 4.5 | 6.0 |
| Bulldog | 17 | 5.3 | 0.5 | 5.4 | 0.8 | 4.3 | 5.9 |
| Staffordshire Bull Terrier | 45 | 5.4 | 0.5 | 5.5 | 0.7 | 3.9 | 6.0 |
|  |  |  |  |  |  |  |  |
| **Retriever/other Mastiff-like** |  |  |  |  |  |  |  |
| Golden Retriever | 65 | 5.0 | 0.5 | 5.0 | 0.6 | 3.9 | 6.0 |
| Great Dane | 15 | 5.3 | 0.5 | 5.2 | 0.7 | 4.5 | 5.9 |
| Labrador Retriever | 161 | 5.2 | 0.4 | 5.2 | 0.5 | 3.7 | 6.0 |
| Newfoundland | 13 | 5.1 | 0.5 | 5.2 | 0.6 | 4.3 | 6.0 |
| Retriever | 23 | 4.9 | 0.4 | 4.9 | 0.7 | 4.2 | 5.6 |
| Rottweiler | 36 | 5.2 | 0.5 | 5.2 | 0.7 | 4.2 | 6.0 |
|  |  |  |  |  |  |  |  |
| **Herding** |  |  |  |  |  |  |  |
| Border Collie | 33 | 5.2 | 0.4 | 5.3 | 0.6 | 4.1 | 6.0 |
| Collie | 10 | 5.0 | 0.6 | 5.0 | 0.7 | 4.2 | 5.9 |
|  |  |  |  |  |  |  |  |
| **Terrier** |  |  |  |  |  |  |  |
| Border Terrier | 24 | 5.2 | 0.4 | 5.2 | 0.6 | 4.3 | 5.9 |
| West Highland White Terrier | 36 | 5.2 | 0.5 | 5.1 | 0.6 | 4.0 | 6.0 |
| Yorkshire Terrier | 23 | 5.1 | 0.6 | 5.1 | 0.7 | 3.5 | 6.0 |
|  |  |  |  |  |  |  |  |
| **Scent hound** |  |  |  |  |  |  |  |
| Basset Hound | 13 | 5.1 | 0.3 | 5.0 | 0.3 | 4.7 | 5.6 |
| Beagle | 14 | 5.3 | 0.5 | 5.4 | 0.7 | 4.3 | 5.9 |
| Dachshund | 16 | 5.4 | 0.6 | 5.7 | 0.6 | 4.0 | 6.0 |
| Rhodesian Ridgeback | 17 | 5.2 | 0.5 | 5.3 | 0.5 | 4.2 | 6.0 |
|  |  |  |  |  |  |  |  |
| **Spaniel/Pointer** |  |  |  |  |  |  |  |
| Cavalier King Charles Spaniel | 93 | 5.0 | 0.5 | 5.0 | 0.8 | 3.9 | 6.0 |
| Cocker Spaniel | 43 | 5.1 | 0.4 | 5.2 | 0.5 | 4.2 | 5.9 |
| English Springer Spaniel | 18 | 5.0 | 0.5 | 5.1 | 0.8 | 4.2 | 5.8 |
| Irish Setter | 15 | 5.4 | 0.4 | 5.3 | 0.4 | 4.7 | 6.0 |
| Italian Spinone | 12 | 4.8 | 0.8 | 4.9 | 1.1 | 3.6 | 5.8 |
| Springer Spaniel | 36 | 5.0 | 0.4 | 4.9 | 0.4 | 4.3 | 6.0 |
| Weimaraner | 24 | 5.1 | 0.5 | 5.2 | 0.7 | 3.9 | 5.9 |
|  |  |  |  |  |  |  |  |
| **Other** |  |  |  |  |  |  |  |
| Dalmatian | 10 | 5.4 | 0.3 | 5.3 | 0.4 | 5.1 | 5.9 |
| Jack Russell Terrier | 38 | 5.2 | 0.6 | 5.3 | 0.6 | 3.5 | 6.0 |

§ Unit of measurement: mmol/L; SD = standard deviation; IQR = interquartile range; Min. = minimum value recorded; Max. = maximum value recorded.

**Table S in S1 File. Statistical analysis of the effects of age, sex and neutering status**. This table shows the p values of a linear mixed effects model to assess the effect of age, sex and neutering status, and all two-way and three-way interactions on each serum biochemical analyte, taking breed as a random effect.

| **Analyte** | **Age** | **Sex** | **Neutering status** | **Age*sex** | **Age*neutering status** | **Sex*neutering status** | **Age*sex*neutering status** |
| --- | --- | --- | --- | --- | --- | --- | --- |
| **Total protein** | <0.0001 | 0.0049 | 0.0012 | 0.3664 | 0.1886 | 0.8393 | 0.7177 |
| **Albumin** | <0.0001 | 0.4579 | 0.0840 | 0.9317 | 0.3301 | 0.9392 | 0.6407 |
| **Globulin** | <0.0001 | 0.0092 | 0.0147 | 0.1361 | 0.3476 | 0.8877 | 0.6625 |
| **Sodium** | 0.4376 | 0.7232 | 0.7260 | 0.7325 | 0.3249 | 0.1802 | 0.8719 |
| **Potassium** | <0.0001 | 0.0101 | 0.0213 | 0.4509 | 0.2613 | 0.0459 | 0.6452 |
| **Chloride** | 0.0730 | 0.7141 | 0.0004 | 0.1585 | 0.1049 | 0.0349 | 0.4526 |
| **Calcium** | <0.0001 | 0.0722 | 0.1047 | 0.3589 | 0.3676 | 0.1431 | 0.1389 |
| **Phosphorus** | <0.0001 | 0.0692 | 0.1203 | 0.0848 | 0.0349 | 0.3921 | 0.5577 |
| **Urea** | 0.0053 | 0.6335 | 0.3402 | 0.4936 | 0.8983 | 0.3714 | 0.4385 |
| **Creatinine** | 0.0004 | 0.0109 | 0.0007 | 0.0964 | 0.1196 | 0.2252 | 0.5091 |
| **Cholesterol** | 0.0163 | 0.5509 | 0.0062 | 0.1271 | 0.0216 | <0.0001 | 0.6104 |
| **Total bilirubin** | 0.0006 | 0.4699 | 0.2293 | 0.5436 | 0.0276 | 0.9885 | 0.0045 |
| **ALT** | <0.0001 | 0.0203 | <0.0001 | 0.8922 | 0.0593 | 0.0199 | 0.9041 |
| **ALP** | <0.0001 | 0.2325 | 0.0187 | 0.5538 | 0.9518 | 0.4428 | 0.5708 |
| **CK** | <0.0001 | 0.7109 | 0.8376 | 0.7003 | 0.2339 | 0.6331 | 0.0099 |
| **Amylase** | <0.0001 | <0.0001 | 0.2065 | 0.6277 | 0.5798 | <0.0001 | 0.0118 |
| **Lipase** | <0.0001 | <0.0001 | <0.0001 | 0.7269 | 0.2235 | <0.0001 | 0.4002 |
| **Glucose** | 0.8430 | 0.2833 | 0.1721 | 0.4551 | 0.8766 | 0.4421 | 0.4100 |

**Table T in S1 File. Principal component analysis – E**igenvalues of the correlation matrix

| **Principal**  **component** | **Eigenvalue** | **Associated variation (%)** | **Cumulative variation (%)** |
| --- | --- | --- | --- |
| **1** | **3.09** | **17.18** | **17.18** |
| **2** | **2.41** | **13.38** | **30.56** |
| **3** | **1.73** | **9.61** | **40.18** |
| **4** | **1.63** | **9.04** | **49.22** |
| **5** | **1.40** | **7.80** | **57.02** |
| **6** | **1.09** | **6.04** | **63.06** |
| 7 | 0.99 | 5.49 | 68.55 |
| 8 | 0.89 | 4.96 | 73.52 |
| 9 | 0.82 | 4.58 | 78.10 |
| 10 | 0.74 | 4.09 | 82.18 |
| 11 | 0.67 | 3.72 | 85.90 |
| 12 | 0.57 | 3.18 | 89.08 |
| 13 | 0.52 | 2.88 | 91.96 |
| 14 | 0.48 | 2.67 | 94.63 |
| 15 | 0.40 | 2.22 | 96.85 |
| 16 | 0.34 | 1.87 | 98.72 |
| 17 | 0.23 | 1.27 | 99.99 |
| 18 | 0.00 | 0.01 | 100 |

**Table U in S1 File. Statistical analysis of pairwise comparisons of serum biochemical analytes between the pure breed and mixed breed dogs.** This table shows the -log_10_(p value) of two-sample Kolmogorov-Smirnov tests to compare the distributions of residuals for the pure breed *versus* mixed breed dogs. Residuals were defined as the observed values minus the estimated fixed effects of age, sex and neutering status. A Bonferroni correction to account for multiple comparisons was applied, yielding a threshold for significance of 10^-4^ (i.e. 4 as stated in the table). The -log_10_(p value) ≥ than 4 were bold faced.

| Breed | **Total protein** | **Albumin** | **Globulin** | **Sodium** | **Potassium** | **Chloride** | **Calcium** | **Phosphorus** | **Urea** | **Creatinine** | **Cholesterol** | **Total bilirubin** | **ALT** | **ALP** | **CK** | **Amylase** | **Lipase** | **Glucose** |
| --- | --- | --- | --- | --- | --- | --- | --- | --- | --- | --- | --- | --- | --- | --- | --- | --- | --- | --- |
| **Ancient** |  |  |  |  |  |  |  |  |  |  |  |  |  |  |  |  |  |  |
| Maltese Terrier | 0.75 | 0.32 | 1.02 | 1.19 | 0.53 | 0.80 | 0.85 | 0.10 | 0.65 | 3.67 | 1.05 | 3.27 | 1.15 | 0.10 | 0.32 | 0.38 | 0.07 |  |
| Shar Pei | 2.79 | 2.02 | **5.31** | 0.24 | 0.13 | 0.43 | 0.95 | 0.14 | 1.17 | 0.33 | 1.10 | 0.27 | 2.27 | 0.32 | 1.47 | 1.12 | 2.43 | 0.58 |
| Siberian Husky | 0.14 | 2.17 | 0.81 | 0.04 | 1.08 | 0.84 | 0.28 | 0.15 | 0.25 | 0.22 | 0.05 | 0.40 | 0.17 | 0.05 | 0.70 | 0.96 | 1.33 |  |
| Tibetan Terrier | 0.68 | 0.82 | 0.52 | 0.16 | 0.19 | 0.12 | 0.77 | 0.09 | 0.75 | 0.84 | 1.86 | 0.82 | 0.74 | 1.37 | 0.68 | 0.02 | 1.94 |  |
|  |  |  |  |  |  |  |  |  |  |  |  |  |  |  |  |  |  |  |
| **Toy** |  |  |  |  |  |  |  |  |  |  |  |  |  |  |  |  |  |  |
| Chihuahua | 0.00 | 0.54 | 0.40 | 0.36 | 0.31 | 0.14 | 0.04 | 0.98 | 2.20 | 6.49 | 1.68 | 1.93 | 1.33 | 0.22 | 0.79 | 1.19 | 0.97 |  |
| Pug | 0.45 | 1.21 | 0.73 | **5.87** | **5.19** | **7.28** | 0.87 | 0.62 | 1.43 | 1.49 | 1.96 | 0.87 | 0.00 | 2.06 | 1.77 | 0.68 | 2.81 |  |
| Shih Tzu | 0.67 | 1.42 | 0.96 | 1.04 | 0.15 | 3.24 | 0.07 | 0.01 | 0.79 | 2.88 | 1.76 | 0.55 | 0.78 | 1.74 | 0.08 | **4.40** | 3.14 | 0.50 |
|  |  |  |  |  |  |  |  |  |  |  |  |  |  |  |  |  |  |  |
| **Working** |  |  |  |  |  |  |  |  |  |  |  |  |  |  |  |  |  |  |
| Dobermann | 0.09 | 1.41 | 0.02 | 0.53 | 0.19 | 0.23 | 0.06 | 3.46 | 0.38 | 0.17 | 0.27 | 0.22 | 0.26 | 0.48 | 0.39 | 1.10 | 1.57 | 0.43 |
| German Shepherd Dog | 0.21 | 2.99 | 0.81 | 0.08 | 0.35 | 3.26 | 0.99 | 0.65 | 1.47 | **6.69** | 1.24 | 0.93 | 0.41 | **6.40** | 0.28 | 2.74 | 2.72 | 0.50 |
| Miniature Schnauzer | 0.01 | 0.46 | 0.45 | 0.77 | 0.97 | 0.75 | 0.76 | 0.16 | 0.62 | 3.65 | 0.09 | 0.27 | 0.08 | 0.34 | 1.04 | 0.76 | 2.29 |  |
| Schnauzer | 0.24 | 0.08 | 0.45 | 0.26 | 1.89 | 0.03 | 0.01 | 0.74 | 0.89 | 1.45 | 0.41 | 1.93 | 0.12 | 0.01 | 0.23 | 0.19 | 1.07 |  |
|  |  |  |  |  |  |  |  |  |  |  |  |  |  |  |  |  |  |  |
| **Sight hound** |  |  |  |  |  |  |  |  |  |  |  |  |  |  |  |  |  |  |
| Greyhound | 1.83 | 0.60 | 2.14 | 0.13 | 0.05 | 0.08 | 0.28 | 1.39 | 0.28 | 2.28 | 0.73 | 0.13 | 0.97 | 0.19 | 0.07 | 0.05 | 0.63 |  |
| Lurcher | 1.02 | 0.08 | 2.15 | 0.18 | 2.88 | 0.69 | 0.62 | 0.22 | 0.31 | 0.18 | 1.00 | 0.03 | 0.23 | 0.25 | 0.33 | 0.02 | 0.94 |  |
|  |  |  |  |  |  |  |  |  |  |  |  |  |  |  |  |  |  |  |
| **Mastiff-like** |  |  |  |  |  |  |  |  |  |  |  |  |  |  |  |  |  |  |
| Boston Terrier | **4.92** | 2.35 | 3.61 | 0.35 | 0.07 | 1.43 | **4.90** | 0.32 | 2.80 | 0.47 | 2.35 | 3.34 | 0.18 | 1.23 | 1.05 | 1.38 | 1.25 |  |
| Boxer | 0.80 | 0.29 | 0.38 | 0.53 | 0.25 | 1.40 | 2.20 | **11.28** | 1.28 | **5.77** | 3.10 | 1.36 | **4.91** | 0.40 | 1.44 | **15.95** | **15.66** | 2.11 |
| Bullmastiff | 0.39 | 0.35 | 1.87 | 0.52 | 1.69 | 2.36 | 0.09 | 0.93 | 0.27 | 2.24 | 3.63 | 0.08 | 2.41 | 0.78 | 0.56 | 0.49 | 2.22 | 0.12 |
| Bulldog | 0.08 | 0.99 | 0.54 | 0.56 | **5.29** | 2.35 | 0.36 | **4.17** | 0.45 | 0.02 | 1.43 | 2.30 | 2.16 | 0.74 | 2.38 | **6.24** | 0.13 | 0.81 |
| Dogue de Bordeaux | 0.03 | 1.11 | 1.29 | 2.46 | 0.02 | 0.89 | 0.15 | 3.43 | 0.19 | 1.03 | 3.39 | 0.18 | 0.33 | 0.82 | 0.05 | 1.93 | 0.10 |  |
| French Bulldog | 0.13 | 0.03 | 0.54 | 0.12 | 0.57 | 3.04 | 0.49 | 1.30 | 0.21 | 0.26 | 0.29 | 2.13 | 0.01 | 1.70 | 1.90 | 0.53 | 0.44 |  |
| Mastiff | 0.96 | 0.18 | 0.42 | 0.93 | 0.27 | 1.36 | 0.79 | 0.47 | 0.04 | 2.94 | 0.05 | 0.17 | 0.50 | 0.58 | 0.37 | 1.89 | 0.84 |  |
| Staffordshire Bull Terrier | 0.18 | 1.59 | 0.97 | 1.56 | 0.80 | 3.49 | 0.47 | 1.89 | 0.22 | 1.01 | **4.66** | 0.64 | 0.39 | 0.11 | 3.03 | 1.29 | 1.19 | 3.26 |
|  |  |  |  |  |  |  |  |  |  |  |  |  |  |  |  |  |  |  |
| **Retriever/other Mastiff-like** | |  |  |  |  |  |  |  |  |  |  |  |  |  |  |  |  |  |
| Flat-Coated Retriever | 2.77 | 1.56 | 1.41 | 1.01 | 1.22 | 0.39 | 1.31 | 1.30 | 0.00 | 1.07 | 0.00 | 0.50 | 0.95 | 0.69 | 1.13 | 1.73 | 0.72 |  |
| Golden Retriever | 0.92 | 1.43 | 0.92 | 0.12 | 0.19 | 0.85 | 0.45 | 2.46 | 2.93 | 0.01 | **6.79** | 0.78 | 0.51 | 1.08 | 0.34 | **4.91** | **15.66** | 0.39 |
| Great Dane | 1.79 | 0.84 | 2.88 | 0.05 | 0.16 | 0.17 | 0.04 | 0.23 | 0.39 | 3.33 | 0.23 | 2.38 | 0.13 | 2.60 | 0.57 | 1.99 | 0.91 | 0.69 |
| Labrador Retriever | 2.96 | 0.59 | 2.64 | 0.01 | 1.19 | 1.65 | 1.46 | 0.07 | 0.91 | **5.14** | 2.14 | 0.52 | 1.27 | 3.68 | 0.95 | 0.44 | **5.98** | 1.11 |
| Leonberger | 1.66 | 0.24 | 0.90 | 1.07 | 0.81 | 0.83 | 0.08 | 1.69 | 0.14 | 2.71 | 1.57 | 0.33 | 0.23 | 0.98 | 1.61 | 1.07 | 0.10 |  |
| Newfoundland | 0.23 | 0.01 | 0.59 | 0.80 | 1.19 | 0.86 | 0.91 | 2.34 | 0.54 | 0.87 | 0.10 | 0.01 | 1.28 | 0.28 | 0.56 | 0.57 | 1.39 | 0.20 |
| Retriever | 0.52 | 3.46 | 0.19 | 0.00 | 1.47 | 0.19 | 1.22 | 0.49 | 0.60 | 1.26 | 3.32 | 0.55 | 0.51 | 0.42 | 0.76 | 1.14 | **5.60** | 1.14 |
| Rottweiler | 0.23 | 1.37 | 1.26 | 0.07 | 0.91 | 2.39 | 0.76 | **4.37** | 0.16 | 2.15 | **6.45** | 0.74 | 3.28 | 3.04 | 3.58 | **5.70** | 0.73 | 0.17 |
|  |  |  |  |  |  |  |  |  |  |  |  |  |  |  |  |  |  |  |
| **Herding** |  |  |  |  |  |  |  |  |  |  |  |  |  |  |  |  |  |  |
| Border Collie | 1.48 | 0.32 | 1.89 | 0.37 | 1.40 | 1.06 | 0.69 | 0.19 | 0.46 | 0.72 | 1.58 | 0.06 | 0.49 | 0.07 | 0.58 | 0.18 | 3.17 | 0.86 |
| Collie | 1.07 | 1.55 | 0.23 | 0.40 | 0.17 | 0.09 | 0.77 | 0.25 | 0.71 | 2.13 | 0.45 | 0.07 | 0.28 | 0.03 | 0.72 | 0.24 | 2.41 | 0.00 |
| Shetland Sheepdog | 0.08 | 0.67 | 0.34 | 0.16 | 0.40 | 0.91 | 0.58 | 2.14 | 0.25 | 0.03 | 0.02 | 3.35 | 0.04 | 0.07 | 0.25 | 0.72 | 0.29 |  |
|  |  |  |  |  |  |  |  |  |  |  |  |  |  |  |  |  |  |  |
| **Terrier** |  |  |  |  |  |  |  |  |  |  |  |  |  |  |  |  |  |  |
| Airedale | 1.72 | 0.27 | 0.86 | 0.84 | 0.04 | 0.52 | 2.49 | 0.79 | 0.51 | 0.10 | 0.95 | 0.79 | 0.68 | 0.03 | 0.01 | 0.96 | 1.89 |  |
| Border Terrier | 0.18 | 1.32 | 0.33 | 0.78 | 0.45 | **4.47** | 0.00 | 0.79 | 0.27 | 0.70 | 0.10 | 1.33 | **4.43** | 0.08 | 0.44 | 0.90 | 0.31 | 0.93 |
| Norfolk Terrier | 0.07 | 0.00 | 0.43 | 0.20 | 0.00 | 0.28 | 0.18 | 0.64 | 0.17 | 0.15 | 0.57 | 0.56 | 0.20 | 0.34 | 1.11 | 0.40 | 1.01 |  |
| West Highland White Terrier | 1.05 | **6.07** | 0.29 | 0.43 | 1.58 | 3.41 | 1.21 | **7.44** | 0.27 | **6.16** | 0.03 | 3.70 | 2.71 | **6.79** | **7.93** | **7.15** | **5.53** | 0.76 |
| Yorkshire Terrier | 0.18 | 1.38 | 0.89 | 0.67 | 2.02 | 0.22 | 0.56 | 0.99 | 0.85 | 4.34 | 3.07 | 1.91 | 0.07 | 1.06 | 1.66 | 1.10 | 1.44 | 0.57 |
|  |  |  |  |  |  |  |  |  |  |  |  |  |  |  |  |  |  |  |
| **Scent hound** |  |  |  |  |  |  |  |  |  |  |  |  |  |  |  |  |  |  |
| Basset Hound | 0.68 | 0.09 | 0.80 | 0.06 | 0.14 | 1.55 | 0.02 | 0.47 | 0.76 | 3.38 | 0.95 | 1.17 | **4.13** | 0.35 | 2.39 | 0.04 | 1.43 | 0.20 |
| Beagle | 0.25 | 0.45 | 0.38 | 0.17 | 0.07 | 0.09 | 0.45 | 1.02 | 0.41 | 2.69 | 0.95 | 0.45 | 1.70 | 1.37 | 0.02 | 2.42 | 0.82 | 0.79 |
| Dachshund | 0.30 | 0.30 | 0.75 | 0.04 | 0.37 | 0.10 | 1.70 | 0.34 | 0.87 | **10.11** | 1.24 | **5.20** | 0.13 | 0.90 | 1.15 | 1.78 | 0.96 | 2.19 |
| Rhodesian Ridgeback | 0.15 | 0.17 | 0.17 | 0.47 | 0.35 | 0.76 | 1.13 | 0.25 | 0.33 | 0.31 | 0.12 | 0.05 | 1.96 | 0.49 | 0.20 | 0.12 | 0.79 | 0.24 |
|  |  |  |  |  |  |  |  |  |  |  |  |  |  |  |  |  |  |  |
| **Spaniel/Pointer** |  |  |  |  |  |  |  |  |  |  |  |  |  |  |  |  |  |  |
| Cavalier King Charles Spaniel | 1.11 | 1.63 | **4.18** | 1.00 | 0.56 | **9.08** | 0.48 | **9.81** | 2.88 | **14.20** | 3.08 | **8.13** | **10.04** | **4.02** | 3.81 | 3.17 | 2.54 | 0.06 |
| Cocker Spaniel | 0.64 | 0.18 | 0.06 | 0.04 | 0.05 | 0.67 | 0.90 | 0.96 | 1.54 | **15.66** | 0.13 | 2.54 | 2.56 | 0.23 | 0.31 | 2.78 | **8.32** | 0.60 |
| English Springer Spaniel | 0.81 | 1.00 | 0.89 | 0.34 | 0.45 | 0.29 | 0.64 | 0.03 | 1.18 | 0.47 | 0.43 | 0.60 | 2.22 | 0.28 | 1.30 | 0.34 | 1.48 | 0.37 |
| German Shorthaired Pointer | 1.66 | 0.95 | 1.52 | 0.88 | 1.03 | 0.35 | 1.98 | 0.10 | 0.65 | 0.02 | 0.19 | 0.06 | 0.54 | 0.01 | 0.06 | 0.26 | 0.22 |  |
| Gordon Setter | 1.01 | 0.40 | 0.84 | 1.27 | 2.43 | 0.29 | 0.78 | 0.01 | 0.83 | 1.01 | 0.90 | 0.22 | 0.90 | 0.31 | 0.15 | 0.07 | 0.44 |  |
| Hungarian Vizsla | 1.39 | 0.93 | 0.31 | 0.01 | 0.23 | 0.35 | 2.09 | 0.61 | 2.55 | 1.35 | 0.63 | 0.94 | 0.27 | 0.45 | 0.23 | 1.85 | 0.27 |  |
| Irish Setter | 0.01 | 0.35 | 0.04 | 0.49 | 0.52 | 0.84 | 1.07 | 0.18 | 0.99 | 3.20 | 0.52 | 1.35 | 0.03 | 0.07 | 0.75 | 0.85 | 3.50 | 0.83 |
| Italian Spinone | 0.10 | 0.01 | 0.02 | 0.81 | 2.88 | 0.10 | 0.69 | 1.96 | 2.20 | 2.56 | 0.05 | 0.23 | 0.01 | 0.02 | 0.29 | 0.53 | 0.24 | 0.74 |
| Pointer | 0.23 | 1.27 | 0.33 | 0.44 | 1.19 | 0.22 | 0.43 | 0.85 | 0.27 | 0.05 | 0.97 | 0.20 | 0.00 | 0.58 | 0.01 | 1.08 | 0.07 |  |
| Springer Spaniel | 0.63 | 0.76 | 1.11 | 0.09 | 0.31 | 0.35 | 0.00 | 2.28 | 0.71 | 3.08 | 0.05 | 0.51 | 2.93 | 0.40 | 0.99 | 0.21 | **5.25** | 0.59 |
| Weimaraner | 0.38 | **6.17** | 0.79 | 0.02 | 0.45 | 1.64 | 0.27 | 0.25 | 0.68 | 0.02 | 0.53 | 0.13 | 0.11 | 1.45 | 0.29 | 0.06 | **4.89** | 0.09 |
|  |  |  |  |  |  |  |  |  |  |  |  |  |  |  |  |  |  |  |
| **Other** |  |  |  |  |  |  |  |  |  |  |  |  |  |  |  |  |  |  |
| Bichon Frise | 0.38 | 0.42 | 0.22 | 0.26 | 0.00 | 0.14 | 0.21 | 0.81 | 1.70 | 3.79 | 0.05 | 1.02 | 0.31 | 0.29 | 0.82 | 0.56 | 0.27 |  |
| Collie cross | 0.15 | 0.77 | 0.66 | 0.22 | 0.26 | 0.44 | 0.31 | 0.26 | 0.04 | 0.24 | 0.18 | 0.98 | 1.09 | 0.15 | 0.08 | 0.14 | 0.69 |  |
| Dalmatian | 0.72 | 0.12 | 0.66 | 0.71 | 1.26 | 0.12 | 0.85 | 1.62 | 1.02 | 0.47 | 0.04 | 0.08 | 1.22 | 0.09 | 0.09 | 0.53 | 0.91 | 2.76 |
| Jack Russell Terrier | 0.02 | 2.58 | 1.06 | 0.50 | 0.58 | 0.05 | 0.40 | 1.22 | 0.26 | **11.71** | 1.25 | 3.30 | 0.26 | 0.89 | 1.86 | 1.86 | 0.06 | 0.50 |
| Labrador cross | 0.36 | 0.02 | 0.52 | 0.63 | 0.10 | 0.18 | 0.03 | 0.24 | 0.12 | 0.23 | 0.50 | 0.69 | 0.13 | 0.15 | 0.31 | 0.02 | 0.05 |  |
| Lhasa Apso | 2.66 | 0.28 | 1.58 | 0.83 | 0.03 | 1.04 | 0.44 | 1.10 | 1.30 | 0.90 | 0.46 | 0.82 | 1.42 | 0.73 | 0.18 | 2.04 | 1.38 |  |
| Poodle | 1.15 | 0.04 | 1.85 | 0.52 | 1.37 | 0.82 | 0.62 | 2.58 | 0.60 | 0.01 | 0.39 | 0.85 | 0.41 | 1.71 | 0.76 | 1.74 | 0.17 |  |
| Standard Poodle | 1.91 | 0.32 | 0.77 | 0.17 | 0.31 | 1.45 | 0.01 | 0.22 | 0.51 | 0.29 | 0.01 | 0.05 | 0.17 | 0.17 | 0.19 | 0.32 | 0.60 |  |

**Table V in S1 File. Tentative breed-specific reference intervals for the Labrador Retriever (n=327; 19 intact females, 98 neutered females, 91 intact males and 119 neutered males). The median (minimum, maximum) age was 6.08 (0.67, 14.00) years.**

| **Analyte** | **SI Units** | **RI^a^** | **Lower reference**  **limit 90% CI^b^** | **Upper reference**  **limit 90% CI^b^** |
| --- | --- | --- | --- | --- |
| Total protein | g/L | 53.1~67.8 | 52.6~53.7 | 67.0~69.1 |
| Albumin | g/L | 28.3~38.4 | 28.1~29.1 | 38.1~38.7 |
| Globulin | g/L | 21.3~34.5 | 21.1~21.4 | 33.3~38.2 |
| Sodium | mmol/L | 143.0~153.0^*^ | 142.0~143.6 | 153.0^*^~153.0^*^ |
| Potassium | mmol/L | 4.10~5.30 | 4.10~4.20 | 5.20~5.40 |
| Chloride | mmol/L | 108.0~116.0 | 106.8~108.5 | 116.0~117.0 |
| Calcium | mmol/L | 2.19~2.66 | 2.17~2.22 | 2.64~2.68 |
| Phosphorus | mmol/L | 0.88~1.87 | 0.81~0.92 | 1.82~1.95 |
| Creatinine | μmol/L | 71~122^#^ | 67~77 | 121^#^~122^#^ |
| Cholesterol | mmol/L | 3.6~8.1 | 3.5~3.8 | 7.8~8.5 |
| Total bilirubin | μmol/L | 0~2.4 | 0~0 | 2.3~2.4 |
| ALT | U/L | 22~76 | 19~24 | 74~82 |
| CK | U/L | 70~346 | 66~74 | 313~366 |
| ALP | U/L | 19^§^~212 | 19^§^~20^§^ | 170~239 |
| Amylase | U/L | 388~1171 | 364~412 | 1136~1217 |
| Lipase | U/L | 102~592 | 91~110 | 573~720 |

^a^ RI: reference interval established by non-parametric method; ^b^ CI: confidence interval established by non-parametric method; ^*^ may be underestimated due to > 2% recorded sodium equal to the existing RVC DL upper reference limits 153 mmol/L; ^#^ may be underestimated due to > 2% recorded creatinine equal to the existing RVC DL upper reference limits 122 μmol/L; ^§^ may be overestimated due to > 2% recorded ALP equal to the existing RVC DL lower reference limits 19 U/L.

**Table W in S1 File. Tentative breed-specific reference intervals for the Cavalier King Charles Spaniels (n=174; 20 intact females, 60 neutered females, 26 intact males and 68 neutered males). The median (minimum, maximum) age was 5.25 (0.75, 18.00) years.**

| **Analyte** | **SI Units** | **RI^a^** | **Lower reference**  **limit 90% CI^b^** | **Upper reference**  **limit 90% CI^b^** |
| --- | --- | --- | --- | --- |
| Total protein | g/L | 53.0~66.9 | 51.6~54.1 | 66.5~68.3 |
| Albumin | g/L | 29.8~38.6 | 28.1~30.2 | 38.4~39.0 |
| Globulin | g/L | 21.2~32.5 | 21.0~21.6 | 30.9~35.9 |
| Sodium | mmol/L | 143.8~152.7 | 142.0~144.0 | 152.2~153.0 |
| Potassium | mmol/L | 4.10~5.06 | 4.00~4.10 | 5.00~5.40 |
| Chloride | mmol/L | 105.2~116.0 | 105.0~106.0 | 114.1~118.0 |
| Calcium | mmol/L | 2.27~2.67 | 2.13~2.33 | 2.65~2.68 |
| Phosphorus | mmol/L | 1.0~1.9 | 0.84~1.04 | 1.85~1.98 |
| Creatinine | μmol/L | 57.4~108.6 | 56~61 | 105~117 |
| Cholesterol | mmol/L | 3.9~8.8 | 3.4~4.2 | 8.5~8.9 |
| Total bilirubin | μmol/L | 0~2.1 | 0~0 | 1.9~2.4 |
| ALT | U/L | 14~70 | 13~15 | 50~81 |
| CK | U/L | 73~359 | 63~78 | 326~392 |
| ALP | U/L | 19^*^~222 | 19^*^~20^*^ | 190~254 |
| Amylase | U/L | 356~1137 | 216~431 | 1091~1220 |
| Lipase | U/L | 142~707 | 124~166 | 576~924 |

^a^ RI: reference interval established by non-parametric method; ^b^ CI: confidence interval established by non-parametric method; ^*^ may be overestimated due to > 2% recorded ALP equal to the existing RVC DL lower reference limits 19 U/L.

**Table X in S1 File. Tentative breed-specific reference intervals for the German Shepherd Dog (n=160; 11 intact females, 58 neutered females, 35 intact males and 56 neutered males) . The median (minimum, maximum) age was 7.00 (0.75, 12.33) years.**

| **Analyte** | **SI Units** | **RI^a^** | **Lower reference**  **limit 90% CI^b^** | **Upper reference**  **limit 90% CI^b^** |
| --- | --- | --- | --- | --- |
| Total protein | g/L | 53.1~68.6 | 52.1~54.2 | 67.7~71.0 |
| Albumin | g/L | 28.6~38.1 | 28.2~29.1 | 37.3~38.7 |
| Globulin | g/L | 22.2~37.9 | 21.2~22.4 | 34.9~38.6 |
| Sodium | mmol/L | 143.3~152.3 | 142.0~144.0 | 152.0~153.0 |
| Potassium | mmol/L | 4.1~5.30 | 3.90~4.20 | 5.20~5.50 |
| Chloride | mmol/L | 107.0~117.3 | 105.6~108.0 | 117.0~118.0 |
| Calcium | mmol/L | 2.24~2.67 | 2.16~2.31 | 2.67~2.70 |
| Phosphorus | mmol/L | 0.81~1.86 | 0.80~0.88 | 1.76~1.95 |
| Creatinine | μmol/L | 75~122^§^ | 70~80 | 121^§^~122^§^ |
| Cholesterol | mmol/L | 3.6~8.0 | 3.3~3.7 | 7.7~8.6 |
| Total bilirubin | μmol/L | 0~2.2 | 0~0 | 2.1~2.4 |
| ALT | U/L | 19~81 | 17~22 | 79~85 |
| CK | U/L | 63~316 | 62~65 | 300~353 |
| ALP | U/L | 19^*^~136 | 19^*^~19^*^ | 119~271 |
| Amylase | U/L | 372~1090 | 204~458 | 1042~1146 |
| Lipase | U/L | 99~632 | 76~112 | 596~885 |

^a^ RI: reference interval established by non-parametric method; ^b^ CI: confidence interval established by non-parametric method; ^§^ may be underestimated due to > 2% recorded creatinine equal to the existing RVC DL upper reference limits 122 μmol/L;^*^ may be overestimated due to > 2% recorded ALP equal to the existing RVC DL lower reference limits 19 U/L.

**Table Y in S1 File. Tentative breed-specific reference intervals for the Boxer (n=146; 16 intact females, 48 neutered females, 33 intact males and 49 neutered males). The median (minimum, maximum) age was 6.08 (0.08, 13.33) years.**

| **Analyte** | **SI Units** | **RI^a^** | **Lower reference**  **limit 90% CI^b^** | **Upper reference**  **limit 90% CI^b^** |
| --- | --- | --- | --- | --- |
| Total protein | g/L | 54.6~69.1 | 53.8~56.2 | 68.3~69.8 |
| Albumin | g/L | 28.9~38.0 | 28.6~29.9 | 37.8~39.0 |
| Globulin | g/L | 21.7~35.7 | 21.4~22.3 | 34.6~37.8 |
| Sodium | mmol/L | 142.7^*^~153.0^§^ | 142.0^*^~143.8^*^ | 152.2^§^~153.0^§^ |
| Potassium | mmol/L | 4.00~5.20 | 3.90~4.10 | 5.10~5.30 |
| Chloride | mmol/L | 107.3~115.9 | 106.0~108.0 | 115.0~117.1 |
| Calcium | mmol/L | 2.25~2.64 | 2.18~2.28 | 2.61~2.70 |
| Phosphorus | mmol/L | 1.02~1.93 | 0.92~1.11 | 1.84~1.96 |
| Creatinine | μmol/L | 81~122^$^ | 78~85 | 121^$^~122^$^ |
| Cholesterol | mmol/L | 4.0~8.7 | 3.8~4.4 | 8.6~8.9 |
| Total bilirubin | μmol/L | 0~2.3 | 0~0 | 2.1~2.4 |
| ALT | U/L | 23~77 | 19~27 | 74~87 |
| CK | U/L | 71~370 | 69~78 | 329~390 |
| ALP | U/L | 20^#^~172 | 19^#^~22^#^ | 156~277 |
| Amylase | U/L | 502~1208 | 338~569 | 1166~1244 |
| Lipase | U/L | 292~1026 | 124~326 | 971~1104 |

^a^ RI: reference interval established by non-parametric method; ^b^ CI: confidence interval established by non-parametric method; ^*^ may be overestimated due to > 2% recorded sodium equal to the existing RVC DL lower reference limits 142 mmol/L; ^§^ may be underestimated due to > 2% recorded sodium equal to the existing RVC DL upper reference limits 153 mmol/L; ^$^ may be underestimated due to > 2% recorded creatinine equal to the existing RVC DL upper reference limits 122 μmol/L; ^#^ may be overestimated due to > 2% recorded ALP equal to the existing RVC DL lower reference limits 19 U/L;.

**Table Z in S1 File. Tentative breed-specific reference intervals for the Golden Retriever (n=121; 18 intact females, 48 neutered females, 28 intact males and 27 neutered males). The median (minimum, maximum) age was 6.75 (0.58, 13.83) years.**

| **Analyte** | **SI Units** | **RI^a^** | **Lower reference**  **limit 90% CI^b^** | **Upper reference**  **limit 90% CI^b^** |
| --- | --- | --- | --- | --- |
| Total protein | g/L | 54.0~68.3 | 51.4~54.7 | 64.8~70.1 |
| Albumin | g/L | 28.6~37.1 | 28.3~29.2 | 36.7~38.0 |
| Globulin | g/L | 21.7~34.3 | 21.2~22.7 | 32.7~40.0 |
| Sodium | mmol/L | 143.0~153.0^§^ | 142.0~143.1 | 152.6^§^~153.0^§^ |
| Potassium | mmol/L | 4.10~5.20 | 3.90~4.10 | 5.10~5.30 |
| Chloride | mmol/L | 107.3~116.0 | 105.0~108.2 | 115.0~117.0 |
| Calcium | mmol/L | 2.22~2.68 | 2.15~2.29 | 2.64~2.69 |
| Phosphorus | mmol/L | 0.83~1.89 | 0.82~0.91 | 1.65~1.99 |
| Creatinine | μmol/L | 69~119 | 65~72 | 117~120 |
| Cholesterol | mmol/L | 4.2~8.9^#^ | 3.3~4.6 | 8.8^#^~8.9^#^ |
| Total bilirubin | μmol/L | 0~2.4^*^ | 0~0.1 | 2.2^*^~2.4^*^ |
| ALT | U/L | 19~69 | 17~20 | 65~86 |
| CK | U/L | 62^$^~370 | 62^$^~76^$^ | 322~390 |
| ALP | U/L | 20~166 | 19~26 | 108~214 |
| Amylase | U/L | 325~1175 | 186~383 | 943~1236 |
| Lipase | U/L | 80~512 | 78~90 | 390~677 |

^a^ RI: reference interval established by non-parametric method; ^b^ CI: confidence interval established by non-parametric method; ^§^ may be underestimated due to > 2% recorded sodium equal to the existing RVC DL upper reference limits 153 mmol/L; ^#^ may be underestimated due to > 2% recorded cholesterol equal to the existing RVC DL upper reference limits 8.9 mmol/L; ^*^ may be underestimated due to > 2% recorded total bilirubin equal to the existing RVC DL upper reference limits 2.4 μmol/L; ^$^ may be overestimated due to > 2% recorded CK equal to the existing RVC DL lower reference limits 61 U/L.
