# Supplementary material for: Serum Biochemical Phenotypes in the Domestic Dog
Source: PLoS One. 2016 Feb 26;11(2):e0149650. doi: 10.1371/journal.pone.0149650 (PMC4769346; doi:10.1371/journal.pone.0149650)
Supplement: S1 Materials and Methods — (DOCX) [file pone.0149650.s010.docx]

**SUPPORTING INFORMATION**

**Materials and Methods** (detailed version)

*Inclusion and exclusion criteria*

Serum biochemical profiles were collected from the database of the Royal Veterinary College (RVC) Diagnostic Laboratory (DL) from January 2004 to October 2013, which receives samples from both its own small animal hospitals (the Queen Mother Hospital for Animals, a primary, secondary and tertiary care center, and the Beaumont Sainsbury Animal Hospital, a primary care center) and external veterinary practices. The database was interrogated over the period of time from its inception in January 2004 to October 2013, comparing each dog’s profile to the generic reference intervals used by the DL for all mixed and pure breed dogs, derived by reference intervals studies carried out on approximately 40 healthy staff dogs of various breeds, ages, sexes and neutering status. Serum biochemical profiles were included in the study if every analyte fell within the reference interval, excluding glucose measurement, which were interpreted separately. Profiles with missing data values were excluded from analysis, as were those associated with insufficiently specific breed descriptors or missing signalment information. Data were collected from both pure breed dogs and a control group of mixed breed dogs, anonymizing all patient details in accordance with general guidelines laid down by the Royal Veterinary College Ethics and Welfare Committee.

*Specimen collection, analysis, and quality control*

All blood samples were collected by licensed veterinarians for routine diagnostic purposes under the Veterinary Surgeons Act (1966), following written informed consent by the owners of the dogs. The vast majority of blood samples were collected into plain serum tubes (99.8%) and analysed using an IL600 Clinical Chemistry Analyzer (Instrumentation Laboratory Ltd, Cheshire; UK). Stable performance of the analyzer was monitored by both internal and external quality control and assurance schemes throughout the study period. Reported analytes included total protein, albumin, globulin, sodium, potassium, chloride, calcium, inorganic phosphorus, urea, creatinine, cholesterol, total bilirubin, alanine transferase (ALT), alkaline phosphatase (ALP), creatinine kinase (CK), amylase, lipase and glucose.

*Statistical analysis*

Age of the dogs was classified into eight mutually exclusive categories, namely less than or equal to one year of age (designated ≤1), greater than one year to less than or equal to two years of age (designated >1:≤2), and >2:≤4, >4:≤6, >6:≤8, >8:≤10, and >10 years of age. (For clarity of expression in the text of the Results and Discussion sections, these age categories are respectively labeled in abbreviated form: ≤1, 1‒2, 2‒4, 4‒6, 6‒8, 8‒10 and >10 years.) A linear mixed effects model was used to assess the effect of age, sex, neutering status, and all two-way and three-way interactions on each analyte, taking breed as a random effect. Type I error rate was set at 5%. There were no significant interactions between age and sex, or between age, sex and neutering status, for any of the measured analytes; these interactions were therefore removed from the linear mixed effects model. Residuals were defined as the observed values minus the estimated fixed effects of age, sex, and neutering status. For those breeds represented by at least 10 dogs, principal component analysis (PCA) by best linear unbiased prediction of the random breed effects was undertaken on all but the mixed breed dogs to summarize correlated breed effects on serum biochemical analytes. Those breeds with at least one principal component of less than -2 or greater than +2 were considered to have distinctive phenotypes.

A complementary analytical approach was then adopted to verify the conclusions of PCA. For each biochemical analyte, the distributions of residuals for all breeds represented by at least 10 dogs were compared with those of the mixed breed dogs by two-sample Kolmogorov-Smirnov (KS) tests. Since there were 60 breeds represented by at least 10 dogs, a Bonferroni correction of 0.05/60=8.3 x 10^-4^ was applied. In an effort to increase the rigor of the statistical analysis and further minimize the possibility of a type 1 error inherent in multiple comparisons, a threshold of p=10^-4^ was applied in these analyses. To facilitate the pictorial representation of the data analyzed by KS tests, all breeds were classified into groups previously defined by haplotype analysis of single nucleotide polymorphisms (SNPs) [1], or to their closest matches based on publically available information about breed heritage. These groups were: Ancient, Toy, Working, Sight Hound, Mastiff-like, Retriever/other Mastiff-like, Herding, Terrier, Scent Hound and Spaniel/Pointer (Supplementary Tables 1 to 18). Those breeds not identified in this haplotype analysis and without an obvious nearest match were considered in a miscellaneous group labeled ‘Other’. Data from breeds having values significantly different from the mixed breed control group were displayed in combined box-and-whisker / dot plots.

Where significant differences were identified for breeds with at least 120 individuals, tentative breed-specific reference intervals were calculated based on the ASVCP guidelines [2]. Normality of the analytes were assessed by the Kolmogorov-Smirnov tests and outliers assessed using Horn’s algorithm. Non-parametric methods were employed to establish the 95% reference intervals and the 90% confidence interval of the reference limits. The lower (upper) limit for the 95% breed-specific reference interval per analyte may be overestimated (underestimated) if more than 2% of the recorded data were equal to the existing RVC DL lower (upper) reference limit, thus suggesting ‘truncation’ of the data. This truncation was not applicable to the lower limit for total bilirubin since its value was already 0.

**REFERENCES FOR SUPPORTING INFORMATION**

1. Vonholdt BM, Pollinger JP, Lohmueller KE, Han E, Parker HG, et al. (2010) Genome-wide SNP and haplotype analyses reveal a rich history underlying dog domestication. Nature 464: 898-902.

2. Friedrichs KR, Harr KE, Freeman KP, Szladovits B, Walton RM, et al. (2012) ASVCP reference interval guidelines: determinnation of de novo reference intervals in veterinary species and other related topics. Vet Clin Path 41: 441-453.
